# The influence of high body mass index on asthma: A study integrating GBD 2021 and Mendelian randomization

**DOI:** 10.1097/MD.0000000000051490

**Published:** 2026-11-06

**Authors:** Xiaokun Yao, Chuanli Zhang

**Affiliations:** a Department of Respiratory and Critical Care Medicine, Nanjing Jiangbei Hospital, Nanjing, Jiangsu, China.

**Keywords:** asthma, body mass index, global burden of disease, Mendelian randomization

## Abstract

Elevated body mass index (BMI) represents a significant adjustable risk element for asthma, posing a considerable worldwide health concern. This research was designed to assess the worldwide impact of asthma due to high BMI between 1990 and 2021, forecast patterns through 2050, and use 2-sample Mendelian randomization (MR) to examine whether elevated BMI is genetically associated with asthma risk, in order to guide preventive approaches. The data for this study were obtained from the global burden of disease 2021 study and the integrative epidemiology unit OpenGWAS project. We employed decomposition analysis, Joinpoint regression, and the Bayesian age–period–cohort model to analyze asthma-related mortality and disability-adjusted life years (DALYs) by age, sex, and socio-demographic index region. Additionally, a bidirectional 2-sample MR analysis was performed to investigate the association between genetically predicted BMI and asthma risk. From 1990 to 2021, the global age-standardized mortality rate and age-standardized DALY rate for asthma attributable to high BMI declined. In contrast, the absolute number of deaths and DALYs increased by 63.9% and 45.7%, respectively. The most significant decline occurred between 1996 and 2000, while low-middle socio-demographic index regions experienced consistent increases. Projections to 2050 indicate a continued decline in age-standardized mortality rate but a slight increase in age-standardized DALY rate. Notably, females and older adults carried a higher disease burden. The MR analysis identified a positive, genetically supported association between elevated BMI and asthma risk (inverse-variance weighted odds ratio = 1.389, 95% confidence interval: 1.303–1.479). However, substantial heterogeneity across genetic variants suggests that this estimate should be interpreted with caution. The asthma burden related to high BMI is forecast to increase in the coming decades, mainly fueled by population growth, population aging, and socioeconomic inequalities. MR further supports a unidirectional, genetically supported association between higher BMI and asthma risk. These results highlight the critical necessity for implementing focused and fair public health strategies across the globe to tackle elevated BMI and reduce the associated asthma burden.

## 1. Introduction

Asthma is a complex chronic inflammatory disorder of the airways, leading to recurrent episodes of wheezing and breathlessness.^[1]^ The global burden of disease (GBD) 2021 study reported a global prevalence of around 262 million asthma cases, equating to an age-standardized rate (ASR) of approximately 3340/100,000 people. A critical and paradoxical trend has emerged: Although the age-standardized mortality rate (ASMR) has steadily declined since 1990, the absolute number of individuals living with asthma and dying from it has risen significantly. This escalating burden places a particularly heavy strain on vulnerable groups, including children and older adults.^[2]^ These trends suggest that despite therapeutic advances, medical interventions alone are insufficient to curb the asthma crisis. The pathogenesis of asthma involves a complex interplay of genetic predisposition,^[3]^ environmental exposures,^[4]^ and immune dysregulation.^[5]^

Body mass index (BMI) is a frequently used indicator of obesity and represents a key modifiable risk factor for asthma. According to GBD 2021, a high BMI is a significant risk factor for asthma-related disability-adjusted life years (DALYs) worldwide, accounting for 14.93% of the total disease burden.^[6]^ A large number of observational studies have consistently linked an elevated BMI to an increased risk of asthma.^[7,8]^ Potential biological mechanisms underlying this association include altered airway mechanics, systemic inflammation, metabolic dysfunction, and gut microbiome dysbiosis.^[9]^

Previous GBD-based studies on the asthma burden attributable to a high BMI have mostly focused on older adults,^[10]^ and some relied on the outdated GBD 2019 dataset.^[11]^ Consequently, these studies could not capture recent trends, including the potential impact of the COVID-19 pandemic. This research gap highlights the need for a comprehensive, up-to-date assessment spanning all ages, genders, and socio-demographic index (SDI) categories, and one that integrates genetic evidence from Mendelian randomization (MR). To address these gaps, this study presents the 1st comprehensive analysis of the spatiotemporal trends in high BMI-related asthma burden using the GBD 2021 database. We examined variations by SDI, age, and sex, identified key drivers of burden changes, and projected trends to 2050. Furthermore, to minimize confounding, we performed a 2-sample MR analysis to evaluate the association between genetically predicted BMI and asthma risk. By integrating descriptive epidemiology with MR, this research aims to elucidate the role of a high BMI in driving the evolution of asthma burden and to provide evidence for shaping obesity prevention and global asthma control strategies.

## 2. Methods

### 2.1. Data collection

This analysis used the GBD 2021 dataset to quantify the worldwide burden of asthma resulting from high BMI. The GBD 2021 provides modeled estimates for 371 health conditions and 88 risk factors across geographical scales from national to global.^[12,13]^ From the global health data exchange platform, we retrieved metrics including death numbers, DALYs, and ASRs for high BMI-related asthma. All estimates were obtained with their 95% uncertainty intervals (UIs) for the years 1990 through 2021. The dataset has a global scope, encompassing 204 countries and territories categorized into 21 GBD regions and 5 SDI quintiles. Subgroup analyses were conducted by sex and age group (5-year intervals from 1 to 94 years, plus a category for ≥95 years). Corresponding SDI values were also obtained.

For the MR analysis, summary-level genetic data were sourced from the MRC integrative epidemiology unit (IEU) OpenGWAS database. Genetic instruments for high BMI were obtained from a genome-wide association study (GWAS) of 457,756 individuals of European ancestry^[14]^ (accession: ebi-a-GCST90025994). Summary-level data for asthma were derived from an independent GWAS meta-analysis including 449,500 European-ancestry participants^[15]^ (accession: ebi-a-GCST90018795). Since the exposure (BMI) and outcome (asthma) data came from nonoverlapping studies, sample overlap was assumed to be minimal. The IEU OpenGWAS database was queried with the filters: trait = “body mass index” or “asthma,” population = “European,” and sample size >400,000. The GBD 2021 estimates used in this study are publicly available, de-identified, aggregate-level data that contain no individual-level information. All original GWAS from which we obtained summary-level data had received ethical approval, and participants had provided informed consent. The use of these de-identified summary statistics for the present analysis is considered exempt from additional ethical review.

### 2.2. Definitions

We defined asthma as a persistent respiratory disorder marked by symptoms of bronchospasm and shortness of breath, most commonly triggered by allergic responses. A clinical diagnosis generally necessitates confirmation by a healthcare professional along with a history of wheezing within the previous 12 months. For this study, asthma cases were primarily identified using International Classification of Diseases, 10th revision codes J45 and J46. Supplementary case definitions included self-reported physician diagnosis, self-reported wheezing episodes or asthma within the past year, or any self-reported lifetime asthma.^[16]^ BMI was computed as the ratio of weight (in kilograms) to the square of height (in meters). Individuals aged 20 years or above with a BMI of 25 kg/m^2^ or greater were defined as having a high BMI, in accordance with established criteria.^[17]^ For participants below 20 years of age, we adhered to the age-specific reference criteria set forth by the international obesity task force. The proportion of the asthma burden that could be attributed to high BMI, quantified as the population attributable fraction, was calculated following the comparative risk assessment methodology.^[12]^ This estimation process incorporated 3 key elements: the exposure–response relationship derived from meta-analyses of epidemiological studies, the prevailing global distribution of BMI levels, and the theoretical minimum risk exposure level. DALY is a comprehensive measure that combines years of life lost due to premature mortality with years lived with disability, providing a standardized quantification of overall disease burden. SDI serves as a summary indicator of regional development, synthesized from 3 fundamental components: income per capita, average educational achievement, and the total fertility rate. Within the GBD 2021 analytical framework, all geographical units were stratified into 5 distinct SDI tiers: high (>0.81), high-middle (0.70–0.81), middle (0.61–0.69), low-middle (0.46–0.60), and low (<0.46).

### 2.3. Statistical analysis

The analysis comprised 4 components: burden description, trend analysis, decomposition and prediction, and MR analysis.

#### 2.3.1. Burden description

To facilitate comparisons of the asthma burden by different populations and temporal periods, we relied on ASMR and age-standardized DALY rate (ASDR). These metrics, computed for every 100,000 individuals based on the GBD World Standard Population, adjust for variations in age structure. The standardization follows this calculation:


$$\begin{array}{c} ASR=\frac{\sum_{i=1}^{A}\left(r_{i}\cdot w_{i}\right)}{\sum_{i=1}^{A}w_{i}} \end{array}$$ (1)

$r_{i}$ is the age-specific rate for the ith age group, $w_{i}$ is the corresponding weight in the reference population, and A is the total number of age groups.

The estimated annual percentage change (EAPC) was derived to quantify the temporal trend of the ASR. This metric was obtained by 1st applying a linear regression model to the natural log-transformed rates, defined by the equation: ln(ASR)=α+β·year+ε. The EAPC value was then calculated based on the regression coefficient β using the formula:


EAPC=100×(exp(β)−1) (2)

A trend was considered significantly increasing if both the EAPC estimate and its lower 95% confidence limit were >0, and significantly decreasing if both were <0.

#### 2.3.2. Joinpoint regression analysis

To characterize temporal patterns, we employed Joinpoint regression. This technique models trends by identifying points where the slope changes significantly, calculating the annual percent change (APC) for each segment and the average annual percent change (AAPC) for the entire 1990 to 2021 span, along with their 95% confidence interval (CI). The best-fitting model, allowing for a maximum of 5 joinpoints, was identified by minimizing the akaike information criterion. A permutation test (10,000 replicates; significance threshold *P* < .05) was used to validate the final model selection. An overall trend was considered meaningful if the AAPC was statistically significant (*P* < .05). All Joinpoint analyses were conducted with the Joinpoint Regression Program (Version 5.0.1 from the National Cancer Institute).

#### 2.3.3. Decomposition analysis and prediction analysis

We applied the Das Gupta decomposition method^[18]^ to assess the contributions of population growth, population aging, and changes in epidemiological rates to the observed changes in deaths and DALYs due to high BMI-related asthma. This method is symmetric in that the factor contributions do not depend on the order in which the factors are considered, and no additional standard-population weighting is applied beyond the population counts and rates for the 2 endpoint years.

Projections of the future disease burden for 2022 to 2050 were generated using a Bayesian age–period–cohort (BAPC) model. The model was fitted utilizing the integrated nested Laplace approximation technique,^[19]^ which offers computational advantages and avoids convergence problems common with conventional Markov Chain Monte Carlo sampling. Temporal smoothing was ensured by incorporating 2nd-order random walk (RW2) priors for the age, period, and cohort effects, following the default specification of the BAPC framework. RW2 priors allow locally linear trends to be learned from the data while penalizing excessive volatility, and are therefore well-suited to capturing nonlinear temporal dynamics. The model’s predictive performance was evaluated by training it on data from 1990 to 2010 and forecasting the period 2011 to 2021. The strong concordance between the projected estimates and the actual observed values during this validation period provided confidence in the model’s forecasts up to 2050. In sensitivity analyses, all 4 sex- and measure-specific models were refitted with 1st-order random walk (RW1) priors to assess the robustness of the projections to the prior specification.

#### 2.3.4. MR analysis

A bidirectional 2-sample MR analysis was employed to evaluate the association between genetically predicted BMI and asthma risk. This analysis rests on 3 key instrumental variable assumptions^[20]^: relevance: genetic instruments must be strongly associated with BMI; independence: instruments are independent of confounders affecting the BMI–asthma relationship; and exclusion restriction: instruments influence asthma risk only via BMI, not through alternative pathways. Genetic instruments for BMI were selected as single-nucleotide polymorphisms (SNPs) reaching genome-wide significance (*P* < 5 × 10^−8^) from a large-scale GWAS of 457,756 individuals of European ancestry.^[14]^ To ensure independence, linkage disequilibrium (LD) clumping was performed (window size = 10,000 kb, *r*^2^ < 0.001). SNPs associated with asthma (*P* < 5 × 10^−8^) were excluded to minimize pleiotropy. This process yielded 368 independent SNPs, of which 361 were retained after harmonization as final instruments. All MR analyses were performed with the TwoSampleMR R package using summary-level data and the European LD reference panel from the OpenGWAS database. The strength of these instruments was confirmed, with all SNP-specific *F* statistics exceeding 10 (mean *F* statistic = 68.4, minimum = 28.5; Table S1, Supplemental Digital Content 1), indicating minimal weak instrument bias. The primary estimate was derived using the inverse-variance weighted (IVW) method. To evaluate robustness, we conducted sensitivity analyses including MR-Egger regression, the weighted median method, the weighted mode and simple mode estimators, Cochran’s *Q* test for heterogeneity, the MR-Pleiotropy Residual Sum and Outlier (MR-PRESSO) test, and leave-one-out analysis. The MR-PRESSO test was implemented using the MR-PRESSO R package, with 10,000 Monte Carlo simulations performed to ensure the precision of the empirical *P* values of the global, outlier, and distortion tests (for 361 instruments, at least 7220 simulations – 20 per instrument – are required to reach the nominal significance level of 0.05 for the outlier test). MR-Egger regression further relies on the InSIDE assumption (i.e., that instrument strength is independent of the direct pleiotropic effects), a condition that cannot be directly tested in summary-data MR and whose violation may bias the MR-Egger estimate.

In order to evaluate the possibility of reverse causality, we conducted a reverse MR analysis with asthma as the exposure and BMI as the outcome, using the same GWAS summary datasets.^[14,15]^ We identified 67 independent SNPs associated with asthma at genome-wide significance (*P* < 5 × 10^−8^), applying the same LD clumping criteria and quality control procedures as in the forward analysis. After harmonization and proxy-based lookup, 48 instruments were retained. These instruments were robust (mean *F* statistic = 65.5, minimum = 29.9), substantially exceeding the recommended threshold of 10, thus mitigating concerns of weak instrument bias. All MR analyses followed the Strengthening the Reporting of Observational Studies in Epidemiology for Mendelian Randomization (STROBE-MR) guidelines.^[21]^

### 2.4. Statistical statement

The reported values for all GBD-derived estimates correspond to the mean of 1000 draws from the posterior distribution. The 95% UIs are defined by the 2.5th and 97.5th percentiles of these posterior draws. All rates are presented per 100,000 people. For the MR analysis, results are reported as odds ratios (ORs) accompanied by 95% CIs. A 2-sided *P* value below .05 served as the threshold for determining statistical significance. The statistical computing environment R (version 4.3.2; R Foundation for Statistical Computing, Vienna, Austria) was used for all analyses, leveraging key packages such as ggplot2, JoinpointR, BAPC, and TwoSampleMR (version 0.7.11). This study is reported in accordance with the STROBE-MR guidelines for Mendelian randomization studies and the Guidelines for Accurate and Transparent Health Estimates Reporting statement for studies using GBD data and global health estimates; a completed STROBE-MR checklist accompanies this submission, and the completed Guidelines for Accurate and Transparent Health Estimates Reporting checklist is available from the corresponding author upon reasonable request.

## 3. Results

### 3.1. Global burden of high BMI-associated asthma

A steady decline was observed in the global ASMR and ASDR for high BMI-related asthma between 1990 and 2021. The ASMR decreased from 1.00 (95% UI: 0.43–1.58) to 0.74 (95% UI: 0.31–1.19)/100,000, with an EAPC of −1.13 (95% CI: −1.23 to −1.04). Similarly, the ASDR fell from 50.53 (95% UI: 24.72–78.10) to 39.42 (95% UI: 19.62–60.16)/100,000, corresponding to an EAPC of −0.85 (95% CI: −0.96 to–0.73) (Tables 1 and 2; Fig. 1).

**Table 1:** Number of asthma deaths attributable to high BMI across SDI and GBD regions in 1990 and 2021, with EAPC (1990–2021).

Location	1990		2021		1990–2021 EAPC (95% CI)
	Deaths cases (95% UI)	ASMR per 100,000 (95% UI)	Deaths cases (95% UI)	ASMR per 100,000 (95% UI)	
Global	38,040.53 (16,523.50–59,933.15)	1.00 (0.43–1.58)	62,363.20 (26,317.27–100,543.22)	0.74 (0.31–1.19)	−1.13 (−1.23 to −1.04)
High SDI	7721.07 (3386.96–12,129.70)	0.70 (0.31–1.10)	4621.61 (2149.42–7199.33)	0.23 (0.11–0.36)	−4.17 (−4.58 to −3.76)
High-middle SDI	6889.73 (2972.03–10,695.72)	0.74 (0.32–1.15)	5492.92 (2547.26–8682.38)	0.29 (0.13–0.46)	−3.66 (−3.93 to −3.38)
Middle SDI	9485.31 (4099.26–14,783.97)	1.05 (0.44–1.64)	18,831.86 (8563.15–29,760.47)	0.75 (0.34–1.19)	−1.29 (−1.43 to −1.15)
Low-middle SDI	9846.35 (4201.12–16,192.68)	1.73 (0.71–2.91)	24,557.84 (10,083.79–41,567.56)	1.83 (0.76–3.17)	0.36 (0.28–0.43)
Low SDI	4040.16 (1743.67–6759.75)	1.83 (0.74–3.15)	8798.36 (3533.27–15,604.21)	1.80 (0.69–3.36)	−0.01 (−0.07 to 0.06)
Andean Latin America	132.33 (69.98–200.41)	0.56 (0.26–0.88)	108.98 (52.06–181.49)	0.19 (0.09–0.31)	−3.60 (−3.73 to −3.46)
Australasia	213.62 (93.54–333.90)	0.94 (0.41–1.46)	173.87 (81.57–265.78)	0.33 (0.16–0.50)	−3.44 (−4.14 to −2.72)
Caribbean	251.35 (115.34–393.19)	0.93 (0.42–1.47)	390.98 (175.52–651.07)	0.74 (0.34–1.23)	−1.01 (−1.23 to −0.78)
Central Asia	1191.66 (508.63–1913.06)	2.66 (1.13–4.28)	909.30 (416.8–1422.7)	1.21 (0.56–1.90)	−3.36 (−3.91 to −2.81)
Central Europe	1631.30 (720.75–2562.25)	1.16 (0.51–1.83)	543.51 (253.91–840.91)	0.23 (0.11–0.36)	−5.77 (−6.27 to −5.27)
Central Latin America	961.92 (438.72–1485.49)	1.25 (0.56–1.96)	746.94 (353.35–1155.60)	0.30 (0.14–0.47)	−5.06 (−5.26 to −4.86)
Central Sub-Saharan Africa	450.62 (173.88–999.34)	2.07 (0.71–5.32)	1330.02 (499.65–3237.49)	2.61 (0.93–7.04)	0.69 (0.64–0.75)
East Asia	3261.83 (1355.18–5477.72)	0.50 (0.21–0.89)	4685.71 (2125.59–7652.26)	0.25 (0.11–0.41)	−2.60 (−2.81 to −2.40)
Eastern Europe	2689.43 (1194.46–4200.68)	0.97 (0.43–1.52)	485.89 (218.92–737.45)	0.14 (0.06–0.21)	−7.60 (−8.05 to −7.14)
Eastern Sub-Saharan Africa	1012.12 (467.84–1615.22)	1.23 (0.51–2.18)	2171.97 (874.13–4256.50)	1.21 (0.46–2.50)	−0.22 (−0.27 to −0.17)
High-income Asia Pacific	1357.11 (589.22–2172.43)	0.75 (0.32–1.20)	603.86 (257.68–1002.11)	0.10 (0.04–0.16)	−7.84 (−8.30 to −7.37)
High-income North America	1393.70 (628.62–2158.79)	0.41 (0.19–0.63)	1310.45 (619.12–1970.94)	0.25 (0.12–0.36)	−2.32 (−2.64 to −1.99)
North Africa and Middle East	4757.80 (2044.36–7554.52)	3.20 (1.30–5.16)	7434.99 (3348.7–11,615.24)	1.90 (0.84–2.98)	−1.90 (−2.01 to −1.78)
Oceania	184.78 (76.27–317.46)	7.50 (3.07–12.9)	405.23 (179.81–724.63)	6.10 (2.65–10.88)	−0.83 (−0.91 to −0.75)
South Asia	7811.68 (3153.74–13,671.81)	1.45 (0.56–2.62)	25,159.69 (10,125.98–45,192.89)	1.84 (0.74–3.35)	1.02 (0.88–1.16)
Southeast Asia	3319.88 (1470.17–5441.3)	1.36 (0.57–2.24)	7725.09 (3508.72–12,451.87)	1.26 (0.57–2.03)	−0.33 (−0.47 to −0.18)
Southern Latin America	268.38 (122.27–420.96)	0.59 (0.27–0.92)	231.61 (106.39–355.11)	0.26 (0.12–0.40)	−2.87 (−3.18 to −2.57)
Southern Sub-Saharan Africa	1017.79 (429.43–1660.64)	3.72 (1.54–6.26)	2080.10 (958.27–3231.05)	3.82 (1.75–6.02)	−0.01 (−0.55 to 0.54)
Tropical Latin America	464.47 (219.10–734.04)	0.53 (0.24–0.84)	726.52 (322.91–1135.6)	0.29 (0.13–0.45)	−2.55 (−2.89 to −2.21)
Western Europe	4000.07 (1753.57–6241.17)	0.69 (0.31–1.08)	1818.05 (788.13–2841.27)	0.18 (0.08–0.27)	−4.87 (−5.36 to −4.37)
Western Sub-Saharan Africa	1668.68 (729.20–2743.12)	1.96 (0.83–3.25)	3320.41 (1516.38–5485.21)	1.73 (0.77–2.86)	−0.35 (−0.42 to −0.28)

ASMR = age-standardized mortality rate, BMI = body mass index, CI = confidence interval, EAPC = estimated annual percentage change, GBD = global burden of disease, SDI = socio-demographic index, UI = uncertainty interval.

**Table 2:** Number of asthma DALYs attributable to high BMI across SDI and GBD regions in 1990 and 2021, with EAPC (1990–2021).

Location	1990		2021		1990–2021
	DALYs cases (95% UI)	ASDR per 100,000 (95% UI)	DALYs cases (95% UI)	ASDR per 100,000 (95% UI)	EAPC (95% CI)
Global	2,253,937.15 (1,125,455.31–3,432,735.85)	50.53 (24.72–78.10)	3,283,295.51 (1,620,702.48–5,034,163.58)	39.42 (19.62–60.16)	−0.85 (−0.96 to −0.73)
High SDI	757,074.07 (353,890.12–1,210,548.27)	76.66 (36.67–121.16)	775,180.58 (367,112.27–1,214,966.43)	62.10 (30.61–95.22)	−0.48 (−0.74 to −0.22)
High-middle SDI	447,609.14 (211,179.44–701,354.98)	43.40 (20.60–67.87)	392,010.78 (191,778.25–602,254.99)	25.40 (12.75–39.05)	−2.15 (−2.36 to −1.94)
Middle SDI	466,571.37 (240,623.53–700,453.43)	36.88 (18.02–55.64)	830,967.74 (419,114.10–1,237,330.69)	32.06 (16.57–47.76)	−0.65 (−0.73 to −0.57)
Low-middle SDI	397,134.25 (202,607.94–598,244.35)	52.89 (24.28–82.79)	874,703.70 (398,330.18–1,358,092.02)	55.50 (24.53–88.23)	0.25 (0.20–0.29)
Low SDI	181,911.24 (97,421.60–275,769.08)	58.99 (26.95–93.07)	406,690.78 (213,266.51–641,050.81)	58.28 (26.47–96.83)	−0.10 (−0.14 to −0.06)
Andean Latin America	17,238.99 (10,189.80–26,106.41)	44.30 (26.75–66.86)	19,331.94 (11,042.44–29,735.15)	29.97 (17.16–46.10)	−1.33 (−1.53 to −1.12)
Australasia	24,391.30 (11,685.73–38,405.11)	112.95 (54.91–176.91)	25,500.35 (12,188.05–40,315.43)	76.14 (37.92–119.92)	−1.51 (−1.68 to −1.35)
Caribbean	21,652.97 (11,361.17–32,772.49)	66.91 (34.65–101.78)	31,076.52 (16,436.16–46,626.69)	63.90 (33.83–96.30)	−0.33 (−0.41 to −0.25)
Central Asia	43,276.76 (20,574.56–66,272.22)	85.50 (39.26–131.67)	41,855.78 (21,245.90–63,471.71)	47.44 (24.10–71.81)	−2.69 (−3.09 to −2.29)
Central Europe	110,455.93 (50,928.07–176,204.22)	76.72 (35.91–122.34)	62,873.28 (29,628.84–99,050.20)	43.43 (21.73–67.69)	−2.00 (−2.28 to −1.72)
Central Latin America	65,091.23 (36,326.00–96,695.04)	53.63 (28.29–80.74)	73,529.27 (39,973.23–111,005.35)	29.05 (15.92–43.67)	−2.23 (−2.39 to −2.06)
Central Sub-Saharan Africa	19,527.25 (9498.27–35,544.94)	62.32 (26.79–132.4)	57,999.64 (28,769.55–112,495.35)	75.95 (30.95–167.77)	0.55 (0.49–0.61)
East Asia	164,172.04 (79,221.47–260,603.12)	17.46 (8.22–27.24)	238,468.42 (117,198.73–369,127.75)	14.18 (7.25–21.82)	−0.94 (−1.15 to −0.74)
Eastern Europe	163,639.00 (75,450.37–254,520.57)	61.01 (28.63–94.95)	51,958.62 (25,115.5–80,536.79)	21.34 (10.59–33.50)	−4.19 (−4.42 to −3.96)
Eastern Sub-Saharan Africa	63,428.00 (36,946.34–95,211.81)	48.37 (25.04–73.07)	139,551.80 (77,598.72–219,047.11)	48.14 (22.82–82.59)	−0.19 (−0.24 to −0.14)
High-income Asia Pacific	86,021.23 (40,222.88–138,714.29)	45.15 (21.63–73.03)	38,688.94 (18,017.53–61,778.99)	15.92 (8.11–25.25)	−3.90 (−4.18 to −3.62)
High-income North America	243,944.08 (116,634.79–382,442.25)	81.44 (40.39–125.56)	408,798.47 (196,560.69–636,692.14)	98.56 (48.43–150.97)	1.23 (0.94–1.53)
North Africa and Middle East	208,518.43 (104,743.95–310,987.91)	101.45 (47.21–154.49)	370,701.22 (194,053.79–551,716.07)	71.41 (37.10–107.87)	−1.37 (−1.46 to −1.28)
Oceania	7035.41 (3424.09–11,639.31)	197.64 (90.12–328.94)	14,594.91 (6810.68–25,175.63)	162.79 (73.99–285.55)	−0.75 (−0.82 to −0.68)
South Asia	283,892.79 (130,790.74–462,505.45)	41.85 (18.35–70.47)	776,006.01 (327,108.28–1,289,399.31)	49.83 (20.64–83.93)	0.76 (0.67–0.86)
Southeast Asia	139,980.52 (69,272.39–215,893.99)	43.98 (21.26–68.59)	297,830.98 (136,294.39–459,609.87)	42.96 (20.05–66.78)	−0.12 (−0.25 to 0.01)
Southern Latin America	33,497.59 (15,942.33–53,237.56)	70.26 (33.30–112.19)	46,500.94 (21,950.12–73,830.08)	61.93 (29.78–96.74)	−0.65 (−0.73 to −0.57)
Southern Sub-Saharan Africa	40,744.50 (19,884.37–63,289.91)	116.52 (52.92–187.94)	70,676.87 (33,316.06–106,853.52)	107.94 (50.65–163.83)	−0.33 (−0.83 to 0.18)
Tropical Latin America	57,686.36 (31,899.43–87,145.26)	42.63 (22.09–65.57)	84,032.58 (44,303.00–127,141.38)	37.47 (20.79–56.4)	−0.91 (−1.1 to −0.72)
Western Europe	385,880.24 (176,641.42–630,814.90)	81.52 (37.96–132.10)	256,877.51 (116,217.56–420,697.80)	49.71 (23.14–80.19)	−1.58 (−1.78 to −1.38)
Western Sub-Saharan Africa	73,862.54 (38,301.15–114,326.90)	64.57 (30.62–102.52)	176,441.47 (89,866.92–268,632.09)	60.72 (28.99–93.97)	−0.21 (−0.27 to −0.15)

ASDR = age-standardized DALY rate, BMI = body mass index, CI = confidence interval, DALYs = disability-adjusted life years, EAPC = estimated annual percentage change, GBD = global burden of disease, SDI = socio-demographic index, UI = uncertainty interval.

**Figure 1.**
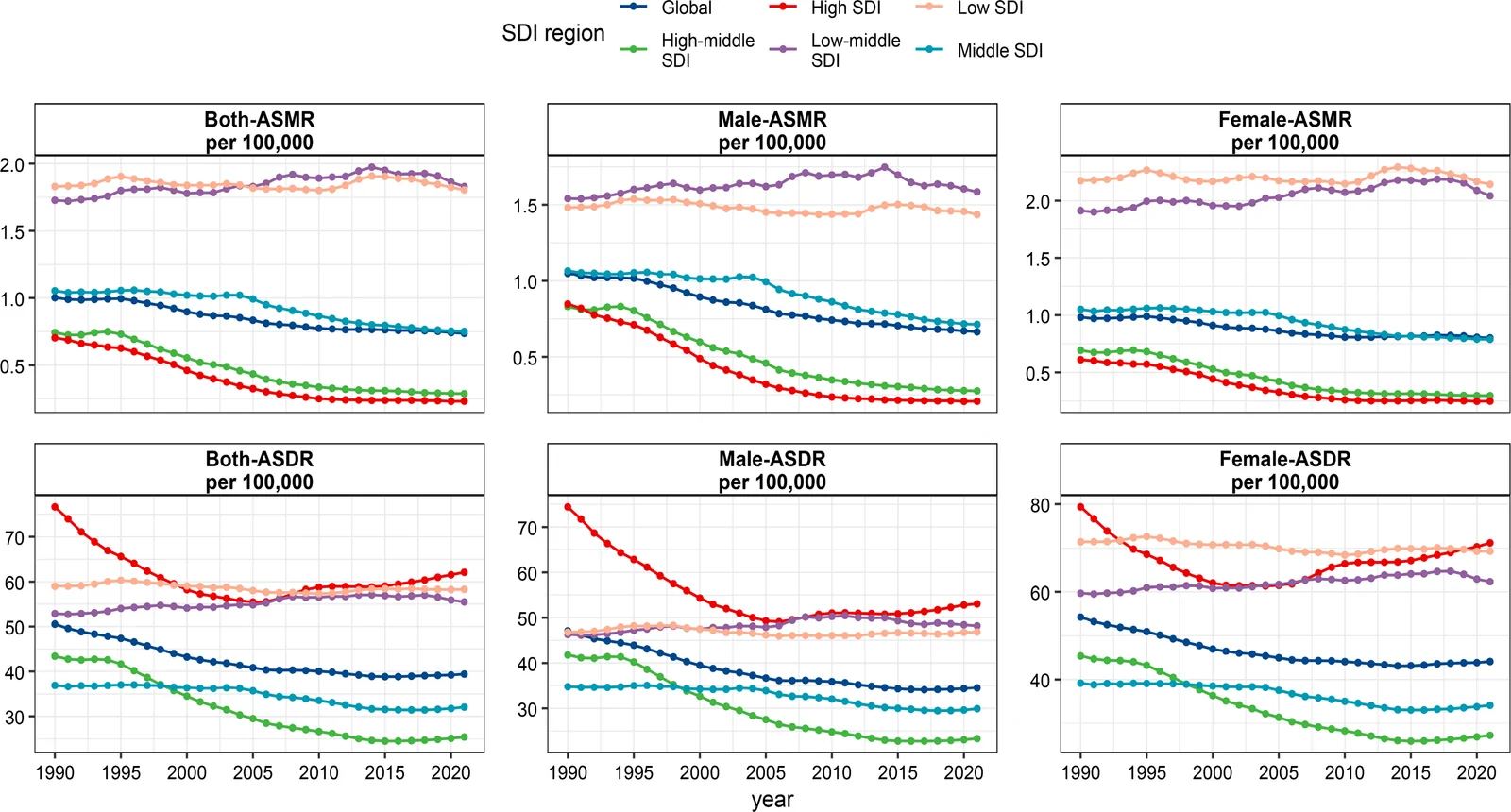
Temporal patterns in ASMR and ASDR for asthma attributable to high BMI, stratified by sex and SDI region, 1990 to 2021. ASDR = age-standardized disability-adjusted life years rate, ASMR = age-standardized mortality rate, BMI = body mass index, SDI = socio-demographic index.

Conversely, the absolute number of asthma deaths attributable to high BMI increased by 63.94%, from 38,040.53 (95% UI: 16,523.50–59,933.15) in 1990 to 62,363.20 (95% UI: 26,317.27–100,543.22) in 2021 (Table 1). Similarly, DALYs increased by 45.67%, from 2,253,937 (95% UI: 1,125,455–3,432,736) to 3,283,296 (95% UI: 1,620,702–5,034,164) over the same period (Table 2).

### 3.2. Regional burden of high BMI-associated asthma

The low-middle SDI region recorded the greatest absolute number of asthma-related deaths (24,557.84) and DALYs (874,703.70) associated with high BMI in 2021 (Tables 1 and 2). A distinctive pattern was observed in this region, which was the only SDI category to record concurrent increases in both ASMR and ASDR throughout the period (1990–2021; Fig. 1). In stark contrast, all other SDI regions exhibited universal declining trends. The most pronounced decrease in ASMR occurred in the high SDI region, while the greatest reduction in ASDR was seen in the high-middle SDI region (Fig. 1).

Across the 21 GBD regions, South Asia accounted for the largest share of asthma deaths (25,159.69; 95% UI: 10,125.98–45,192.89) and DALYs (776,006.01; 95% UI: 327,108.28–1,289,399.31) caused by high BMI in 2021 (Tables 1 and 2; Fig. 2A and B). A different pattern emerged upon examining ASRs: Oceania presented the most severe burden globally, with an ASMR of 6.10 and an ASDR of 162.79/100,000 population (Tables 1 and 2; Fig. 2C and D). An analysis of temporal trends from 1990 to 2021 revealed that high-income Asia Pacific experienced the steepest mortality rate reduction (EAPC = −7.84), whereas Eastern Europe showed the most rapid decline in DALY rates (EAPC = −4.19). On the other end of the spectrum, South Asia displayed the most substantial upward trend in ASMR (EAPC = 1.02), and high-income North America led in ASDR increases (EAPC = 1.23) (Tables 1 and 2).

**Figure 2.**
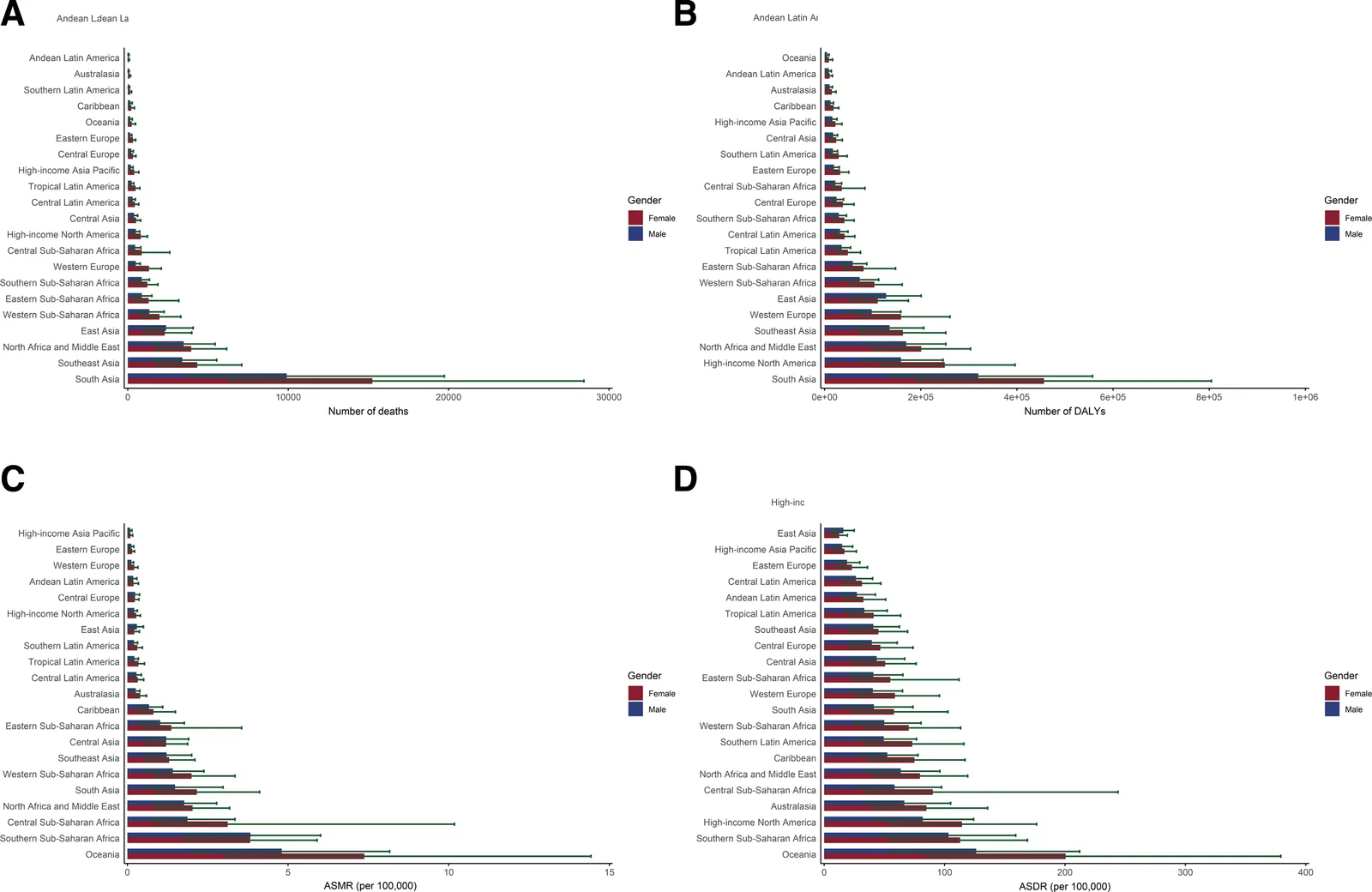
Distribution of the asthma burden associated with high BMI across the 21 GBD regions, 2021. (A) Number of deaths. (B) Number of DALYs. (C) ASMR. (D) ASDR. ASDR = age-standardized DALY rate, ASMR = age-standardized mortality rate, BMI = body mass index, DALYs = disability-adjusted life years, GBD = global burden of disease.

### 3.3. National burden of high BMI-associated asthma

In 2021, India recorded the highest number of asthma deaths attributable to high BMI (21,018.30; 95% UI: 8407.39–36,835.71), followed by China (4321.91; 95% UI: 1949.72–7146.99) and Indonesia (3231.94; 95% UI: 1439.41–5316.53) (Table S2, Supplemental Digital Content 2). A comparable trend was noted in relation to DALYs, with India again having the highest burden (632,587.57; 95% UI: 261,685.39–1,046,890.64), followed by the United States (389,253.62; 95% UI: 187,273.68–606,143.36) and China (218,662.32; 95% UI: 107,327.86–339,079.19) (Table S3, Supplemental Digital Content 3). Regarding ASRs, Fiji recorded the greatest ASMR (12.84/100,000; 95% UI: 5.96–20.04) and ASDR (285.03/100,000; 95% UI: 138.37–435.79) globally in 2021, with Kiribati ranking 2nd (Fig. 3A and B; Tables S2 and S3, Supplemental Digital Content 2). A longitudinal analysis from 1990 to 2021 identified 26 countries with rising ASMR trends. Additionally, 41 countries showed increasing ASDR. Among the countries with rising trends, Zimbabwe stood out with the sharpest increases in both ASMR and ASDR, exhibiting EAPCs of 2.97 (95% CI: 2.56–3.38) and 3.01 (95% CI: 2.55–3.48) (Tables S2 and S3, Supplemental Digital Content 2).

**Figure 3.**
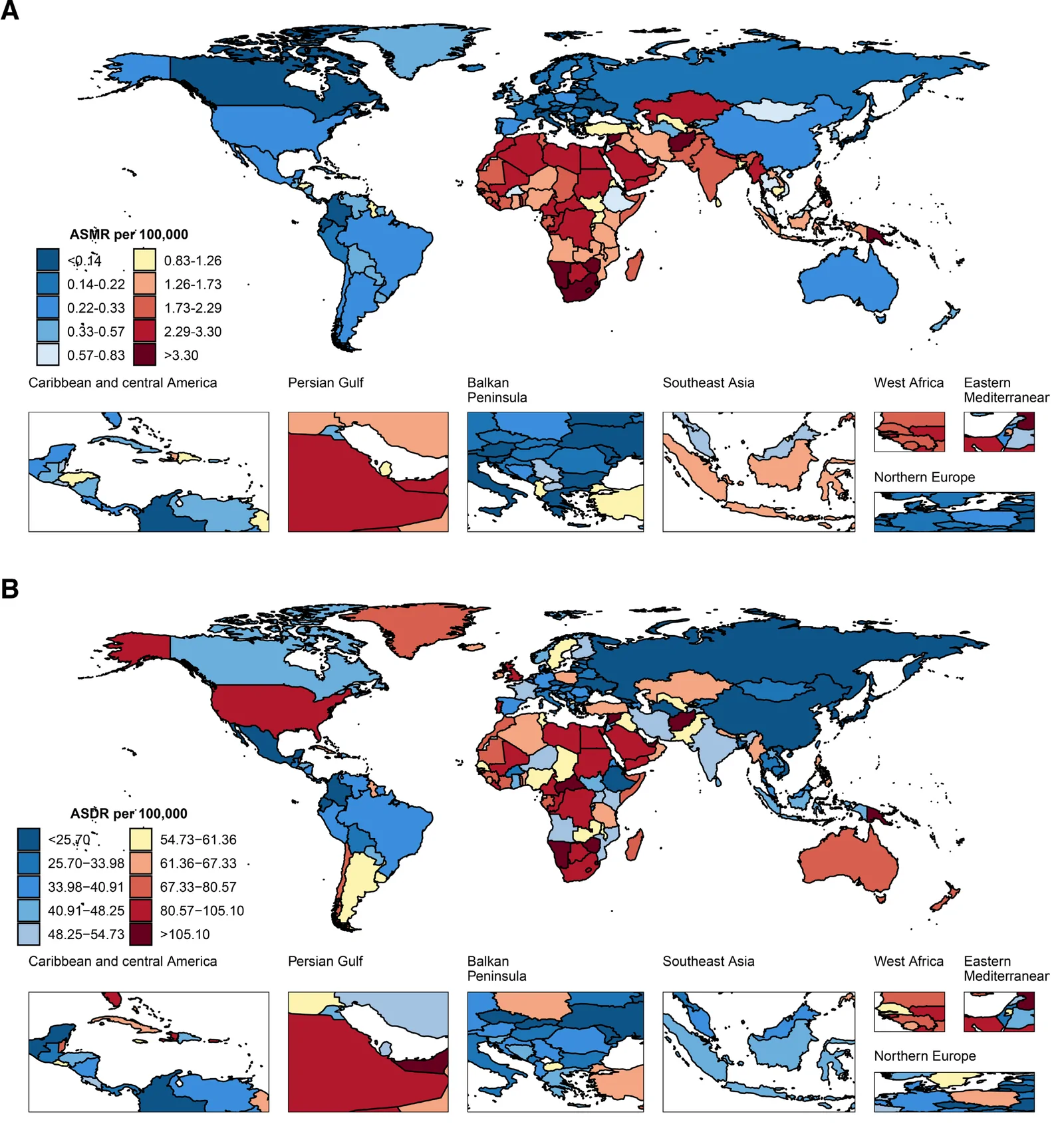
Geographical profile of age-standardized asthma rates attributable to high BMI across nations, 2021. (A) ASMR. (B) ASDR. ASDR = age-standardized DALY rate, ASMR = age-standardized mortality rate, BMI = body mass index.

### 3.4. High BMI-associated asthma burden stratified by age group and sex

The distribution of asthma deaths associated with high BMI in 2021 exhibited a clear age-dependent pattern (Fig. 4A). The number of deaths was relatively low during childhood, then gradually increased with advancing age. Both men and women reached their peak in the 70 to 74 age group, and then it decreased again. A consistent sex disparity was apparent throughout the lifespan, with female death numbers persistently surpassing those of males in every age group. When examining mortality rates, a steady age-related increase was observed, culminating in the highest values among women aged 95 years and above. For men, the mortality rate maximized in the 90 to 94 year age group before stabilizing.

**Figure 4.**
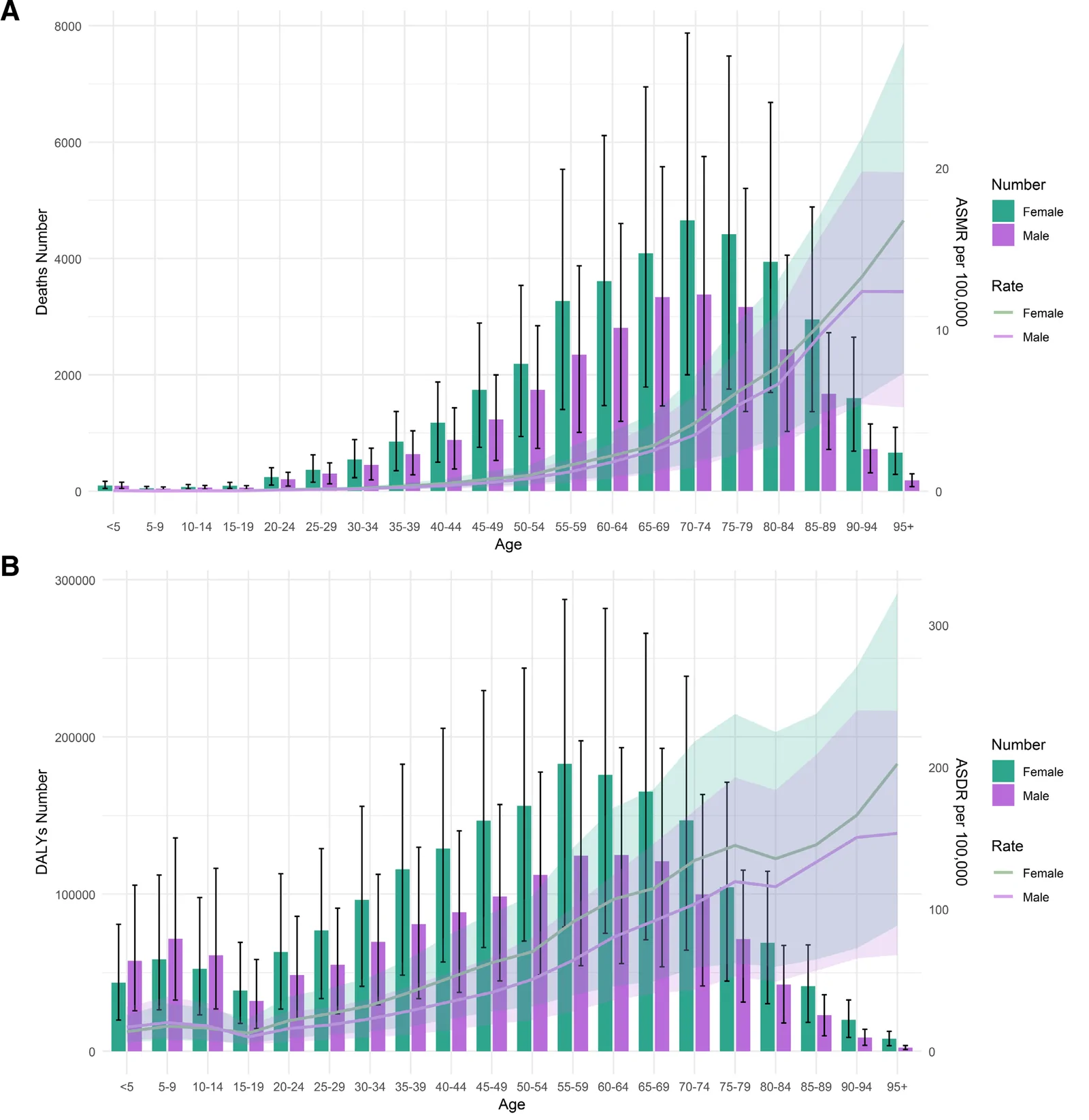
Global burden of asthma attributable to high BMI in 2021, stratified by age group and gender. (A) Number of deaths and ASMR. (B) Number of DALYs and ASDR. ASDR = age-standardized DALY rate, ASMR = age-standardized mortality rate, BMI = body mass index, DALYs = disability-adjusted life years.

An analogous profile was evident for DALYs (Fig. 4B). The number of DALYs decreased from ages 5 to 19 before gradually increasing to their maximum. The peak occurred in the 60 to 64 age group for men and the 55 to 59 age group for women, followed by a subsequent decline. A notable sex-specific crossover was identified: while males below 15 years of age exhibited greater DALY numbers compared to females, this pattern reversed after age 15, from which point females shouldered a progressively larger proportion of the DALY burden. The ASDR demonstrated a near-linear ascending trajectory from ages 15 to 79 for both males and females.

### 3.5. Correlation of SDI with high BMI-associated asthma burden

A significant inverse relationship was identified between the SDI and ASMR of asthma attributable to high BMI, both globally and across the 21 GBD regions from 1990 to 2021 (ρ = −0.70, *P* < .001; Fig. 5A). Higher SDI was generally associated with a lower mortality burden. A weaker, but still significant, inverse correlation was found between SDI and ASDR (ρ = −0.12, *P* < .001; Fig. S1A, Supplemental Digital Content 4). Notably, regions including Oceania and sub-Saharan Africa exhibited ASMR and ASDR values that markedly exceeded the expectations based on their SDI levels.

**Figure 5.**
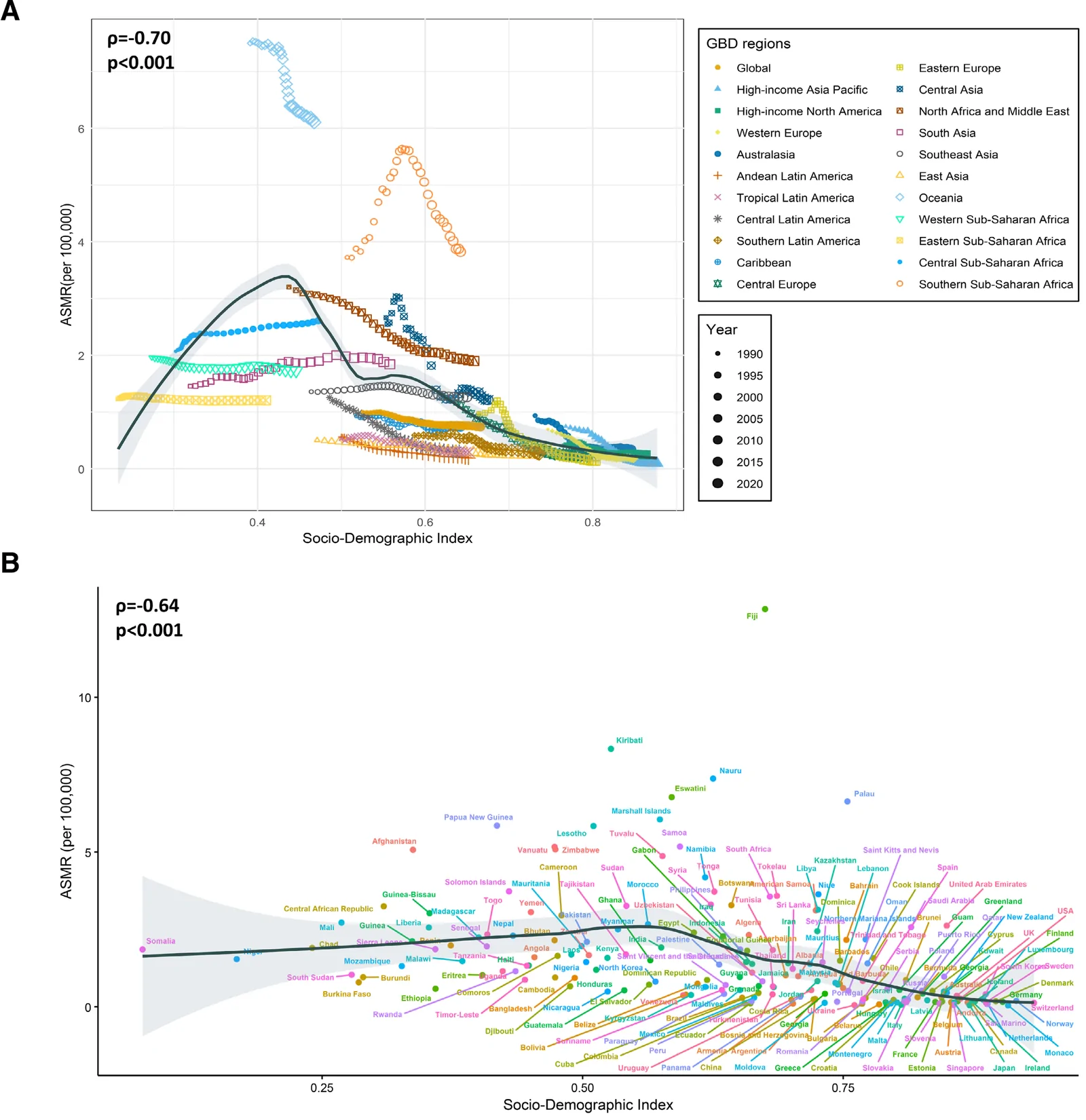
Association between the SDI and ASMR of asthma attributable to high BMI. (A) Longitudinal trends across 21 GBD regions, 1990–2021. (B) Cross-national variation in 204 countries and territories, 2021. ASDR = age-standardized disability-adjusted life years rate, ASMR = age-standardized mortality rate, BMI = body mass index, GBD = global burden of disease, SDI = socio-demographic index.

This inverse association persisted at the national level in 2021 for both ASMR (ρ = −0.64, *P* < .001; Fig. 5B) and ASDR (ρ = −0.25, *P* < .001; Fig. S1B, Supplemental Digital Content 4). Fiji was a notable outlier, with observed rates far exceeding the predictions based on its SDI.

### 3.6. Joinpoint regression analysis of high BMI-associated asthma burden

Joinpoint analysis for the period 1990 to 2021 revealed an overall declining trend in the global ASMR (AAPC = −0.97) and ASDR (AAPC = −0.79) for asthma attributable to high BMI. The most substantial decline in ASMR was recorded in the 1996 to 2000 interval (APC = −2.29), after which the rate of decline decelerated (Fig. 6A). A similar pattern was observed for ASDR, with the steepest decrease from 1996 to 2000 (APC = –1.90), followed by a slower rate of decline. Of note, the ASDR exhibited a slight but significant increase between 2015 and 2021 (APC = 0.2, *P* < .05; Fig. 6B).

**Figure 6.**
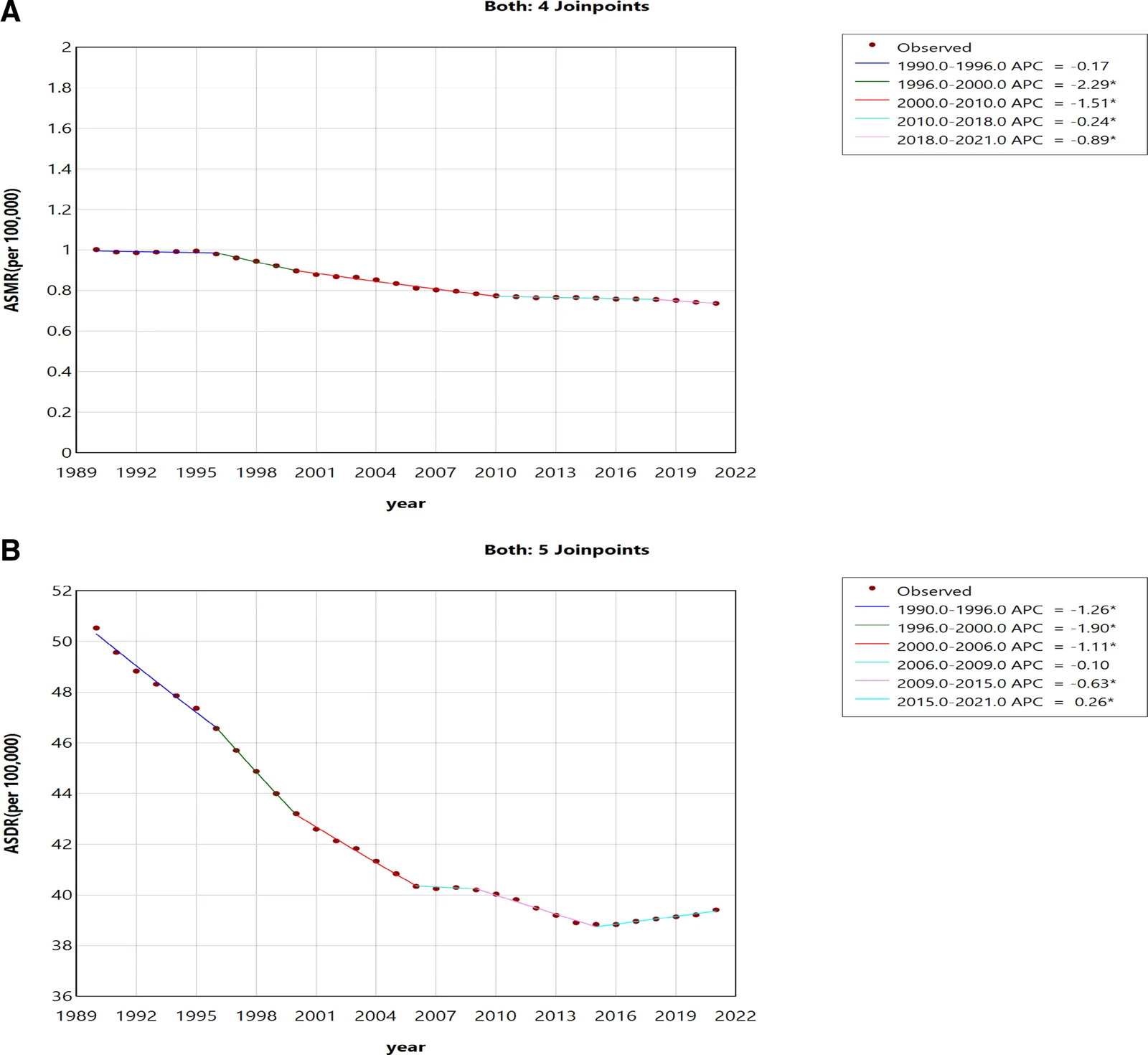
Joinpoint trend analysis of the global asthma burden associated with high BMI, 1990 to 2021. (A) ASMR. (B) ASDR. APC = annual percent change, ASDR = age-standardized disability-adjusted life years rate, ASMR = age-standardized mortality rate, BMI = body mass index.

### 3.7. Decomposition analysis of high BMI-associated asthma burden

Decomposition analysis indicated that the increases in deaths and DALYs from high BMI-related asthma between 1990 and 2021 were primarily driven by population growth and population aging, while the overall contribution of changes in epidemiological rates was relatively modest (Fig. 7; Table S4, Supplemental Digital Content 5).

**Figure 7.**
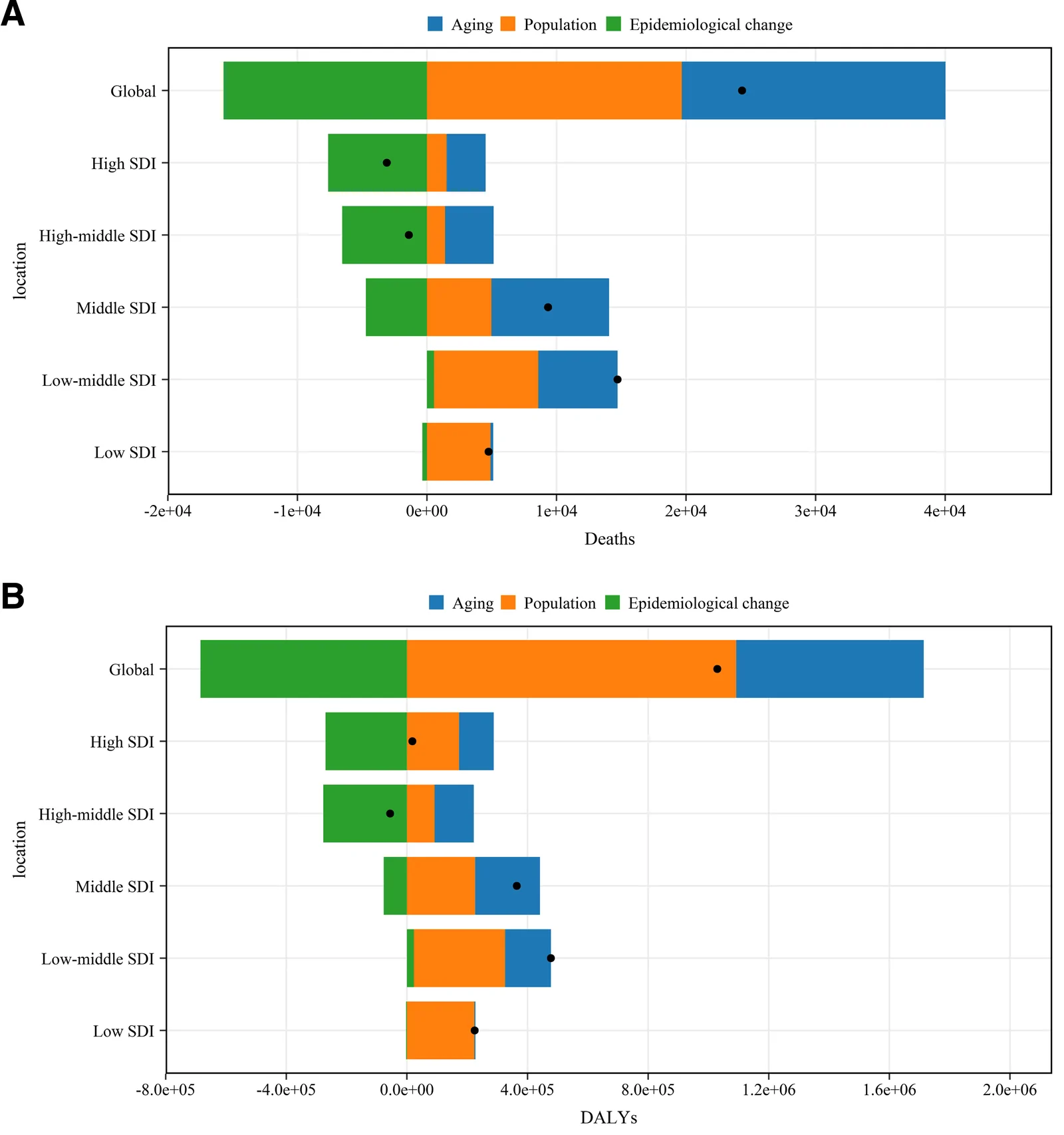
Decomposition analysis of the trends in asthma-related deaths and DALYs associated with high BMI, by SDI region. (A) Deaths. (B) DALYs. BMI = body mass index, DALYs = disability-adjusted life years, SDI = socio-demographic index.

Notable disparities were observed across SDI regions. High and high-middle SDI regions experienced a net decrease in deaths, largely due to favorable epidemiological changes that reduced death rates, especially in high-SDI regions. In middle SDI and low SDI regions, although epidemiological changes also contributed to risk reduction, this effect was more than offset by the strong positive contributions from population growth and aging, resulting in a net increase in deaths (Fig. 7A). Trends for DALYs broadly mirrored those for mortality (Fig. 7B), with the exception that high-SDI regions showed a small net increase in DALYs despite a net decrease in deaths, driven by the overwhelming contributions of population growth and aging. It is important to highlight that unfavorable epidemiological changes, which increased the burden of asthma-related deaths and DALYs, were observed exclusively in the low-middle SDI regions (Fig. 7).

### 3.8. Prediction of high BMI-associated asthma burden

The BAPC model was employed to project the high BMI-attributable asthma burden through the year 2050. Globally, a steady decline in ASMR is forecast for both males and females throughout this period (Fig. 8). The decline is expected to be slightly faster in females than in males. By 2050, the ASMR is predicted to be 0.60/100,000 individuals for males and 0.65/100,000 individuals for females (Fig. 8A and B). In contrast, the ASDR is forecast to increase modestly. By 2050, the male ASDR is expected to reach 43.40/100,000, and the female ASDR is projected to be 54.68/100,000 (Fig. 8C and D). These projections indicate that females will likely continue to experience a disproportionately higher burden in the future. In sensitivity analyses, refitting all 4 sex- and measure-specific models with RW1 priors yielded materially different 2022 to 2050 trajectories for some outcomes (Fig. S2, Supplemental Digital Content 6). The RW2-based projections were therefore retained as the primary results.

**Figure 8.**
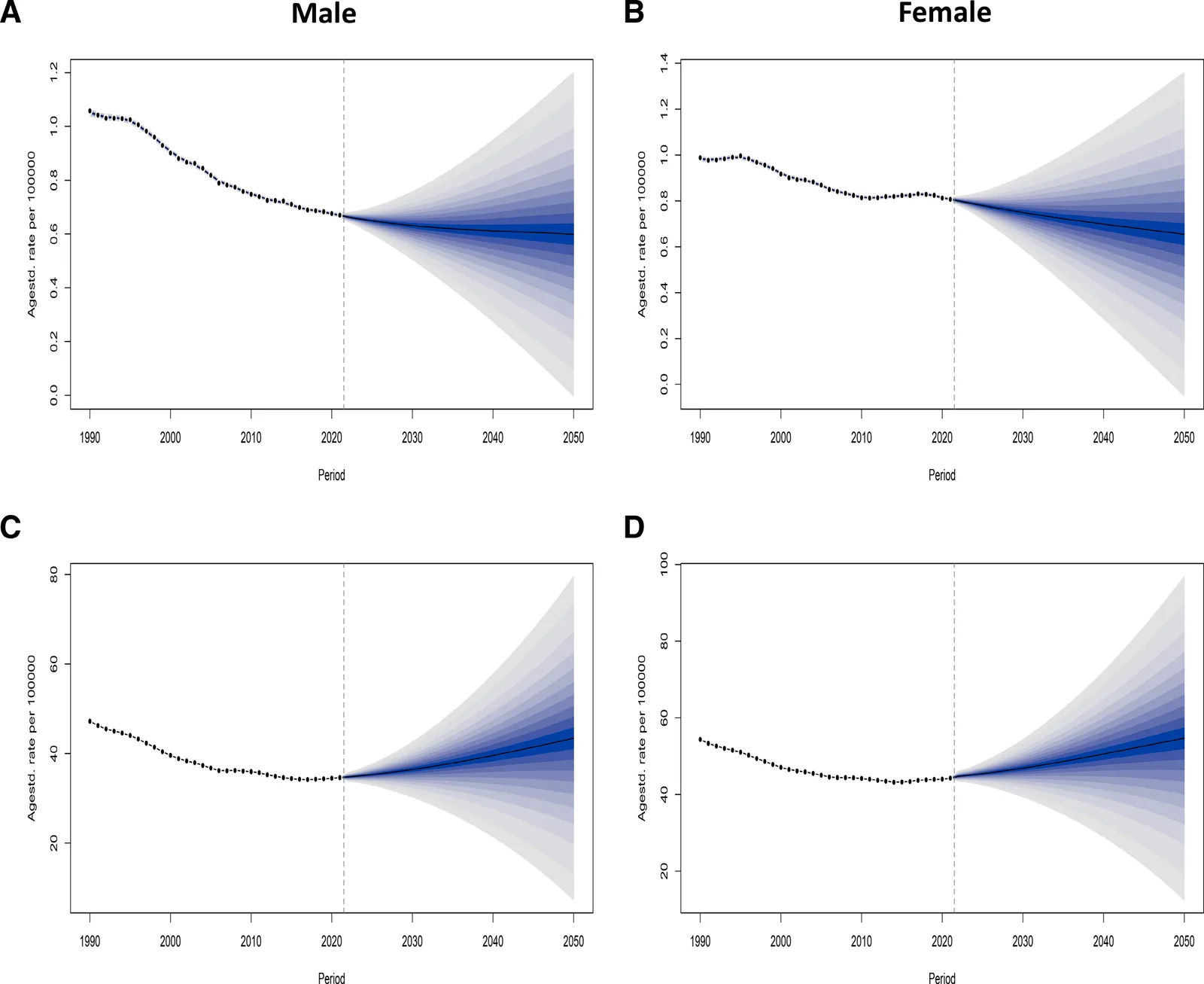
BAPC model-predicted age-standardized rates for high BMI-associated asthma by sex, 2022 to 2050. (A) ASMR (male). (B) ASMR (female). (C) ASDR (male). (D) ASDR (female). ASDR = age-standardized disability-adjusted life years rate, ASMR = age-standardized mortality rate, BAPC = Bayesian age–period–cohort, BMI = body mass index.

### 3.9. Bidirectional MR of the association between high BMI and asthma

Results from the MR suggested a positive, genetically supported association between BMI and asthma risk. The primary IVW analysis showed that each 1-unit increase in genetically predicted BMI was associated with higher odds of asthma (OR = 1.389, 95% CI: 1.303–1.479, *P* = 3.3 × 10^−24^; Fig. 9; Table 3). Using 361 genetic instruments, we observed substantial heterogeneity (Cochran’s *Q* = 669.07, *P* = 7.4 × 10^−21^; Table 3), which is consistent with widespread horizontal pleiotropy or other violations of the MR assumptions; the IVW estimate should therefore be interpreted with caution. The weighted median (OR = 1.282, 95% CI: 1.169–1.405, *P* = 1.2 × 10^−7^) and MR-Egger (OR = 1.317, 95% CI: 1.118–1.551, *P* = 1.1 × 10^−3^) estimates, which are more robust to horizontal pleiotropy than the IVW method, were directionally consistent with the IVW estimate (Table 3). The MR-PRESSO global test indicated the presence of pleiotropic outliers (*P* < 1 × 10^−4^). After removal of 3 outlier SNPs (rs1800437, rs429358, and rs6559365), the corrected estimate remained essentially unchanged (OR = 1.388, 95% CI: 1.305–1.476, *P* = 1.6 × 10^−22^; Table 3; Fig. S3, Supplemental Digital Content 7), and the distortion test showed no significant difference between the estimates before and after correction (*P* = .989; Table 3). Leave-one-out analysis confirmed that the association was not driven by any single SNP (Fig. S4, Supplemental Digital Content 8), and the funnel plot did not suggest pronounced asymmetry (Fig. S5, Supplemental Digital Content 9). The forest plot of individual SNP effects showed predominantly positive effect directions across variants (252/361, 69.8%), although most individual estimates had confidence intervals crossing the null, consistent with weak instrument strength (Fig. S6, Supplemental Digital Content 10). The MR-Egger intercept test did not indicate directional pleiotropy (*P* = .492; Table 3); however, this test has limited statistical power, particularly in the presence of substantial heterogeneity, and residual pleiotropy cannot be excluded. Therefore, the consistency of estimates across methods should be interpreted as supportive of, but not definitive for, a robust association.

**Table 3:** Mendelian randomization estimates for the associations of genetically predicted BMI with asthma and of asthma with BMI.

Method	n SNPs	OR	95% CI	*P* value
Forward MR (BMI → asthma)				
IVW (random effects)	361	1.389	1.303–1.479	3.3 × 10^−24^
Weighted median	361	1.282	1.169–1.405	1.2 × 10^−7^
MR-Egger	361	1.317	1.118–1.551	1.1 × 10^−3^
Simple mode	361	1.236	0.994–1.537	.058
Weighted mode	361	1.265	1.118–1.432	2.3 × 10^−4^
MR-Egger intercept	–	–	–	.492
Cochran’s *Q* test (IVW; *Q* = 669.07)	361	–	–	7.4 × 10^−21^
MR-PRESSO global test	361	–	–	<1 × 10^−4^
MR-PRESSO (raw)	361	1.389	1.303–1.479	3.3 × 10^−24^
MR-PRESSO (outlier-corrected)	358	1.388	1.305–1.476	1.6 × 10^−22^
MR-PRESSO distortion test	–	–	–	.989
Reverse MR (asthma → BMI)				
IVW	48	0.988	0.965–1.011	.296
Weighted median	48	0.984	0.968–1.000	.049
MR-Egger	48	0.988	0.922–1.058	.726
Simple mode	48	0.985	0.957–1.015	.331
Weighted mode	48	0.984	0.964–1.005	.141
MR-Egger intercept	–	–	–	.997

BMI = body mass index, CI = confidence interval, IVW = inverse-variance weighted, MR = Mendelian randomization, MR-PRESSO = MR-pleiotropy residual sum and outlier, OR = odds ratio, SNP = single-nucleotide polymorphism.

**Figure 9.**
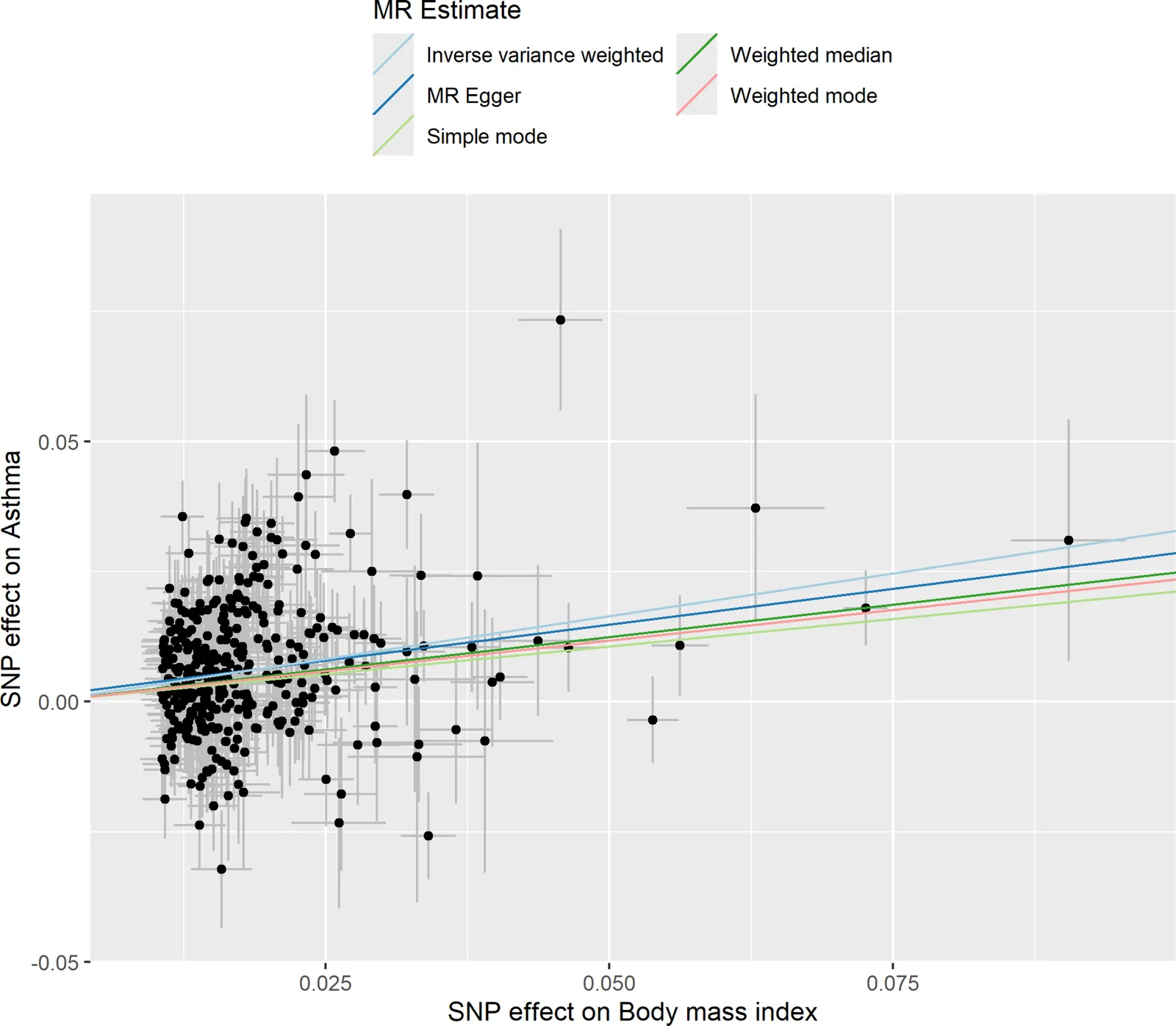
Scatter plot of SNP effects on BMI and asthma risk in the MR analysis of genetically predicted BMI and asthma. BMI = body mass index, MR = Mendelian randomization, SNP = single-nucleotide polymorphism.

To evaluate reverse causation, we performed a reverse MR analysis using 48 independent genetic instruments for asthma. The IVW analysis found no significant effect of genetically predicted asthma on BMI (OR = 0.988, 95% CI: 0.965–1.011, *P* = .296; Fig. S7, Supplemental Digital Content 11; Table 3). This null association was broadly consistent across all sensitivity analyses, with no evidence of directional pleiotropy (MR-Egger intercept *P* = .997; Table 3).

Collectively, these findings support a unidirectional, genetically supported association between higher BMI and asthma risk, with no evidence of a reverse association.

## 4. Discussion

This study integrates data from the GBD and GWAS to provide a comprehensive assessment of the burden of high BMI-related asthma and of the genetically supported association between high BMI and asthma risk. Key findings reveal a substantial growth in the absolute asthma burden attributable to high BMI over the 3 decades between 1990 and 2021. In contrast, ASMR and ASDR exhibited a downward trend. This pattern, indicative of “falling rates but rising numbers” underscores that population growth and aging, rather than increasing individual-level risk, are the main drivers of the growing absolute burden. These findings highlight an escalating public health challenge, necessitating a deeper exploration of the biological mechanisms and historical context of asthma management.

Epidemiological and clinical research has demonstrated a significant association between elevated BMI and the risk of asthma.^[7,22]^ Our study strengthens this evidence by integrating population-level burden estimates with genetic association evidence from MR. This approach not only corroborates the association but also quantifies its population-wide impact over 3 decades, extending previous research.^[23]^ These robust findings underscore the importance of weight management as a cornerstone of asthma clinical care and prevention, particularly for high-risk individuals.^[24]^ Beyond this modifiable risk factor, advancements in asthma treatment have also played a crucial role. The pronounced decline in ASMR and ASDR between 1996 and 2000 coincides with a pivotal shift in global asthma management, following the dissemination and implementation of the 1995 global initiative for asthma guidelines, which established inhaled corticosteroids as 1st-line anti-inflammatory therapy.^[25]^ This temporal association suggests that the standardization of care globally contributed to reducing asthma-related mortality and disability, a benefit supported by extensive evidence for inhaled corticosteroids in preventing exacerbations, hospitalizations, and death.^[26,27]^

Our analysis revealed substantial geographic heterogeneity in the asthma burden attributable to high BMI. India accounted for the greatest total count of deaths and DALYs, likely reflecting its large population size and the rapidly rising prevalence of obesity.^[28]^ In contrast, Pacific Island nations, notably Fiji and Kiribati, bore the heaviest burden in terms of ASRs, despite their smaller populations. This disparity may be influenced by genetic susceptibility,^[29]^ dietary transitions towards Western patterns, and declining physical activity associated with urbanization.^[30]^ Meanwhile, Zimbabwe experienced the most rapid increase in disease burden, a trend characteristic of the “dual burden of disease” often seen during socioeconomic transition.

A significant inverse association was found between the SDI and ASRs of high BMI-related asthma. Consistent with prior studies,^[9]^ high-SDI regions observed a consistent decline in both ASMR and ASDR. Conversely, the low-middle SDI quintile was the only group to experience an increase in ASRs from 1990 to 2021. Decomposition analysis revealed that in these regions, population growth and aging, compounded by unfavorable epidemiological changes, jointly drove the rising burden. This disparity likely reflects challenges unique to low-middle SDI settings, such as rapidly escalating obesity rates,^[31]^ deteriorating air quality,^[32]^ and constrained access to essential medicines, including a critical shortage of inhaled corticosteroids for asthma management.^[33]^ Addressing these inequities requires multisectoral strategies aimed at reducing dietary salt and sugar consumption, enforcing stricter air quality standards, and strengthening primary healthcare systems to enhance the coverage and quality of asthma diagnosis and treatment.

The asthma burden associated with high BMI also demonstrated distinct variations by age and sex, with the most pronounced impact observed among older adult females – a finding consistent with previous reports.^[9]^ This disparity may be explained by the interplay of hormonal fluctuations, body fat distribution, and inflammatory pathways. For example, the postmenopausal decline in estrogen promotes central adiposity,^[34]^ which can elevate levels of pro-inflammatory cytokines and leptin^[35]^ and impose mechanical loads on lung function.^[36]^ Furthermore, comorbidities such as cardiovascular disease and depression are highly prevalent in older women with obesity^[37,38]^ and can exacerbate asthma-related disability. These findings underscore the value of a life-course perspective. Implementing comprehensive strategies that integrate weight management, physical rehabilitation, and psychosocial support is essential to alleviate the disease burden in this vulnerable demographic.

The COVID-19 pandemic provided a unique context in which to assess the impact of high BMI on asthma burden. Although the GBD 2021 data encompass the initial years of the pandemic (2019–2021), our trend analysis did not reveal a significant deviation in the high BMI-related asthma burden trajectory during this period. This observation might be explained by countervailing mechanisms. On 1 hand, lockdowns and restrictions likely reduced physical activity and promoted unhealthy dietary shifts, contributing to rising obesity rates globally,^[39]^ a trend that could potentially increase asthma incidence. On the other hand, public health measures such as vaccination, mask-wearing, and remote work may have reduced exposure to respiratory viruses and other environmental triggers, thereby lowering the rate of acute asthma exacerbations.

Our projections suggest divergent future pathways: a continuing decline in ASMR but a slight rise in ASDR. This pattern of decreasing mortality alongside increasing disability likely reflects improvements in acute care and survival, yet underscores persistent chronic morbidity and health loss among people with high BMI.^[40]^ Consequently, global asthma management strategies should evolve beyond a primary focus on survival to also prioritize the improvement of long-term functional status and quality of life through sustained behavioral and clinical support.

Several limitations of this study warrant consideration. First, as our analysis relies on the GBD database, potential under-ascertainment of cases in low-income regions may lead to an underestimation of the true disease burden in these settings. Second, the MR analysis is subject to substantial heterogeneity across genetic variants (Cochran’s *Q* = 669.07, *P* = 7.4 × 10^−21^) and to pleiotropic outliers identified by MR-PRESSO, both of which violate the exclusion restriction assumption. Although multiple sensitivity analyses were performed, the IVW estimate may be biased by horizontal pleiotropy, and the InSIDE assumption underlying MR-Egger regression cannot be tested. The MR findings should therefore be interpreted as genetically supported associations rather than causal effects. Third, all GWAS summary statistics used in the MR analysis were derived from populations of European ancestry, whereas the settings that bear the highest absolute burden of high-BMI-attributable asthma, such as India and China, are predominantly non-European. Differences in LD structure, allele frequencies, and gene–environment interactions may therefore limit the transportability of our estimates. The genetically supported association reported here should be extrapolated to non-European populations with caution, and replication using ancestry-diverse GWAS is warranted. Fourth, whereas the forward MR analysis employed strong genetic instruments for BMI, the reverse MR analysis was constrained by a relatively limited number of independent SNPs for asthma. Although these instruments were robust (high *F* statistics), the smaller set may affect the power and robustness of sensitivity analyses. Fifth, despite the use of MR to minimize confounding, residual bias from unmeasured environmental factors (e.g., air pollution and smoking^[41,42]^) cannot be fully ruled out. Sixth, the BAPC projections extrapolate historical mortality and DALY trends, do not incorporate BMI as a time-varying covariate, and should therefore be interpreted as trend-based scenarios conditional on the continuation of past patterns. In regions undergoing rapid nutritional and obesity transitions – particularly low- and middle-SDI settings where obesity prevalence is rising fastest – the projections may underestimate future asthma burden attributable to high BMI. Finally, BMI is an imperfect proxy for adiposity, as it does not differentiate between fat and lean mass, potentially obscuring variations in obesity-related risk.

## 5. Conclusions

By integrating the GBD framework with MR, this study identifies high BMI as a risk factor genetically associated with the global asthma burden. The increasing absolute burden, driven largely by demographic forces, masks improvements in ASRs and exhibits substantial inequality across regions, SDI levels, age groups, and sexes. Moving forward, public health efforts must prioritize population-level obesity prevention, mitigate socioeconomic barriers to healthcare, and deliver tailored interventions that account for demographic and sex-specific disparities.

## Acknowledgments

We acknowledge the immense efforts of the Global Burden of Disease Study 2021 collaborators and the broader GBD network in producing and maintaining this vital public resource. We also thank the participants and investigators of the genome-wide association studies from which we obtained summary statistics.

## Author contributions

**Conceptualization:** Xiaokun Yao, Chuanli Zhang.

**Data curation:** Xiaokun Yao.

**Formal analysis:** Xiaokun Yao.

**Investigation:** Chuanli Zhang.

**Methodology:** Xiaokun Yao, Chuanli Zhang.

**Project administration:** Chuanli Zhang.

**Resources:** Chuanli Zhang.

**Supervision:** Chuanli Zhang.

**Visualization:** Xiaokun Yao.

**Writing – original draft:** Xiaokun Yao.

**Writing – review & editing:** Chuanli Zhang.























## References

[1] Venkatesan P. 2025 GINA report for asthma. Lancet Respir Med. 2025;13:e41–2. 10.1016/S2213-2600(25)00242-5 40582369

[2] GBD 2021 Asthma and Allergic Diseases Collaborators. Global, regional, and national burden of asthma and atopic dermatitis, 1990–2021, and projections to 2050: a systematic analysis of the Global Burden of Disease Study 2021. Lancet Respir Med. 2025;13:425–46. 10.1016/S2213-2600(25)00003-7 40147466

[3] Pividori M Schoettler N Nicolae DL Ober C Im HK. Shared and distinct genetic risk factors for childhood-onset and adult-onset asthma: genome-wide and transcriptome-wide studies. Lancet Respir Med. 2019;7:509–22. 10.1016/S2213-2600(19)30055-4 PMC6534440 31036433

[4] Byrwa-Hill BM Morphew TL Presto AA Fabisiak JP Wenzel SE. Living in environmental justice areas worsens asthma severity and control: differential interactions with disease duration, age at onset, and pollution. J Allergy Clin Immunol. 2023;152:1321–9.e5. 10.1016/j.jaci.2023.04.015 PMC10626048 37156327

[5] Seumois G Ramírez-Suástegui C Schmiedel BJ. Single-cell transcriptomic analysis of allergen-specific T cells in allergy and asthma. Sci Immunol. 2020;5:eaba6087. 10.1126/sciimmunol.aba6087 PMC7372639 32532832

[6] Yuan L Tao J Wang J. Global, regional, national burden of asthma from 1990 to 2021, with projections of incidence to 2050: a systematic analysis of the global burden of disease study 2021. EClinicalMedicine. 2025;80:103051. 10.1016/j.eclinm.2024.103051 PMC11764843 39867965

[7] Parasuaraman G Ayyasamy L Aune D. The association between body mass index, abdominal fatness, and weight change and the risk of adult asthma: a systematic review and meta-analysis of cohort studies. Sci Rep. 2023;13:7745. 10.1038/s41598-023-31373-6 PMC10182066 37173338

[8] Fang C Jiang Z Su X Fan W. The association between body mass index and asthma in children: a crosssectional study from NHANES 1999 to 2020. Sci Rep. 2025;15:9448. 10.1038/s41598-025-92619-z PMC11923108 40108226

[9] Tooba R Wu TD. Obesity and asthma: a focused review. Respir Med. 2022;204:107012. 10.1016/j.rmed.2022.107012 PMC9671155 36279813

[10] Wang Z Liu Y Li Y Wang Q Liu J. Global burden of asthma attributable to high body mass index in older adults 1990–2021 and prediction to 2050: an analysis of global burden of disease study 2021. World Allergy Organ J. 2025;18:101040. 10.1016/j.waojou.2025.101040 PMC11946875 40151545

[11] Liu J Yuan M Chen Y. Global burden of asthma associated with high body mass index from 1990 to 2019. Ann Allergy Asthma Immunol. 2022;129:720–30.e8. 10.1016/j.anai.2022.08.013 36002091

[12] GBD 2021 Risk Factors Collaborators. Global burden and strength of evidence for 88 risk factors in 204 countries and 811 subnational locations, 1990–2021: a systematic analysis for the global burden of disease study 2021. Lancet. 2024;403:2162–203. 10.1016/S0140-6736(24)00933-4 PMC11120204 38762324

[13] GBD 2021 Diseases and Injuries Collaborators. Global incidence, prevalence, years lived with disability (YLDs), disability-adjusted life-years (DALYs), and healthy life expectancy (HALE) for 371 diseases and injuries in 204 countries and territories and 811 subnational locations, 1990–2021: a systematic analysis for the global burden of disease study 2021. Lancet. 2024;403:2133–61. 10.1016/S0140-6736(24)00757-8 PMC11122111 38642570

[14] Barton AR Sherman MA Mukamel RE Loh PR. Whole-exome imputation within UK Biobank powers rare coding variant association and fine-mapping analyses. Nat Genet. 2021;53:1260–9. 10.1038/s41588-021-00892-1 PMC8349845 34226706

[15] Sakaue S Kanai M Tanigawa Y; FinnGen. A cross-population atlas of genetic associations for 220 human phenotypes. Nat Genet. 2021;53:1415–24. 10.1038/s41588-021-00931-x PMC12208603 34594039

[16] Wang Z Li Y Gao Y. Global, regional, and national burden of asthma and its attributable risk factors from 1990 to 2019: a systematic analysis for the global burden of disease study 2019. Respir Res. 2023;24:169. 10.1186/s12931-023-02475-6 PMC10288698 37353829

[17] Zhou XD Chen QF Yang W. Burden of disease attributable to high body mass index: an analysis of data from the global burden of disease study 2021. EClinicalMedicine. 2024;76:102848. 10.1016/j.eclinm.2024.102848 PMC11462227 39386160

[18] Das Gupta P. A general method of decomposing a difference between two rates into several components. Demography. 1978;15:99–112. 631402

[19] Riebler A Held L. Projecting the future burden of cancer: Bayesian age-period-cohort analysis with integrated nested Laplace approximations. Biom J. 2017;59:531–49. 10.1002/bimj.201500263 28139001

[20] Li J Wei J Wang J. Association between gut microbiota and spinal stenosis: a two-sample Mendelian randomization study. Front Immunol. 2024;15:1360132. 10.3389/fimmu.2024.1360132 PMC11066289 38707908

[21] Skrivankova VW Richmond RC Woolf BAR. Strengthening the reporting of observational studies in epidemiology using Mendelian randomisation (STROBE-MR): explanation and elaboration. BMJ. 2021;375:n2233. 10.1136/bmj.n2233 PMC8546498 34702754

[22] Bal C Pohl W Milger K. Characterization of obesity in severe asthma in the German asthma net. J Allergy Clin Immunol Pract. 2023;11:3417–24.e3. 10.1016/j.jaip.2023.06.049 37406803

[23] Huang J Zhou X Dong B. Obesity-related asthma and its relationship with microbiota. Front Cell Infect Microbiol. 2023;13:1303899. 10.3389/fcimb.2023.1303899 PMC10825962 38292857

[24] Reyes-Angel J Kaviany P Rastogi D Forno E. Obesity-related asthma in children and adolescents. Lancet Child Adolesc Health. 2022;6:713–24. 10.1016/S2352-4642(22)00185-7 PMC12090466 35988550

[25] Asthma GIf. Global Strategy for Asthma Management and Prevention. National Institutes of Health, National Heart, Lung, and Blood Institute;1995.

[26] Kips JC Pauwels RA. Low dose inhaled corticosteroids and the prevention of death from asthma. Thorax. 2001;56(Suppl 2): ii74–8. PMC1765979 11514710

[27] Suissa S Ernst P Kezouh A. Regular use of inhaled corticosteroids and the long term prevention of hospitalisation for asthma. Thorax. 2002;57:880–4. 10.1136/thorax.57.10.880 PMC1746208 12324675

[28] Tham KW Ghani RA Cua SC. Obesity in South and Southeast Asia – a new consensus on care and management. Obes Rev. 2023;24:e13520. 10.1111/obr.13520 PMC10078503 36453081

[29] Sun H Lin M Russell EM. The impact of global and local Polynesian genetic ancestry on complex traits in Native Hawaiians. PLoS Genet. 2021;17:e1009273. 10.1371/journal.pgen.1009273 PMC7877570 33571193

[30] Statovci D Aguilera M MacSharry J Melgar S. The impact of western diet and nutrients on the microbiota and immune response at mucosal interfaces. Front Immunol. 2017;8:838. 10.3389/fimmu.2017.00838 PMC5532387 28804483

[31] GBD 2021 Adult BMI Collaborators. Global, regional, and national prevalence of adult overweight and obesity, 1990–2021, with forecasts to 2050: a forecasting study for the global burden of disease study 2021. Lancet. 2025;405:813–38. 10.1016/S0140-6736(25)00355-1 PMC11920007 40049186

[32] Fang T Di Y Xu Y. Temporal trends of particulate matter pollution and its health burden, 1990–2021, with projections to 2036: a systematic analysis for the global burden of disease study 2021. Front Public Health. 2025;13:1579716. 10.3389/fpubh.2025.1579716 PMC12041061 40308905

[33] Mortimer K Reddel HK Pitrez PM Bateman ED. Asthma management in low and middle income countries: case for change. Eur Respir J. 2022;60:2103179. 10.1183/13993003.03179-2021 PMC9474897 35210321

[34] Juppi HK Sipilä S Fachada V. Total and regional body adiposity increases during menopause-evidence from a follow-up study. Aging Cell. 2022;21:e13621. 10.1111/acel.13621 PMC9197413 35509177

[35] Sánchez-Ortega H Jiménez-Cortegana C Novalbos-Ruiz JP Gómez-Bastero A Soto-Campos JG Sánchez-Margalet V. Role of leptin as a link between asthma and obesity: a systematic review and meta-analysis. Int J Mol Sci. 2022;24:546. 10.3390/ijms24010546 PMC9820321 36613991

[36] Miethe S Karsonova A Karaulov A Renz H. Obesity and asthma. J Allergy Clin Immunol. 2020;146:685–93. 10.1016/j.jaci.2020.08.011 33032723

[37] Shan J Yin R Panuthai S. Body mass index and multimorbidity risk: a systematic review and dose-response meta-analysis. Arch Gerontol Geriatr. 2024;123:105418. 10.1016/j.archger.2024.105418 38604087

[38] Cazzola M Rogliani P Ora J Calzetta L Matera MG. Asthma and comorbidities: recent advances. Pol Arch Intern Med. 2022;132:16250. 10.20452/pamw.16250 35485651

[39] Nour TY Altintaş KH. Effect of the COVID-19 pandemic on obesity and it is risk factors: a systematic review. BMC Public Health. 2023;23:1018. 10.1186/s12889-023-15833-2 PMC10227822 37254139

[40] Born CDC Bhadra R D’Souza G. Combined lifestyle interventions in the prevention and management of asthma and COPD: a systematic review. Nutrients. 2024;16:1515. 10.3390/nu16101515 PMC11124109 38794757

[41] Luo C Wei T Jiang W. The association between air pollution and obesity: an umbrella review of meta-analyses and systematic reviews. BMC Public Health. 2024;24:1856. 10.1186/s12889-024-19370-4 PMC11238414 38992628

[42] Carrasquilla GD García-Ureña M Romero-Lado MJ Kilpeläinen TO. Estimating causality between smoking and abdominal obesity by Mendelian randomization. Addiction. 2024;119:1024–34. 10.1111/add.16454 38509034

