# Supplementary material for: The influence of high body mass index on asthma: A study integrating GBD 2021 and Mendelian randomization
Source: Medicine (Baltimore). 2026 Nov 6;105(45):e51490. doi: 10.1097/MD.0000000000051490 (PMC13747491; doi:10.1097/MD.0000000000051490)
Supplement: Supplementary file 1 [file medi-105-e51490-s001.docx]

**Table S1.** Characteristics of the 361 genetic instruments used in the Mendelian randomization analysis (exposure: BMI; outcome: asthma)

| **SNP** | **Effect/Other allele (BMI)** | **EAF** | **β (BMI)** | **SE (BMI)** | **P (BMI)** | **N (BMI)** | **β (asthma)** | **SE (asthma)** | **P (asthma)** | **F-statistic** |
| --- | --- | --- | --- | --- | --- | --- | --- | --- | --- | --- |
| rs10005135 | A>C | 0.305 | 0.0183 | 0.0021 | 1.30e-18 | 457756 | 0.0072 | 0.0073 | 3.23e-01 | 77.2 |
| rs10146997 | G>A | 0.222 | 0.0275 | 0.0023 | 2.80e-33 | 457756 | 0.0128 | 0.0095 | 1.80e-01 | 141.4 |
| rs10172678 | C>T | 0.604 | -0.0196 | 0.0019 | 6.80e-24 | 457756 | -0.0151 | 0.0072 | 3.54e-02 | 100.8 |
| rs10207878 | G>A | 0.437 | -0.0141 | 0.0019 | 8.20e-13 | 457756 | 0.0146 | 0.0077 | 5.81e-02 | 54.1 |
| rs10209821 | T>C | 0.343 | 0.0172 | 0.0020 | 1.70e-17 | 457756 | 0.0141 | 0.0072 | 5.17e-02 | 73.1 |
| rs1029969 | A>G | 0.735 | 0.0128 | 0.0022 | 1.60e-08 | 457756 | 0.0007 | 0.0076 | 9.28e-01 | 34.6 |
| rs1030221 | T>C | 0.479 | 0.0140 | 0.0019 | 2.00e-13 | 457756 | -0.0039 | 0.0073 | 5.93e-01 | 53.6 |
| rs1048932 | A>C | 0.412 | -0.0157 | 0.0020 | 9.80e-16 | 457756 | -0.0017 | 0.0070 | 8.03e-01 | 63.7 |
| rs10494720 | G>A | 0.377 | -0.0141 | 0.0020 | 3.00e-13 | 457756 | -0.0022 | 0.0074 | 7.65e-01 | 51.5 |
| rs10507483 | C>T | 0.166 | 0.0164 | 0.0026 | 1.10e-10 | 457756 | 0.0136 | 0.0104 | 1.90e-01 | 40.5 |
| rs10510419 | T>G | 0.147 | -0.0158 | 0.0027 | 1.50e-09 | 457756 | 0.0322 | 0.0113 | 4.41e-03 | 34.4 |
| rs1061801 | A>G | 0.191 | 0.0177 | 0.0025 | 1.40e-12 | 457756 | 0.0298 | 0.0098 | 2.33e-03 | 48.6 |
| rs1064213 | A>G | 0.477 | 0.0141 | 0.0019 | 1.30e-13 | 457756 | -0.0023 | 0.0071 | 7.47e-01 | 54.4 |
| rs10738406 | A>G | 0.446 | -0.0191 | 0.0019 | 5.30e-23 | 457756 | -0.0121 | 0.0073 | 9.78e-02 | 97.3 |
| rs10756801 | T>G | 0.643 | -0.0148 | 0.0020 | 3.70e-13 | 457756 | 0.0029 | 0.0084 | 7.34e-01 | 54.1 |
| rs10788918 | G>T | 0.682 | -0.0182 | 0.0020 | 1.80e-19 | 457756 | -0.0228 | 0.0073 | 1.68e-03 | 79.0 |
| rs10795422 | G>A | 0.689 | 0.0144 | 0.0021 | 4.60e-12 | 457756 | 0.0008 | 0.0081 | 9.20e-01 | 48.0 |
| rs10818810 | A>G | 0.383 | 0.0129 | 0.0020 | 2.40e-11 | 457756 | -0.0071 | 0.0071 | 3.18e-01 | 42.4 |
| rs10819759 | G>A | 0.314 | 0.0168 | 0.0021 | 4.60e-16 | 457756 | 0.0304 | 0.0081 | 1.81e-04 | 65.6 |
| rs1083455 | A>G | 0.579 | -0.0166 | 0.0019 | 4.50e-18 | 457756 | -0.0198 | 0.0074 | 7.12e-03 | 73.5 |
| rs10851975 | A>C | 0.849 | 0.0149 | 0.0027 | 1.50e-08 | 457756 | 0.0044 | 0.0114 | 6.98e-01 | 30.5 |
| rs10894294 | A>C | 0.512 | 0.0145 | 0.0019 | 3.50e-14 | 457756 | 0.0060 | 0.0069 | 3.88e-01 | 57.3 |
| rs10914685 | T>A | 0.325 | -0.0120 | 0.0020 | 5.70e-09 | 457756 | 0.0046 | 0.0077 | 5.50e-01 | 34.8 |
| rs10938397 | G>A | 0.435 | 0.0294 | 0.0019 | 3.10e-52 | 457756 | 0.0027 | 0.0071 | 6.98e-01 | 230.6 |
| rs10953740 | G>A | 0.555 | -0.0161 | 0.0019 | 1.20e-16 | 457756 | -0.0151 | 0.0080 | 6.12e-02 | 70.0 |
| rs10968576 | G>A | 0.322 | 0.0240 | 0.0021 | 1.20e-31 | 457756 | 0.0025 | 0.0076 | 7.43e-01 | 137.0 |
| rs11000112 | C>T | 0.415 | 0.0122 | 0.0019 | 1.00e-10 | 457756 | -0.0053 | 0.0079 | 5.01e-01 | 39.0 |
| rs11000945 | A>G | 0.123 | 0.0189 | 0.0029 | 1.00e-10 | 457756 | 0.0258 | 0.0114 | 2.36e-02 | 42.0 |
| rs11086089 | T>C | 0.408 | 0.0160 | 0.0020 | 1.40e-16 | 457756 | 0.0180 | 0.0071 | 1.18e-02 | 67.1 |
| rs11097236 | A>G | 0.201 | 0.0134 | 0.0024 | 4.50e-08 | 457756 | 0.0108 | 0.0082 | 1.90e-01 | 31.2 |
| rs11165513 | T>C | 0.385 | 0.0131 | 0.0020 | 1.70e-11 | 457756 | -0.0029 | 0.0072 | 6.85e-01 | 44.5 |
| rs11189513 | G>A | 0.316 | -0.0180 | 0.0021 | 7.70e-18 | 457756 | -0.0015 | 0.0076 | 8.48e-01 | 75.2 |
| rs11201992 | A>C | 0.458 | -0.0123 | 0.0019 | 1.40e-10 | 457756 | -0.0134 | 0.0070 | 5.66e-02 | 40.4 |
| rs1125683 | G>A | 0.448 | 0.0117 | 0.0019 | 1.10e-09 | 457756 | -0.0112 | 0.0069 | 1.04e-01 | 36.6 |
| rs1126930 | C>G | 0.035 | 0.0329 | 0.0053 | 4.10e-10 | 457756 | 0.0043 | 0.0218 | 8.44e-01 | 38.4 |
| rs11374767 | AC>A | 0.390 | -0.0139 | 0.0020 | 1.50e-12 | 457756 | 0.0010 | 0.0074 | 8.91e-01 | 48.5 |
| rs114263339 | T>C | 0.026 | 0.0390 | 0.0060 | 7.20e-11 | 457756 | -0.0076 | 0.0253 | 7.63e-01 | 41.7 |
| rs11521066 | C>T | 0.289 | 0.0121 | 0.0021 | 1.70e-09 | 457756 | 0.0066 | 0.0077 | 3.95e-01 | 33.1 |
| rs11538 | G>A | 0.172 | 0.0149 | 0.0026 | 1.40e-09 | 457756 | 0.0061 | 0.0103 | 5.53e-01 | 33.6 |
| rs11542009 | T>C | 0.102 | 0.0226 | 0.0032 | 3.90e-13 | 457756 | 0.0394 | 0.0140 | 4.81e-03 | 51.0 |
| rs11575375 | A>G | 0.345 | 0.0121 | 0.0020 | 9.90e-10 | 457756 | 0.0151 | 0.0075 | 4.57e-02 | 36.0 |
| rs1159974 | C>T | 0.525 | 0.0129 | 0.0019 | 8.20e-12 | 457756 | 0.0062 | 0.0071 | 3.82e-01 | 45.3 |
| rs11622224 | T>C | 0.523 | 0.0111 | 0.0019 | 1.10e-08 | 457756 | 0.0071 | 0.0074 | 3.32e-01 | 33.4 |
| rs11630385 | G>A | 0.467 | 0.0112 | 0.0019 | 3.20e-09 | 457756 | -0.0024 | 0.0078 | 7.57e-01 | 33.4 |
| rs11668587 | G>A | 0.333 | -0.0177 | 0.0020 | 5.30e-18 | 457756 | -0.0013 | 0.0073 | 8.57e-01 | 75.8 |
| rs11679338 | T>C | 0.662 | 0.0171 | 0.0020 | 6.50e-18 | 457756 | 0.0013 | 0.0076 | 8.60e-01 | 72.3 |
| rs11694662 | T>C | 0.291 | 0.0116 | 0.0021 | 5.80e-09 | 457756 | -0.0037 | 0.0080 | 6.40e-01 | 30.7 |
| rs117176448 | G>C | 0.096 | 0.0225 | 0.0033 | 1.30e-11 | 457756 | 0.0254 | 0.0139 | 6.73e-02 | 47.7 |
| rs11729908 | T>C | 0.492 | -0.0107 | 0.0019 | 1.70e-08 | 457756 | -0.0093 | 0.0071 | 1.93e-01 | 31.3 |
| rs11739877 | T>C | 0.622 | 0.0120 | 0.0020 | 6.30e-10 | 457756 | 0.0103 | 0.0070 | 1.44e-01 | 36.7 |
| rs1177619 | C>T | 0.526 | -0.0139 | 0.0019 | 1.80e-13 | 457756 | 0.0163 | 0.0072 | 2.32e-02 | 52.6 |
| rs11776713 | C>T | 0.477 | -0.0123 | 0.0019 | 1.30e-10 | 457756 | 0.0008 | 0.0071 | 9.08e-01 | 40.8 |
| rs117829164 | G>T | 0.119 | -0.0233 | 0.0030 | 3.60e-15 | 457756 | -0.0300 | 0.0132 | 2.30e-02 | 61.3 |
| rs11806197 | G>A | 0.191 | 0.0245 | 0.0024 | 1.60e-23 | 457756 | 0.0161 | 0.0085 | 5.78e-02 | 102.1 |
| rs11864909 | T>C | 0.286 | 0.0115 | 0.0021 | 4.00e-08 | 457756 | -0.0070 | 0.0079 | 3.77e-01 | 29.2 |
| rs11954149 | G>A | 0.537 | -0.0115 | 0.0019 | 4.80e-09 | 457756 | 0.0058 | 0.0073 | 4.25e-01 | 35.6 |
| rs12140153 | T>G | 0.097 | -0.0365 | 0.0033 | 1.00e-28 | 457756 | 0.0054 | 0.0142 | 7.04e-01 | 123.7 |
| rs12152961 | A>G | 0.213 | 0.0130 | 0.0023 | 3.00e-08 | 457756 | 0.0009 | 0.0099 | 9.29e-01 | 30.9 |
| rs12209834 | A>C | 0.244 | -0.0129 | 0.0022 | 9.10e-09 | 457756 | 0.0052 | 0.0076 | 4.95e-01 | 33.3 |
| rs1229984 | C>T | 0.970 | 0.0437 | 0.0062 | 9.80e-14 | 457756 | 0.0117 | 0.0145 | 4.19e-01 | 50.5 |
| rs12325419 | A>G | 0.116 | -0.0224 | 0.0030 | 3.30e-14 | 457756 | 0.0002 | 0.0111 | 9.84e-01 | 56.1 |
| rs12331308 | A>G | 0.182 | -0.0150 | 0.0025 | 2.20e-09 | 457756 | 0.0013 | 0.0084 | 8.82e-01 | 36.6 |
| rs12344695 | G>A | 0.448 | -0.0127 | 0.0019 | 6.10e-11 | 457756 | 0.0066 | 0.0069 | 3.45e-01 | 43.7 |
| rs12364470 | G>T | 0.165 | 0.0199 | 0.0026 | 1.10e-14 | 457756 | -0.0018 | 0.0104 | 8.60e-01 | 59.0 |
| rs12435157 | A>G | 0.287 | -0.0151 | 0.0021 | 8.30e-13 | 457756 | -0.0188 | 0.0084 | 2.59e-02 | 50.5 |
| rs12459368 | G>A | 0.268 | -0.0177 | 0.0022 | 2.90e-16 | 457756 | -0.0106 | 0.0077 | 1.66e-01 | 66.2 |
| rs12500459 | A>G | 0.130 | 0.0181 | 0.0028 | 2.80e-10 | 457756 | 0.0169 | 0.0100 | 8.98e-02 | 40.2 |
| rs12514473 | C>T | 0.242 | -0.0173 | 0.0022 | 7.90e-15 | 457756 | 0.0047 | 0.0088 | 5.89e-01 | 59.8 |
| rs12543508 | T>C | 0.110 | 0.0217 | 0.0031 | 6.80e-12 | 457756 | 0.0044 | 0.0109 | 6.87e-01 | 49.9 |
| rs1260326 | C>T | 0.598 | 0.0110 | 0.0020 | 1.40e-08 | 457756 | 0.0015 | 0.0071 | 8.27e-01 | 31.4 |
| rs12639116 | T>C | 0.097 | -0.0179 | 0.0032 | 3.20e-08 | 457756 | -0.0175 | 0.0118 | 1.36e-01 | 30.2 |
| rs12764049 | G>A | 0.226 | 0.0169 | 0.0023 | 1.90e-13 | 457756 | -0.0090 | 0.0079 | 2.55e-01 | 54.4 |
| rs12881629 | G>A | 0.082 | 0.0212 | 0.0035 | 3.90e-10 | 457756 | -0.0038 | 0.0148 | 7.97e-01 | 36.7 |
| rs12921986 | G>A | 0.078 | 0.0207 | 0.0036 | 4.40e-09 | 457756 | 0.0311 | 0.0158 | 4.95e-02 | 33.3 |
| rs13006913 | A>G | 0.704 | 0.0117 | 0.0021 | 2.50e-08 | 457756 | 0.0189 | 0.0083 | 2.25e-02 | 31.6 |
| rs13038765 | A>G | 0.101 | -0.0178 | 0.0032 | 2.70e-08 | 457756 | 0.0175 | 0.0148 | 2.35e-01 | 30.8 |
| rs13054 | C>T | 0.592 | -0.0130 | 0.0020 | 8.30e-11 | 457756 | -0.0031 | 0.0071 | 6.64e-01 | 43.9 |
| rs13107325 | T>C | 0.075 | 0.0457 | 0.0037 | 4.80e-37 | 457756 | 0.0734 | 0.0174 | 2.50e-05 | 152.8 |
| rs13201510 | A>G | 0.210 | -0.0146 | 0.0024 | 8.70e-10 | 457756 | -0.0078 | 0.0112 | 4.89e-01 | 38.7 |
| rs13233916 | G>C | 0.089 | -0.0235 | 0.0034 | 4.50e-12 | 457756 | 0.0055 | 0.0096 | 5.68e-01 | 48.9 |
| rs13243284 | C>A | 0.421 | 0.0179 | 0.0020 | 9.20e-21 | 457756 | -0.0097 | 0.0070 | 1.67e-01 | 83.6 |
| rs13417131 | C>T | 0.109 | 0.0174 | 0.0031 | 2.60e-08 | 457756 | 0.0200 | 0.0131 | 1.27e-01 | 32.1 |
| rs1364063 | C>T | 0.409 | -0.0226 | 0.0020 | 2.60e-32 | 457756 | -0.0105 | 0.0075 | 1.61e-01 | 133.8 |
| rs13642 | T>A | 0.360 | -0.0173 | 0.0020 | 2.60e-18 | 457756 | -0.0171 | 0.0072 | 1.86e-02 | 74.5 |
| rs1388693 | A>G | 0.757 | 0.0141 | 0.0022 | 5.50e-10 | 457756 | 0.0086 | 0.0077 | 2.66e-01 | 40.0 |
| rs1406779 | T>C | 0.320 | 0.0223 | 0.0020 | 8.70e-28 | 457756 | -0.0038 | 0.0081 | 6.43e-01 | 118.5 |
| rs1412971 | T>G | 0.563 | -0.0148 | 0.0019 | 1.30e-14 | 457756 | -0.0089 | 0.0070 | 2.04e-01 | 59.3 |
| rs1426172 | G>A | 0.318 | -0.0133 | 0.0021 | 2.10e-11 | 457756 | -0.0016 | 0.0072 | 8.27e-01 | 41.8 |
| rs144443274 | T>C | 0.175 | 0.0208 | 0.0025 | 2.10e-16 | 457756 | 0.0051 | 0.0106 | 6.28e-01 | 66.8 |
| rs1462433 | A>G | 0.298 | -0.0221 | 0.0021 | 2.00e-25 | 457756 | -0.0082 | 0.0074 | 2.69e-01 | 110.3 |
| rs1484375 | G>A | 0.779 | -0.0129 | 0.0023 | 9.10e-09 | 457756 | -0.0062 | 0.0079 | 4.36e-01 | 31.1 |
| rs1491905 | C>T | 0.505 | -0.0167 | 0.0019 | 2.50e-18 | 457756 | -0.0180 | 0.0071 | 1.09e-02 | 75.7 |
| rs1578407 | T>C | 0.729 | 0.0126 | 0.0022 | 1.00e-08 | 457756 | -0.0048 | 0.0088 | 5.86e-01 | 33.7 |
| rs1579557 | T>C | 0.296 | 0.0230 | 0.0021 | 1.40e-27 | 457756 | 0.0090 | 0.0075 | 2.31e-01 | 120.3 |
| rs159037 | C>T | 0.253 | 0.0132 | 0.0022 | 4.10e-09 | 457756 | 0.0138 | 0.0082 | 9.26e-02 | 35.7 |
| rs1657930 | A>G | 0.804 | -0.0147 | 0.0024 | 6.20e-10 | 457756 | -0.0235 | 0.0086 | 6.30e-03 | 36.8 |
| rs16826093 | A>T | 0.210 | 0.0179 | 0.0024 | 4.00e-15 | 457756 | 0.0345 | 0.0087 | 7.12e-05 | 57.9 |
| rs16904538 | A>G | 0.116 | 0.0169 | 0.0030 | 1.40e-08 | 457756 | 0.0034 | 0.0103 | 7.40e-01 | 31.8 |
| rs17040743 | C>A | 0.374 | 0.0116 | 0.0020 | 1.30e-09 | 457756 | 0.0083 | 0.0077 | 2.82e-01 | 34.1 |
| rs17071756 | C>T | 0.169 | 0.0149 | 0.0026 | 2.20e-08 | 457756 | -0.0049 | 0.0093 | 5.96e-01 | 32.6 |
| rs17425707 | C>T | 0.104 | 0.0186 | 0.0031 | 1.50e-08 | 457756 | 0.0281 | 0.0138 | 4.16e-02 | 35.3 |
| rs17782313 | C>T | 0.233 | 0.0538 | 0.0023 | 3.90e-125 | 457756 | -0.0035 | 0.0083 | 6.72e-01 | 560.8 |
| rs1784302 | G>C | 0.394 | 0.0131 | 0.0020 | 4.00e-10 | 457756 | 0.0037 | 0.0077 | 6.31e-01 | 40.8 |
| rs179442 | T>C | 0.338 | -0.0181 | 0.0020 | 1.70e-19 | 457756 | 0.0015 | 0.0072 | 8.30e-01 | 79.2 |
| rs1800437 | C>G | 0.194 | -0.0340 | 0.0024 | 4.90e-44 | 457756 | 0.0258 | 0.0084 | 2.04e-03 | 195.1 |
| rs1805123 | G>T | 0.243 | -0.0163 | 0.0022 | 3.80e-13 | 457756 | -0.0086 | 0.0106 | 4.14e-01 | 52.4 |
| rs1837680 | A>G | 0.221 | -0.0140 | 0.0023 | 1.70e-09 | 457756 | -0.0174 | 0.0087 | 4.67e-02 | 36.8 |
| rs1860561 | A>G | 0.227 | 0.0146 | 0.0023 | 1.50e-10 | 457756 | 0.0176 | 0.0094 | 6.01e-02 | 40.0 |
| rs1884630 | G>A | 0.192 | -0.0163 | 0.0024 | 5.30e-11 | 457756 | 0.0120 | 0.0083 | 1.51e-01 | 44.4 |
| rs1927788 | C>A | 0.465 | 0.0122 | 0.0019 | 1.00e-10 | 457756 | -0.0005 | 0.0070 | 9.41e-01 | 39.8 |
| rs1937462 | G>A | 0.593 | 0.0113 | 0.0019 | 4.10e-09 | 457756 | 0.0134 | 0.0071 | 5.91e-02 | 34.1 |
| rs1949203 | G>A | 0.764 | 0.0139 | 0.0023 | 1.00e-09 | 457756 | -0.0238 | 0.0086 | 5.81e-03 | 37.7 |
| rs1951716 | T>C | 0.631 | 0.0202 | 0.0020 | 1.40e-24 | 457756 | 0.0315 | 0.0075 | 2.45e-05 | 101.7 |
| rs1970801 | T>G | 0.594 | -0.0108 | 0.0020 | 3.80e-08 | 457756 | 0.0120 | 0.0070 | 8.54e-02 | 30.5 |
| rs197393 | C>T | 0.379 | 0.0140 | 0.0020 | 2.50e-13 | 457756 | 0.0019 | 0.0071 | 7.90e-01 | 50.6 |
| rs1973993 | C>T | 0.597 | 0.0195 | 0.0019 | 2.30e-23 | 457756 | 0.0165 | 0.0075 | 2.78e-02 | 100.3 |
| rs2015407 | G>A | 0.650 | -0.0181 | 0.0020 | 3.40e-20 | 457756 | 0.0025 | 0.0071 | 7.22e-01 | 80.9 |
| rs2017657 | T>G | 0.574 | -0.0192 | 0.0019 | 6.60e-23 | 457756 | -0.0238 | 0.0073 | 1.21e-03 | 97.3 |
| rs2072758 | T>C | 0.111 | 0.0218 | 0.0030 | 9.00e-13 | 457756 | -0.0059 | 0.0104 | 5.70e-01 | 51.7 |
| rs2074613 | T>C | 0.560 | 0.0123 | 0.0019 | 2.10e-10 | 457756 | 0.0175 | 0.0069 | 1.16e-02 | 40.6 |
| rs2076561 | A>G | 0.333 | 0.0150 | 0.0020 | 5.90e-13 | 457756 | -0.0094 | 0.0072 | 1.95e-01 | 53.5 |
| rs2076603 | A>G | 0.634 | -0.0128 | 0.0020 | 1.20e-10 | 457756 | -0.0051 | 0.0072 | 4.79e-01 | 40.2 |
| rs2110499 | G>A | 0.464 | 0.0143 | 0.0019 | 3.10e-14 | 457756 | 0.0004 | 0.0071 | 9.58e-01 | 55.3 |
| rs215614 | A>G | 0.628 | -0.0154 | 0.0020 | 1.10e-14 | 457756 | 0.0015 | 0.0073 | 8.40e-01 | 60.0 |
| rs2172131 | C>T | 0.579 | -0.0140 | 0.0019 | 8.80e-13 | 457756 | 0.0044 | 0.0078 | 5.70e-01 | 52.0 |
| rs217669 | C>T | 0.271 | 0.0177 | 0.0022 | 1.80e-15 | 457756 | 0.0095 | 0.0077 | 2.16e-01 | 67.1 |
| rs2188806 | T>C | 0.476 | 0.0117 | 0.0019 | 1.90e-09 | 457756 | 0.0117 | 0.0070 | 9.50e-02 | 37.3 |
| rs2228215 | G>A | 0.351 | -0.0139 | 0.0020 | 1.40e-11 | 457756 | -0.0188 | 0.0073 | 1.03e-02 | 47.3 |
| rs2228550 | G>T | 0.641 | 0.0129 | 0.0020 | 4.00e-10 | 457756 | 0.0285 | 0.0073 | 9.76e-05 | 40.3 |
| rs2231142 | T>G | 0.113 | -0.0201 | 0.0030 | 1.40e-11 | 457756 | -0.0043 | 0.0099 | 6.67e-01 | 43.7 |
| rs2234458 | T>C | 0.637 | -0.0188 | 0.0020 | 2.90e-21 | 457756 | 0.0051 | 0.0072 | 4.79e-01 | 88.5 |
| rs2250377 | G>A | 0.661 | -0.0236 | 0.0020 | 1.50e-31 | 457756 | -0.0132 | 0.0080 | 9.87e-02 | 134.4 |
| rs2271264 | A>G | 0.090 | 0.0248 | 0.0034 | 1.70e-13 | 457756 | 0.0123 | 0.0105 | 2.39e-01 | 54.7 |
| rs2271818 | G>C | 0.334 | -0.0120 | 0.0021 | 1.10e-08 | 457756 | 0.0013 | 0.0072 | 8.55e-01 | 33.4 |
| rs2273699 | G>A | 0.337 | -0.0153 | 0.0020 | 2.20e-14 | 457756 | -0.0058 | 0.0076 | 4.48e-01 | 55.8 |
| rs2274316 | A>C | 0.650 | 0.0128 | 0.0020 | 4.80e-10 | 457756 | 0.0132 | 0.0073 | 6.84e-02 | 40.6 |
| rs2275527 | A>G | 0.234 | 0.0151 | 0.0023 | 2.20e-11 | 457756 | -0.0200 | 0.0087 | 2.15e-02 | 43.7 |
| rs2276190 | A>G | 0.271 | 0.0146 | 0.0022 | 6.60e-12 | 457756 | -0.0135 | 0.0074 | 6.95e-02 | 45.3 |
| rs2277158 | G>A | 0.418 | 0.0130 | 0.0019 | 4.80e-11 | 457756 | 0.0063 | 0.0070 | 3.65e-01 | 44.8 |
| rs2279574 | A>C | 0.538 | -0.0151 | 0.0019 | 2.10e-15 | 457756 | -0.0092 | 0.0069 | 1.86e-01 | 60.3 |
| rs2289322 | T>G | 0.147 | 0.0162 | 0.0027 | 9.10e-10 | 457756 | 0.0119 | 0.0100 | 2.33e-01 | 35.6 |
| rs2290081 | T>C | 0.345 | 0.0112 | 0.0020 | 7.40e-09 | 457756 | -0.0071 | 0.0071 | 3.19e-01 | 31.2 |
| rs2292238 | C>A | 0.405 | -0.0162 | 0.0020 | 3.30e-16 | 457756 | 0.0077 | 0.0071 | 2.80e-01 | 67.5 |
| rs2306540 | T>C | 0.307 | 0.0128 | 0.0021 | 5.50e-10 | 457756 | 0.0036 | 0.0079 | 6.43e-01 | 37.5 |
| rs2306593 | T>C | 0.482 | -0.0162 | 0.0019 | 6.00e-17 | 457756 | 0.0077 | 0.0069 | 2.66e-01 | 69.4 |
| rs2307111 | C>T | 0.383 | -0.0284 | 0.0020 | 2.70e-47 | 457756 | -0.0129 | 0.0070 | 6.62e-02 | 203.1 |
| rs2309838 | C>T | 0.401 | -0.0164 | 0.0019 | 3.20e-17 | 457756 | -0.0131 | 0.0070 | 6.05e-02 | 71.3 |
| rs236667 | C>A | 0.586 | 0.0230 | 0.0020 | 2.40e-31 | 457756 | -0.0003 | 0.0147 | 9.84e-01 | 134.4 |
| rs2373284 | C>T | 0.392 | -0.0110 | 0.0020 | 4.50e-08 | 457756 | 0.0007 | 0.0072 | 9.26e-01 | 31.3 |
| rs2385403 | T>C | 0.151 | -0.0199 | 0.0027 | 5.70e-14 | 457756 | -0.0225 | 0.0115 | 5.00e-02 | 55.5 |
| rs2472297 | T>C | 0.263 | 0.0141 | 0.0022 | 8.40e-11 | 457756 | -0.0054 | 0.0091 | 5.52e-01 | 41.3 |
| rs2510344 | C>T | 0.486 | -0.0206 | 0.0019 | 9.90e-27 | 457756 | -0.0050 | 0.0070 | 4.76e-01 | 112.7 |
| rs2609188 | C>T | 0.438 | -0.0132 | 0.0019 | 2.60e-12 | 457756 | -0.0007 | 0.0070 | 9.17e-01 | 47.0 |
| rs2615613 | T>G | 0.082 | -0.0198 | 0.0035 | 3.30e-08 | 457756 | -0.0050 | 0.0122 | 6.84e-01 | 31.3 |
| rs2627323 | T>C | 0.466 | 0.0112 | 0.0019 | 9.90e-09 | 457756 | 0.0155 | 0.0077 | 4.43e-02 | 33.8 |
| rs2733269 | G>A | 0.554 | 0.0125 | 0.0019 | 1.30e-10 | 457756 | 0.0001 | 0.0070 | 9.84e-01 | 42.2 |
| rs2802536 | C>T | 0.822 | -0.0146 | 0.0025 | 5.70e-09 | 457756 | -0.0231 | 0.0098 | 1.85e-02 | 34.3 |
| rs2814992 | G>A | 0.329 | 0.0285 | 0.0020 | 5.00e-45 | 457756 | 0.0068 | 0.0074 | 3.59e-01 | 195.5 |
| rs2815757 | T>C | 0.809 | 0.0321 | 0.0024 | 3.90e-40 | 457756 | 0.0398 | 0.0104 | 1.25e-04 | 175.3 |
| rs2833978 | A>G | 0.102 | -0.0174 | 0.0032 | 1.70e-08 | 457756 | -0.0238 | 0.0135 | 7.74e-02 | 29.5 |
| rs2836754 | C>T | 0.638 | 0.0123 | 0.0020 | 2.90e-10 | 457756 | 0.0122 | 0.0073 | 9.58e-02 | 37.7 |
| rs2838818 | G>A | 0.552 | 0.0159 | 0.0020 | 4.20e-16 | 457756 | 0.0047 | 0.0070 | 4.98e-01 | 65.4 |
| rs28433800 | T>C | 0.396 | 0.0127 | 0.0020 | 7.50e-11 | 457756 | 0.0113 | 0.0073 | 1.19e-01 | 41.6 |
| rs285681 | A>G | 0.186 | 0.0147 | 0.0025 | 4.50e-09 | 457756 | 0.0020 | 0.0100 | 8.38e-01 | 35.1 |
| rs2862996 | T>G | 0.689 | -0.0228 | 0.0021 | 1.00e-28 | 457756 | -0.0171 | 0.0079 | 3.06e-02 | 121.0 |
| rs28726372 | C>T | 0.307 | 0.0115 | 0.0021 | 3.20e-08 | 457756 | 0.0021 | 0.0074 | 7.75e-01 | 30.9 |
| rs288173 | G>A | 0.173 | -0.0241 | 0.0025 | 1.20e-21 | 457756 | -0.0283 | 0.0083 | 6.19e-04 | 90.7 |
| rs2920939 | A>G | 0.607 | 0.0108 | 0.0020 | 3.60e-08 | 457756 | 0.0119 | 0.0073 | 1.02e-01 | 30.2 |
| rs3004928 | C>A | 0.425 | 0.0107 | 0.0020 | 3.20e-08 | 457756 | 0.0004 | 0.0075 | 9.58e-01 | 29.9 |
| rs312925 | A>G | 0.278 | -0.0139 | 0.0021 | 3.70e-11 | 457756 | 0.0037 | 0.0081 | 6.43e-01 | 42.0 |
| rs32415 | T>C | 0.217 | 0.0128 | 0.0023 | 4.20e-08 | 457756 | -0.0040 | 0.0083 | 6.24e-01 | 30.3 |
| rs326851 | A>G | 0.613 | 0.0111 | 0.0020 | 8.00e-09 | 457756 | 0.0021 | 0.0071 | 7.66e-01 | 31.8 |
| rs329080 | C>T | 0.516 | 0.0110 | 0.0019 | 1.80e-08 | 457756 | -0.0071 | 0.0072 | 3.30e-01 | 33.1 |
| rs329118 | T>C | 0.418 | -0.0156 | 0.0019 | 1.60e-16 | 457756 | -0.0234 | 0.0070 | 8.48e-04 | 64.5 |
| rs337638 | C>T | 0.375 | -0.0133 | 0.0020 | 6.20e-11 | 457756 | 0.0025 | 0.0072 | 7.26e-01 | 44.8 |
| rs34153025 | C>T | 0.023 | -0.0384 | 0.0065 | 2.60e-09 | 457756 | -0.0241 | 0.0257 | 3.49e-01 | 34.5 |
| rs34213351 | T>C | 0.077 | 0.0278 | 0.0036 | 6.00e-15 | 457756 | -0.0083 | 0.0115 | 4.72e-01 | 59.9 |
| rs34774377 | C>T | 0.119 | -0.0164 | 0.0030 | 1.50e-08 | 457756 | 0.0181 | 0.0125 | 1.50e-01 | 30.8 |
| rs34811474 | A>G | 0.230 | -0.0271 | 0.0023 | 1.20e-32 | 457756 | -0.0075 | 0.0095 | 4.27e-01 | 134.7 |
| rs35168566 | A>G | 0.556 | 0.0133 | 0.0019 | 2.30e-11 | 457756 | 0.0166 | 0.0074 | 2.47e-02 | 46.6 |
| rs35186116 | G>A | 0.292 | 0.0164 | 0.0021 | 7.80e-15 | 457756 | -0.0057 | 0.0091 | 5.27e-01 | 60.5 |
| rs355754 | C>T | 0.398 | 0.0155 | 0.0020 | 6.00e-15 | 457756 | -0.0051 | 0.0070 | 4.64e-01 | 61.7 |
| rs35678823 | T>C | 0.578 | 0.0107 | 0.0019 | 4.20e-08 | 457756 | 0.0106 | 0.0071 | 1.34e-01 | 30.2 |
| rs35686847 | A>C | 0.648 | -0.0128 | 0.0020 | 4.50e-10 | 457756 | 0.0053 | 0.0072 | 4.62e-01 | 40.7 |
| rs35697691 | G>C | 0.089 | 0.0233 | 0.0034 | 1.40e-11 | 457756 | 0.0436 | 0.0154 | 4.66e-03 | 46.6 |
| rs35917007 | G>A | 0.534 | 0.0142 | 0.0019 | 9.20e-14 | 457756 | -0.0047 | 0.0073 | 5.18e-01 | 54.2 |
| rs364584 | A>C | 0.638 | -0.0120 | 0.0020 | 2.30e-09 | 457756 | -0.0188 | 0.0085 | 2.67e-02 | 35.8 |
| rs3735478 | T>G | 0.289 | -0.0132 | 0.0021 | 3.20e-10 | 457756 | -0.0055 | 0.0079 | 4.85e-01 | 38.1 |
| rs3742305 | G>C | 0.264 | -0.0163 | 0.0022 | 5.80e-14 | 457756 | -0.0056 | 0.0080 | 4.86e-01 | 54.9 |
| rs3751837 | T>C | 0.217 | 0.0150 | 0.0023 | 1.10e-10 | 457756 | -0.0130 | 0.0082 | 1.14e-01 | 41.0 |
| rs3751859 | A>G | 0.157 | -0.0166 | 0.0026 | 4.60e-10 | 457756 | 0.0004 | 0.0087 | 9.63e-01 | 39.4 |
| rs3814058 | C>T | 0.171 | -0.0145 | 0.0025 | 1.00e-08 | 457756 | 0.0133 | 0.0081 | 1.00e-01 | 32.4 |
| rs3814883 | T>C | 0.481 | 0.0237 | 0.0019 | 1.40e-34 | 457756 | 0.0131 | 0.0072 | 6.69e-02 | 148.7 |
| rs3823445 | A>G | 0.290 | -0.0146 | 0.0021 | 2.70e-11 | 457756 | -0.0037 | 0.0073 | 6.08e-01 | 47.2 |
| rs3826408 | T>C | 0.457 | 0.0109 | 0.0019 | 8.40e-09 | 457756 | -0.0131 | 0.0069 | 5.89e-02 | 31.7 |
| rs3895907 | G>A | 0.560 | -0.0172 | 0.0019 | 2.90e-19 | 457756 | -0.0207 | 0.0076 | 6.72e-03 | 79.5 |
| rs3935190 | A>G | 0.531 | -0.0161 | 0.0020 | 2.60e-16 | 457756 | -0.0076 | 0.0070 | 2.79e-01 | 67.2 |
| rs396354 | C>T | 0.712 | -0.0164 | 0.0021 | 9.30e-15 | 457756 | -0.0044 | 0.0084 | 6.05e-01 | 59.1 |
| rs40245 | T>A | 0.636 | -0.0108 | 0.0020 | 4.90e-08 | 457756 | -0.0115 | 0.0081 | 1.55e-01 | 28.5 |
| rs40831 | G>A | 0.425 | 0.0260 | 0.0020 | 5.50e-40 | 457756 | 0.0137 | 0.0072 | 5.57e-02 | 174.8 |
| rs41292864 | A>G | 0.118 | 0.0175 | 0.0030 | 1.30e-08 | 457756 | 0.0097 | 0.0128 | 4.48e-01 | 33.7 |
| rs4148866 | T>C | 0.408 | 0.0112 | 0.0020 | 8.10e-09 | 457756 | 0.0100 | 0.0071 | 1.60e-01 | 32.7 |
| rs4264461 | G>A | 0.537 | 0.0113 | 0.0019 | 2.60e-09 | 457756 | -0.0023 | 0.0074 | 7.58e-01 | 34.6 |
| rs4273371 | C>T | 0.487 | 0.0117 | 0.0019 | 5.00e-10 | 457756 | 0.0139 | 0.0072 | 5.38e-02 | 37.4 |
| rs429358 | C>T | 0.154 | -0.0258 | 0.0027 | 9.90e-22 | 457756 | -0.0481 | 0.0098 | 9.09e-07 | 92.5 |
| rs4322600 | G>A | 0.729 | -0.0125 | 0.0022 | 9.50e-09 | 457756 | -0.0011 | 0.0086 | 8.96e-01 | 33.5 |
| rs4377469 | T>G | 0.887 | 0.0210 | 0.0030 | 4.90e-13 | 457756 | -0.0045 | 0.0097 | 6.47e-01 | 48.4 |
| rs4442149 | T>C | 0.376 | 0.0145 | 0.0020 | 1.10e-13 | 457756 | 0.0006 | 0.0075 | 9.33e-01 | 53.5 |
| rs4449331 | C>T | 0.401 | 0.0137 | 0.0019 | 5.40e-13 | 457756 | 0.0098 | 0.0074 | 1.84e-01 | 49.8 |
| rs4461299 | C>T | 0.372 | 0.0108 | 0.0020 | 4.10e-08 | 457756 | -0.0187 | 0.0076 | 1.42e-02 | 30.2 |
| rs4486004 | T>G | 0.221 | -0.0157 | 0.0025 | 1.80e-10 | 457756 | -0.0025 | 0.0087 | 7.77e-01 | 38.4 |
| rs4487458 | A>G | 0.294 | -0.0119 | 0.0021 | 1.40e-08 | 457756 | -0.0013 | 0.0087 | 8.86e-01 | 32.0 |
| rs4537902 | G>T | 0.491 | 0.0158 | 0.0019 | 1.30e-16 | 457756 | -0.0115 | 0.0073 | 1.18e-01 | 68.0 |
| rs45486197 | A>G | 0.064 | 0.0262 | 0.0042 | 2.50e-10 | 457756 | -0.0233 | 0.0164 | 1.56e-01 | 39.6 |
| rs4569092 | T>C | 0.135 | 0.0189 | 0.0028 | 6.80e-12 | 457756 | -0.0052 | 0.0091 | 5.68e-01 | 45.5 |
| rs4590441 | G>A | 0.876 | 0.0166 | 0.0029 | 1.40e-08 | 457756 | 0.0072 | 0.0104 | 4.86e-01 | 32.4 |
| rs4591326 | C>G | 0.280 | -0.0145 | 0.0021 | 5.20e-12 | 457756 | -0.0022 | 0.0075 | 7.73e-01 | 46.6 |
| rs4643373 | C>T | 0.299 | -0.0208 | 0.0021 | 3.30e-22 | 457756 | 0.0040 | 0.0086 | 6.43e-01 | 96.2 |
| rs4667812 | G>T | 0.245 | -0.0137 | 0.0022 | 2.70e-10 | 457756 | -0.0113 | 0.0078 | 1.47e-01 | 38.2 |
| rs4700645 | G>A | 0.326 | -0.0132 | 0.0020 | 8.50e-11 | 457756 | -0.0051 | 0.0074 | 4.90e-01 | 41.8 |
| rs4717623 | T>C | 0.261 | -0.0163 | 0.0022 | 7.40e-14 | 457756 | 0.0122 | 0.0079 | 1.25e-01 | 55.6 |
| rs4722398 | T>C | 0.136 | 0.0173 | 0.0028 | 6.00e-10 | 457756 | -0.0159 | 0.0114 | 1.62e-01 | 38.5 |
| rs4745062 | T>C | 0.415 | 0.0133 | 0.0019 | 2.70e-12 | 457756 | 0.0164 | 0.0069 | 1.78e-02 | 46.8 |
| rs4764307 | T>C | 0.307 | -0.0121 | 0.0021 | 6.50e-09 | 457756 | 0.0005 | 0.0077 | 9.47e-01 | 33.8 |
| rs4771561 | C>T | 0.402 | -0.0107 | 0.0020 | 1.90e-08 | 457756 | -0.0063 | 0.0070 | 3.68e-01 | 29.5 |
| rs4778879 | G>A | 0.437 | -0.0134 | 0.0019 | 4.00e-12 | 457756 | -0.0172 | 0.0070 | 1.34e-02 | 47.4 |
| rs4808187 | C>A | 0.078 | 0.0208 | 0.0036 | 2.50e-09 | 457756 | 0.0174 | 0.0116 | 1.35e-01 | 33.4 |
| rs4929923 | C>T | 0.645 | 0.0176 | 0.0020 | 1.00e-18 | 457756 | 0.0085 | 0.0072 | 2.36e-01 | 77.0 |
| rs4936175 | C>T | 0.443 | 0.0126 | 0.0019 | 5.90e-11 | 457756 | 0.0210 | 0.0079 | 7.62e-03 | 42.4 |
| rs4952843 | G>A | 0.392 | -0.0149 | 0.0020 | 1.90e-14 | 457756 | 0.0007 | 0.0078 | 9.31e-01 | 58.7 |
| rs4984406 | C>T | 0.508 | -0.0131 | 0.0019 | 3.40e-12 | 457756 | 0.0019 | 0.0069 | 7.81e-01 | 46.6 |
| rs4986041 | C>T | 0.454 | -0.0180 | 0.0019 | 3.60e-20 | 457756 | 0.0005 | 0.0070 | 9.46e-01 | 86.5 |
| rs543874 | G>A | 0.205 | 0.0464 | 0.0024 | 1.70e-87 | 457756 | 0.0104 | 0.0085 | 2.22e-01 | 385.6 |
| rs55726687 | A>G | 0.204 | 0.0250 | 0.0024 | 1.00e-25 | 457756 | -0.0149 | 0.0091 | 1.02e-01 | 106.5 |
| rs55928579 | C>G | 0.117 | -0.0180 | 0.0030 | 1.70e-09 | 457756 | -0.0009 | 0.0113 | 9.38e-01 | 36.4 |
| rs56226325 | T>C | 0.155 | -0.0180 | 0.0026 | 6.70e-12 | 457756 | -0.0352 | 0.0096 | 2.33e-04 | 46.3 |
| rs57163574 | C>A | 0.216 | -0.0140 | 0.0023 | 1.10e-09 | 457756 | -0.0080 | 0.0095 | 4.02e-01 | 35.9 |
| rs5798930 | GT>G | 0.712 | -0.0153 | 0.0021 | 1.60e-12 | 457756 | -0.0080 | 0.0107 | 4.54e-01 | 50.5 |
| rs58634322 | C>T | 0.111 | 0.0167 | 0.0030 | 2.80e-08 | 457756 | 0.0193 | 0.0140 | 1.69e-01 | 30.3 |
| rs58873369 | G>C | 0.064 | 0.0295 | 0.0039 | 2.40e-14 | 457756 | -0.0079 | 0.0151 | 5.99e-01 | 57.2 |
| rs594024 | C>T | 0.554 | -0.0141 | 0.0019 | 4.00e-13 | 457756 | 0.0012 | 0.0096 | 8.98e-01 | 53.6 |
| rs5995843 | G>A | 0.345 | -0.0170 | 0.0020 | 4.40e-16 | 457756 | -0.0112 | 0.0071 | 1.14e-01 | 69.3 |
| rs6038557 | A>G | 0.629 | 0.0208 | 0.0020 | 2.10e-25 | 457756 | 0.0037 | 0.0078 | 6.35e-01 | 108.3 |
| rs60654199 | A>C | 0.067 | 0.0332 | 0.0038 | 2.30e-18 | 457756 | -0.0082 | 0.0111 | 4.61e-01 | 75.1 |
| rs6069039 | A>G | 0.721 | -0.0129 | 0.0021 | 1.60e-09 | 457756 | 0.0039 | 0.0139 | 7.78e-01 | 37.5 |
| rs6096949 | T>C | 0.185 | -0.0259 | 0.0025 | 9.10e-25 | 457756 | -0.0022 | 0.0093 | 8.14e-01 | 109.0 |
| rs61223906 | A>G | 0.340 | -0.0129 | 0.0020 | 4.50e-11 | 457756 | -0.0099 | 0.0072 | 1.70e-01 | 40.4 |
| rs61587156 | G>T | 0.185 | -0.0293 | 0.0025 | 1.00e-31 | 457756 | -0.0121 | 0.0103 | 2.40e-01 | 141.5 |
| rs61813293 | T>G | 0.143 | 0.0242 | 0.0027 | 2.20e-19 | 457756 | 0.0142 | 0.0114 | 2.11e-01 | 79.4 |
| rs61971082 | G>T | 0.284 | 0.0153 | 0.0021 | 1.70e-12 | 457756 | 0.0083 | 0.0083 | 3.22e-01 | 51.6 |
| rs62092069 | A>G | 0.371 | -0.0142 | 0.0020 | 5.10e-13 | 457756 | 0.0009 | 0.0071 | 8.94e-01 | 51.1 |
| rs62114506 | C>G | 0.259 | -0.0128 | 0.0022 | 3.60e-09 | 457756 | -0.0025 | 0.0079 | 7.54e-01 | 33.0 |
| rs62192064 | G>C | 0.387 | -0.0113 | 0.0020 | 7.50e-09 | 457756 | -0.0100 | 0.0076 | 1.84e-01 | 33.1 |
| rs62253188 | A>G | 0.319 | 0.0111 | 0.0020 | 4.40e-08 | 457756 | 0.0044 | 0.0075 | 5.52e-01 | 29.4 |
| rs6235 | G>C | 0.268 | 0.0173 | 0.0022 | 2.40e-15 | 457756 | 0.0062 | 0.0079 | 4.31e-01 | 63.5 |
| rs62422661 | T>G | 0.393 | -0.0173 | 0.0020 | 4.70e-19 | 457756 | -0.0094 | 0.0071 | 1.90e-01 | 77.6 |
| rs6265 | T>C | 0.188 | -0.0404 | 0.0025 | 2.40e-60 | 457756 | -0.0047 | 0.0083 | 5.70e-01 | 268.8 |
| rs6426066 | C>T | 0.777 | -0.0130 | 0.0023 | 1.60e-08 | 457756 | -0.0061 | 0.0085 | 4.75e-01 | 32.1 |
| rs6442548 | G>A | 0.679 | -0.0112 | 0.0020 | 3.90e-08 | 457756 | -0.0218 | 0.0082 | 8.08e-03 | 30.3 |
| rs645040 | T>G | 0.773 | 0.0189 | 0.0023 | 6.10e-17 | 457756 | 0.0178 | 0.0087 | 4.01e-02 | 69.0 |
| rs6461112 | T>C | 0.974 | -0.0330 | 0.0061 | 2.70e-08 | 457756 | 0.0106 | 0.0280 | 7.05e-01 | 29.5 |
| rs6471352 | T>C | 0.535 | 0.0118 | 0.0019 | 4.70e-10 | 457756 | 0.0063 | 0.0070 | 3.63e-01 | 37.0 |
| rs6479500 | G>A | 0.678 | -0.0159 | 0.0021 | 2.50e-14 | 457756 | -0.0026 | 0.0073 | 7.22e-01 | 58.5 |
| rs652852 | T>C | 0.392 | -0.0137 | 0.0020 | 1.70e-11 | 457756 | 0.0076 | 0.0070 | 2.80e-01 | 48.5 |
| rs6545695 | A>G | 0.388 | -0.0185 | 0.0020 | 3.50e-21 | 457756 | 0.0006 | 0.0070 | 9.30e-01 | 89.4 |
| rs6548238 | C>T | 0.828 | 0.0562 | 0.0025 | 2.10e-111 | 457756 | 0.0108 | 0.0097 | 2.66e-01 | 495.9 |
| rs6555936 | A>G | 0.697 | 0.0173 | 0.0021 | 6.90e-17 | 457756 | -0.0015 | 0.0082 | 8.58e-01 | 68.1 |
| rs6559365 | A>G | 0.498 | 0.0124 | 0.0019 | 8.40e-11 | 457756 | 0.0355 | 0.0070 | 3.67e-07 | 41.3 |
| rs6591 | T>C | 0.540 | 0.0133 | 0.0019 | 4.00e-11 | 457756 | -0.0074 | 0.0071 | 2.98e-01 | 46.7 |
| rs6597173 | T>C | 0.673 | 0.0117 | 0.0020 | 2.00e-08 | 457756 | 0.0002 | 0.0073 | 9.82e-01 | 32.8 |
| rs6669341 | G>A | 0.583 | -0.0162 | 0.0019 | 3.60e-17 | 457756 | 0.0013 | 0.0080 | 8.71e-01 | 70.1 |
| rs6700863 | C>G | 0.832 | -0.0179 | 0.0026 | 2.90e-12 | 457756 | -0.0194 | 0.0090 | 3.19e-02 | 48.7 |
| rs6706613 | C>T | 0.837 | -0.0210 | 0.0026 | 1.70e-15 | 457756 | -0.0035 | 0.0096 | 7.16e-01 | 66.6 |
| rs6715082 | C>T | 0.346 | 0.0161 | 0.0020 | 9.00e-16 | 457756 | 0.0140 | 0.0073 | 5.53e-02 | 64.4 |
| rs6720868 | T>C | 0.316 | 0.0169 | 0.0021 | 6.50e-16 | 457756 | -0.0133 | 0.0076 | 8.17e-02 | 67.6 |
| rs6738833 | C>T | 0.514 | 0.0122 | 0.0019 | 2.20e-10 | 457756 | 0.0137 | 0.0075 | 6.86e-02 | 40.7 |
| rs6751741 | C>T | 0.561 | -0.0114 | 0.0019 | 9.70e-10 | 457756 | 0.0085 | 0.0080 | 2.86e-01 | 35.2 |
| rs67602344 | A>C | 0.192 | -0.0219 | 0.0024 | 2.40e-19 | 457756 | -0.0111 | 0.0103 | 2.83e-01 | 79.9 |
| rs6764926 | C>T | 0.548 | -0.0212 | 0.0019 | 8.40e-29 | 457756 | -0.0284 | 0.0073 | 1.03e-04 | 122.1 |
| rs67776659 | C>T | 0.153 | 0.0156 | 0.0027 | 3.40e-09 | 457756 | 0.0312 | 0.0109 | 4.15e-03 | 34.7 |
| rs6790717 | G>A | 0.540 | 0.0105 | 0.0019 | 2.50e-08 | 457756 | 0.0017 | 0.0070 | 8.04e-01 | 30.4 |
| rs6831020 | A>C | 0.294 | -0.0157 | 0.0021 | 2.00e-14 | 457756 | -0.0051 | 0.0085 | 5.50e-01 | 55.5 |
| rs6876883 | A>G | 0.436 | -0.0131 | 0.0019 | 4.80e-11 | 457756 | 0.0158 | 0.0069 | 2.27e-02 | 45.8 |
| rs6880789 | G>A | 0.464 | -0.0110 | 0.0019 | 6.20e-09 | 457756 | -0.0097 | 0.0071 | 1.74e-01 | 33.0 |
| rs694786 | C>T | 0.540 | 0.0159 | 0.0019 | 9.40e-17 | 457756 | 0.0157 | 0.0070 | 2.47e-02 | 68.6 |
| rs6950388 | A>G | 0.796 | 0.0144 | 0.0024 | 6.50e-10 | 457756 | 0.0050 | 0.0081 | 5.35e-01 | 36.7 |
| rs7013560 | C>T | 0.477 | -0.0120 | 0.0019 | 6.40e-10 | 457756 | -0.0018 | 0.0074 | 8.07e-01 | 39.4 |
| rs705165 | T>G | 0.248 | 0.0157 | 0.0022 | 1.30e-12 | 457756 | 0.0007 | 0.0079 | 9.24e-01 | 49.7 |
| rs7078160 | A>G | 0.160 | 0.0155 | 0.0026 | 4.50e-09 | 457756 | 0.0083 | 0.0084 | 3.28e-01 | 35.0 |
| rs7098100 | A>G | 0.340 | 0.0202 | 0.0020 | 3.10e-23 | 457756 | 0.0342 | 0.0083 | 4.04e-05 | 99.5 |
| rs710985 | T>C | 0.521 | -0.0119 | 0.0019 | 1.90e-10 | 457756 | -0.0027 | 0.0069 | 6.93e-01 | 38.9 |
| rs7124681 | A>C | 0.408 | 0.0256 | 0.0020 | 2.20e-39 | 457756 | 0.0143 | 0.0074 | 5.26e-02 | 170.7 |
| rs7132908 | A>G | 0.385 | 0.0294 | 0.0020 | 3.50e-51 | 457756 | -0.0047 | 0.0072 | 5.17e-01 | 221.6 |
| rs713586 | C>T | 0.489 | 0.0337 | 0.0019 | 2.70e-71 | 457756 | 0.0107 | 0.0069 | 1.23e-01 | 311.8 |
| rs71567659 | A>G | 0.138 | -0.0206 | 0.0028 | 1.20e-13 | 457756 | -0.0122 | 0.0121 | 3.13e-01 | 54.9 |
| rs71627581 | A>G | 0.111 | -0.0209 | 0.0031 | 1.40e-11 | 457756 | -0.0186 | 0.0119 | 1.19e-01 | 46.1 |
| rs7170035 | G>A | 0.226 | -0.0154 | 0.0023 | 2.90e-11 | 457756 | -0.0195 | 0.0083 | 1.81e-02 | 44.6 |
| rs7170185 | A>T | 0.224 | -0.0299 | 0.0023 | 4.30e-41 | 457756 | -0.0112 | 0.0081 | 1.66e-01 | 166.7 |
| rs7211567 | T>C | 0.216 | -0.0158 | 0.0023 | 3.40e-11 | 457756 | -0.0119 | 0.0078 | 1.28e-01 | 45.7 |
| rs7230932 | A>G | 0.370 | -0.0114 | 0.0020 | 2.00e-08 | 457756 | 0.0026 | 0.0071 | 7.21e-01 | 32.4 |
| rs72634826 | A>G | 0.252 | -0.0199 | 0.0023 | 5.70e-19 | 457756 | -0.0103 | 0.0094 | 2.76e-01 | 77.3 |
| rs72705206 | T>C | 0.026 | 0.0629 | 0.0061 | 4.10e-25 | 457756 | 0.0372 | 0.0219 | 8.98e-02 | 106.6 |
| rs72769581 | C>T | 0.212 | -0.0205 | 0.0023 | 3.00e-18 | 457756 | -0.0047 | 0.0085 | 5.81e-01 | 76.2 |
| rs72948836 | G>A | 0.058 | -0.0231 | 0.0041 | 2.90e-08 | 457756 | -0.0010 | 0.0123 | 9.35e-01 | 31.7 |
| rs73024820 | G>A | 0.159 | -0.0229 | 0.0026 | 1.30e-18 | 457756 | -0.0036 | 0.0111 | 7.44e-01 | 75.7 |
| rs73215408 | T>C | 0.137 | -0.0198 | 0.0028 | 3.90e-12 | 457756 | -0.0110 | 0.0104 | 2.92e-01 | 50.8 |
| rs7323 | C>G | 0.280 | -0.0176 | 0.0022 | 2.20e-15 | 457756 | -0.0094 | 0.0087 | 2.78e-01 | 66.4 |
| rs7325555 | T>C | 0.426 | -0.0133 | 0.0019 | 7.20e-12 | 457756 | -0.0013 | 0.0073 | 8.54e-01 | 47.0 |
| rs7332115 | G>T | 0.371 | -0.0169 | 0.0020 | 2.70e-17 | 457756 | -0.0113 | 0.0075 | 1.33e-01 | 72.3 |
| rs73601548 | T>C | 0.114 | 0.0182 | 0.0030 | 1.20e-09 | 457756 | 0.0002 | 0.0129 | 9.88e-01 | 36.1 |
| rs73837032 | A>G | 0.209 | 0.0157 | 0.0024 | 3.20e-11 | 457756 | 0.0050 | 0.0096 | 6.02e-01 | 44.4 |
| rs7420531 | A>G | 0.539 | 0.0106 | 0.0019 | 1.10e-08 | 457756 | -0.0110 | 0.0073 | 1.32e-01 | 30.5 |
| rs7422777 | T>C | 0.526 | -0.0120 | 0.0019 | 1.50e-10 | 457756 | -0.0057 | 0.0069 | 4.10e-01 | 39.6 |
| rs7432838 | T>C | 0.843 | 0.0195 | 0.0027 | 1.30e-13 | 457756 | 0.0263 | 0.0105 | 1.23e-02 | 54.3 |
| rs7451021 | C>T | 0.685 | -0.0112 | 0.0021 | 4.10e-08 | 457756 | -0.0082 | 0.0077 | 2.85e-01 | 28.8 |
| rs74579064 | A>C | 0.096 | 0.0181 | 0.0032 | 3.10e-08 | 457756 | 0.0060 | 0.0118 | 6.10e-01 | 31.4 |
| rs7488867 | T>C | 0.252 | -0.0198 | 0.0022 | 3.20e-19 | 457756 | 0.0024 | 0.0076 | 7.54e-01 | 80.0 |
| rs74934567 | G>A | 0.154 | -0.0227 | 0.0026 | 6.20e-18 | 457756 | 0.0020 | 0.0093 | 8.33e-01 | 73.4 |
| rs7552982 | C>T | 0.132 | 0.0186 | 0.0028 | 2.40e-11 | 457756 | 0.0184 | 0.0092 | 4.52e-02 | 43.5 |
| rs75810210 | T>C | 0.089 | -0.0322 | 0.0034 | 7.10e-21 | 457756 | -0.0096 | 0.0142 | 4.96e-01 | 90.8 |
| rs76111507 | T>C | 0.037 | -0.0905 | 0.0051 | 9.70e-73 | 457756 | -0.0310 | 0.0232 | 1.81e-01 | 320.2 |
| rs7617565 | T>C | 0.263 | 0.0149 | 0.0022 | 5.80e-12 | 457756 | 0.0078 | 0.0094 | 4.06e-01 | 47.4 |
| rs7618975 | C>A | 0.390 | -0.0135 | 0.0020 | 3.50e-12 | 457756 | -0.0071 | 0.0072 | 3.20e-01 | 47.6 |
| rs76223526 | T>C | 0.049 | 0.0291 | 0.0045 | 5.40e-11 | 457756 | 0.0250 | 0.0178 | 1.61e-01 | 42.5 |
| rs7663212 | G>A | 0.250 | -0.0138 | 0.0022 | 2.00e-10 | 457756 | -0.0048 | 0.0078 | 5.42e-01 | 38.8 |
| rs7686195 | C>T | 0.548 | -0.0127 | 0.0019 | 2.70e-11 | 457756 | -0.0036 | 0.0069 | 6.03e-01 | 43.5 |
| rs7719067 | G>A | 0.572 | -0.0160 | 0.0019 | 5.80e-17 | 457756 | -0.0016 | 0.0080 | 8.37e-01 | 68.5 |
| rs7721099 | C>T | 0.134 | 0.0334 | 0.0028 | 2.70e-33 | 457756 | 0.0243 | 0.0118 | 3.96e-02 | 145.6 |
| rs77483079 | T>C | 0.155 | -0.0251 | 0.0027 | 6.00e-21 | 457756 | -0.0041 | 0.0093 | 6.59e-01 | 89.4 |
| rs7762794 | G>A | 0.285 | 0.0144 | 0.0021 | 7.60e-12 | 457756 | 0.0102 | 0.0074 | 1.67e-01 | 45.7 |
| rs7822013 | C>T | 0.441 | 0.0109 | 0.0019 | 1.50e-08 | 457756 | 0.0077 | 0.0069 | 2.71e-01 | 31.6 |
| rs7869771 | C>A | 0.280 | -0.0152 | 0.0021 | 3.00e-12 | 457756 | 0.0053 | 0.0077 | 4.90e-01 | 50.8 |
| rs7871866 | C>G | 0.152 | 0.0187 | 0.0027 | 7.10e-13 | 457756 | 0.0240 | 0.0091 | 8.70e-03 | 48.0 |
| rs7903146 | T>C | 0.290 | -0.0161 | 0.0021 | 8.30e-15 | 457756 | -0.0056 | 0.0088 | 5.25e-01 | 58.4 |
| rs79186842 | G>A | 0.147 | -0.0209 | 0.0027 | 5.10e-14 | 457756 | -0.0080 | 0.0112 | 4.71e-01 | 58.1 |
| rs79950910 | A>G | 0.132 | 0.0174 | 0.0028 | 1.70e-10 | 457756 | 0.0232 | 0.0104 | 2.62e-02 | 38.1 |
| rs79966207 | C>T | 0.174 | 0.0154 | 0.0026 | 2.20e-09 | 457756 | 0.0046 | 0.0098 | 6.42e-01 | 34.3 |
| rs8015400 | A>C | 0.678 | 0.0215 | 0.0021 | 5.30e-26 | 457756 | 0.0013 | 0.0075 | 8.65e-01 | 109.2 |
| rs8022504 | C>A | 0.085 | 0.0208 | 0.0035 | 3.30e-09 | 457756 | 0.0040 | 0.0141 | 7.79e-01 | 36.4 |
| rs8079754 | G>A | 0.192 | 0.0190 | 0.0025 | 3.00e-15 | 457756 | 0.0326 | 0.0082 | 7.09e-05 | 59.7 |
| rs8086318 | A>G | 0.579 | 0.0137 | 0.0020 | 4.10e-12 | 457756 | 0.0157 | 0.0081 | 5.41e-02 | 48.4 |
| rs8097783 | A>G | 0.073 | -0.0397 | 0.0037 | 1.40e-26 | 457756 | -0.0037 | 0.0124 | 7.67e-01 | 115.1 |
| rs8102137 | C>T | 0.329 | 0.0203 | 0.0020 | 5.30e-23 | 457756 | -0.0008 | 0.0080 | 9.22e-01 | 98.6 |
| rs879620 | T>C | 0.611 | 0.0231 | 0.0020 | 3.10e-32 | 457756 | 0.0070 | 0.0071 | 3.27e-01 | 136.2 |
| rs881301 | C>T | 0.412 | 0.0116 | 0.0019 | 3.10e-09 | 457756 | 0.0038 | 0.0070 | 5.88e-01 | 35.7 |
| rs883403 | C>T | 0.154 | -0.0238 | 0.0027 | 2.80e-18 | 457756 | -0.0008 | 0.0085 | 9.26e-01 | 79.1 |
| rs9257274 | C>T | 0.215 | -0.0249 | 0.0035 | 7.80e-13 | 457756 | -0.0053 | 0.0080 | 5.10e-01 | 50.9 |
| rs9288354 | G>A | 0.907 | -0.0264 | 0.0033 | 7.20e-16 | 457756 | 0.0178 | 0.0147 | 2.27e-01 | 64.5 |
| rs9288851 | G>A | 0.176 | 0.0160 | 0.0025 | 1.30e-10 | 457756 | 0.0167 | 0.0107 | 1.19e-01 | 40.7 |
| rs9382043 | A>G | 0.305 | 0.0150 | 0.0021 | 4.40e-13 | 457756 | 0.0082 | 0.0075 | 2.80e-01 | 51.3 |
| rs9384679 | T>C | 0.371 | -0.0172 | 0.0020 | 1.80e-18 | 457756 | 0.0072 | 0.0072 | 3.20e-01 | 75.4 |
| rs9387289 | T>C | 0.370 | -0.0138 | 0.0020 | 5.20e-12 | 457756 | -0.0174 | 0.0072 | 1.55e-02 | 48.5 |
| rs942650 | C>T | 0.317 | -0.0136 | 0.0021 | 8.10e-11 | 457756 | -0.0085 | 0.0085 | 3.21e-01 | 43.3 |
| rs9491202 | G>A | 0.165 | 0.0144 | 0.0026 | 9.20e-09 | 457756 | -0.0057 | 0.0108 | 5.98e-01 | 30.8 |
| rs9515446 | G>A | 0.448 | 0.0146 | 0.0019 | 2.40e-14 | 457756 | 0.0126 | 0.0069 | 6.94e-02 | 56.6 |
| rs9544948 | A>G | 0.415 | 0.0136 | 0.0019 | 1.20e-11 | 457756 | -0.0010 | 0.0099 | 9.23e-01 | 48.6 |
| rs9821675 | G>A | 0.505 | 0.0272 | 0.0019 | 6.90e-47 | 457756 | 0.0323 | 0.0075 | 1.47e-05 | 202.2 |
| rs9832622 | A>G | 0.350 | -0.0126 | 0.0020 | 4.80e-10 | 457756 | -0.0171 | 0.0072 | 1.79e-02 | 39.8 |
| rs987237 | G>A | 0.180 | 0.0379 | 0.0025 | 1.10e-52 | 457756 | 0.0105 | 0.0087 | 2.28e-01 | 229.8 |
| rs9888368 | A>G | 0.159 | 0.0154 | 0.0026 | 4.20e-09 | 457756 | -0.0109 | 0.0091 | 2.27e-01 | 34.2 |
| rs9940128 | A>G | 0.423 | 0.0726 | 0.0019 | 1.00e-200 | 457756 | 0.0180 | 0.0072 | 1.25e-02 | 1399.1 |

Abbreviations: SNP = single-nucleotide polymorphism; EAF = effect allele frequency; BMI = body mass index; β = beta coefficient (log odds per effect allele); SE = standard error; P = P value; N = sample size. Effect alleles are aligned to the BMI-increasing allele; the F-statistic was computed as (β/SE)². All 361 instruments had F > 10 (mean = 68.4, minimum = 28.5).
