# Supplementary material for: The influence of high body mass index on asthma: A study integrating GBD 2021 and Mendelian randomization
Source: Medicine (Baltimore). 2026 Nov 6;105(45):e51490. doi: 10.1097/MD.0000000000051490 (PMC13747491; doi:10.1097/MD.0000000000051490)
Supplement: Supplementary file 2 [file medi-105-e51490-s002.docx]

**Table S2.** Number of asthma deaths attributable to high BMI across 204 countries and territories, with EAPC (1990–2021).

| **location** | **1990** | | **2021** | | **1990-2021** |
| --- | --- | --- | --- | --- | --- |
|  | **Deaths case（95%UI）** | **ASMR per 100,000(95%UI)** | **Deaths case（95%UI）** | **ASMR per 100,000(95%UI)** | **EAPC (95%CI)** |
| Afghanistan | 446.16 (184.07-805.79) | 6.90 (2.86-12.47) | 439.99 (183.72-815.69) | 5.07 (2.05-9.92) | -1.34 (-1.58 to -1.10) |
| Albania | 51.73 (23.16-81.09) | 3.01 (1.34-4.73) | 41.72 (17.64-72.69) | 0.98 (0.42-1.73) | -3.95 (-4.39 to -3.52) |
| Algeria | 280.68 (115.88-472.25) | 3.15 (1.28-5.40) | 634.85 (291.19-1033.72) | 2.31 (1.03-3.74) | -0.86 (-0.91 to -0.80) |
| American Samoa | 1.14 (0.53-1.78) | 5.82 (2.68-9.18) | 1.24 (0.62-1.87) | 3.11 (1.51-4.71) | -2.39 (-2.59 to -2.18) |
| Andorra | 0.22 (0.09-0.38) | 0.41 (0.17-0.71) | 0.22 (0.09-0.39) | 0.14 (0.06-0.25) | -3.38 (-3.71 to -3.04) |
| Angola | 78.99 (34.48-150.99) | 1.85 (0.74-4.14) | 190.83 (78.33-350.73) | 1.60 (0.59-3.11) | -0.83 (-0.95 to -0.70) |
| Antigua and Barbuda | 0.34 (0.15-0.53) | 0.61 (0.27-0.97) | 0.61 (0.29-0.95) | 0.60 (0.28-0.94) | -0.57 (-0.99 to -0.15) |
| Argentina | 165.17 (75.63-263.70) | 0.52 (0.24-0.83) | 127.67 (59.54-195.44) | 0.23 (0.11-0.35) | -3.02 (-3.44 to -2.59) |
| Armenia | 7.92 (3.36-12.42) | 0.30 (0.13-0.47) | 4.51 (1.97-6.99) | 0.10 (0.05-0.16) | -3.49 (-3.84 to -3.15) |
| Australia | 175.14 (76.09-277.53) | 0.92 (0.40-1.46) | 138.37 (64.73-211.26) | 0.31 (0.15-0.47) | -3.52 (-4.22 to -2.82) |
| Austria | 88.64 (39.72-137.87) | 0.74 (0.33-1.15) | 25.60 (11.26-40.17) | 0.13 (0.06-0.21) | -6.12 (-6.59 to -5.66) |
| Azerbaijan | 104.06 (46.19-173.46) | 2.24 (0.98-3.85) | 93.69 (41.55-159.16) | 1.02 (0.46-1.72) | -3.06 (-3.27 to -2.84) |
| Bahamas | 1.24 (0.54-1.92) | 0.74 (0.32-1.15) | 2.39 (1.07-3.78) | 0.59 (0.26-0.93) | -1.39 (-1.71 to -1.08) |
| Bahrain | 5.27 (2.42-8.28) | 4.23 (1.96-6.84) | 11.09 (5.31-17.97) | 2.16 (0.97-3.60) | -2.87 (-3.33 to -2.42) |
| Bangladesh | 549.86 (237.06-982.36) | 1.14 (0.48-2.08) | 1274.11 (491.39-2514.45) | 0.93 (0.36-1.84) | -0.58 (-0.73 to -0.43) |
| Barbados | 2.67 (1.19-4.22) | 0.93 (0.41-1.46) | 3.63 (1.76-5.97) | 0.74 (0.36-1.21) | -1.06 (-1.48 to -0.63) |
| Belarus | 93.81 (40.06-153.60) | 0.74 (0.32-1.21) | 11.46 (5.03-18.41) | 0.07 (0.03-0.12) | -8.91 (-9.66 to -8.15) |
| Belgium | 92.01 (39.80-144.53) | 0.62 (0.27-0.98) | 36.34 (15.88-56.49) | 0.15 (0.07-0.23) | -5.23 (-5.61 to -4.86) |
| Belize | 0.78 (0.38-1.24) | 0.75 (0.34-1.20) | 2.46 (1.19-3.71) | 0.74 (0.35-1.13) | -0.73 (-1.36 to -0.10) |
| Benin | 57.08 (25.41-98.53) | 2.76 (1.18-4.80) | 105.93 (45.40-201.43) | 1.98 (0.83-3.84) | -0.97 (-1.21 to -0.73) |
| Bermuda | 0.45 (0.19-0.70) | 0.74 (0.32-1.16) | 0.41 (0.20-0.62) | 0.30 (0.15-0.46) | -3.57 (-4.10 to -3.03) |
| Bhutan | 7.08 (2.77-13.33) | 2.92 (1.12-5.54) | 12.55 (4.78-26.60) | 2.15 (0.80-4.53) | -1.20 (-1.30 to -1.09) |
| Bolivia (Plurinational State of) | 31.32 (17.03-49.44) | 0.79 (0.37-1.30) | 32.98 (15.22-54.05) | 0.39 (0.18-0.63) | -2.34 (-2.42 to -2.26) |
| Bosnia and Herzegovina | 20.69 (8.37-37.33) | 0.57 (0.23-1.04) | 16.12 (7.55-26.08) | 0.25 (0.12-0.41) | -3.21 (-3.52 to -2.90) |
| Botswana | 20.99 (7.65-42.89) | 4.13 (1.52-8.43) | 42.70 (17.33-76.41) | 3.28 (1.37-5.86) | -0.58 (-0.82 to -0.34) |
| Brazil | 454.21 (214.70-716.92) | 0.53 (0.24-0.85) | 703.08 (312.67-1092.97) | 0.29 (0.13-0.45) | -2.60 (-2.95 to -2.26) |
| Brunei Darussalam | 1.73 (0.78-2.76) | 1.68 (0.74-2.72) | 2.61 (1.15-4.22) | 0.86 (0.39-1.43) | -1.81 (-2.01 to -1.60) |
| Bulgaria | 68.94 (30.11-110.13) | 0.66 (0.29-1.05) | 13.75 (5.78-21.60) | 0.10 (0.04-0.15) | -7.19 (-7.71 to -6.66) |
| Burkina Faso | 44.21 (22.82-72.68) | 0.95 (0.46-1.62) | 80.82 (37.71-134.37) | 0.79 (0.36-1.31) | -0.61 (-0.80 to -0.42) |
| Burundi | 32.78 (14.94-55.88) | 1.27 (0.53-2.29) | 48.31 (19.46-94.20) | 0.96 (0.34-2.03) | -1.39 (-1.65 to -1.12) |
| Cabo Verde | 5.07 (2.01-8.82) | 2.22 (0.88-3.87) | 5.17 (2.14-9.31) | 1.16 (0.49-2.09) | -1.98 (-2.61 to -1.35) |
| Cambodia | 43.66 (19.65-70.45) | 1.03 (0.45-1.68) | 104.48 (47.24-185.02) | 0.95 (0.44-1.67) | -0.38 (-0.44 to -0.33) |
| Cameroon | 178.21 (81.66-301.92) | 4.10 (1.83-6.94) | 368.32 (165.28-635.29) | 2.95 (1.30-5.17) | -0.95 (-1.03 to -0.86) |
| Canada | 116.46 (52.34-181.00) | 0.36 (0.17-0.57) | 83.25 (37.66-129.23) | 0.13 (0.06-0.19) | -3.88 (-4.32 to -3.44) |
| Central African Republic | 28.29 (11.21-59.79) | 2.41 (0.89-6.17) | 74.34 (26.75-193.20) | 3.25 (1.08-9.49) | 0.88 (0.80 to 0.95) |
| Chad | 55.73 (23.17-94.82) | 2.02 (0.82-3.53) | 112.00 (46.87-196.58) | 1.90 (0.75-3.44) | -0.15 (-0.22 to -0.07) |
| Chile | 68.15 (31.08-105.98) | 0.70 (0.32-1.09) | 73.07 (33.14-113.03) | 0.28 (0.13-0.44) | -2.83 (-3.00 to -2.67) |
| China | 2959.97 (1224.91-5041.93) | 0.47 (0.19-0.84) | 4321.91 (1949.72-7146.99) | 0.24 (0.11-0.39) | -2.52 (-2.74 to -2.30) |
| Colombia | 118.63 (54.27-184.77) | 0.71 (0.32-1.10) | 62.84 (28.82-99.93) | 0.11 (0.05-0.18) | -7.04 (-7.44 to -6.63) |
| Comoros | 3.73 (1.59-6.46) | 1.76 (0.69-3.25) | 7.91 (3.35-14.75) | 1.64 (0.65-3.13) | -0.54 (-0.82 to -0.26) |
| Congo | 31.74 (11.77-66.40) | 2.98 (1.05-6.83) | 64.72 (27.32-118.84) | 2.42 (1.00-4.67) | -1.01 (-1.19 to -0.83) |
| Cook Islands | 0.39 (0.17-0.64) | 3.60 (1.57-6.06) | 0.38 (0.17-0.62) | 1.57 (0.72-2.62) | -2.91 (-3.07 to -2.75) |
| Costa Rica | 10.55 (5.07-16.58) | 0.60 (0.28-0.95) | 12.31 (5.70-19.41) | 0.22 (0.10-0.35) | -4.14 (-4.79 to -3.48) |
| Croatia | 63.66 (28.46-102.10) | 1.16 (0.52-1.86) | 21.81 (9.12-34.90) | 0.22 (0.09-0.35) | -6.39 (-7.12 to -5.65) |
| Cuba | 55.49 (23.99-84.96) | 0.54 (0.23-0.82) | 81.39 (36.23-129.05) | 0.45 (0.20-0.70) | -1.05 (-1.42 to -0.69) |
| Cyprus | 8.01 (3.44-13.88) | 1.34 (0.56-2.26) | 8.82 (4.03-14.47) | 0.51 (0.23-0.83) | -3.53 (-3.79 to -3.27) |
| Czechia | 81.29 (36.92-124.24) | 0.60 (0.27-0.91) | 36.89 (16.03-56.77) | 0.16 (0.07-0.24) | -4.44 (-5.20 to -3.68) |
| Coate d'Ivoire | 108.23 (45.72-180.44) | 2.72 (1.17-4.64) | 232.57 (101.17-408.81) | 2.06 (0.86-3.63) | -0.76 (-0.85 to -0.67) |
| Democratic People's Republic of Korea | 89.27 (33.18-191.91) | 0.76 (0.27-1.74) | 227.86 (86.40-480.63) | 0.81 (0.30-1.79) | 0.26 (0.22 to 0.31) |
| Democratic Republic of the Congo | 287.61 (105.63-686.44) | 1.96 (0.66-5.41) | 969.37 (340.21-2633.05) | 2.91 (0.94-8.77) | 1.32 (1.27 to 1.38) |
| Denmark | 50.02 (22.38-80.28) | 0.64 (0.29-1.02) | 21.60 (9.52-34.36) | 0.18 (0.08-0.28) | -5.22 (-5.74 to -4.69) |
| Djibouti | 1.23 (0.57-2.08) | 0.80 (0.34-1.43) | 4.24 (1.65-7.84) | 0.66 (0.25-1.24) | -0.81 (-0.91 to -0.72) |
| Dominica | 0.87 (0.39-1.36) | 1.49 (0.65-2.32) | 1.20 (0.56-1.88) | 1.49 (0.70-2.33) | -0.20 (-0.31 to -0.09) |
| Dominican Republic | 53.14 (26.03-84.15) | 1.37 (0.64-2.20) | 88.51 (38.53-155.97) | 0.86 (0.38-1.52) | -1.17 (-1.37 to -0.97) |
| Ecuador | 39.87 (18.67-60.37) | 0.73 (0.33-1.14) | 16.88 (7.63-26.88) | 0.11 (0.05-0.17) | -5.52 (-5.98 to -5.06) |
| Egypt | 1218.98 (549.98-1950.42) | 5.52 (2.32-9.25) | 1129.93 (542.80-1745.55) | 2.40 (1.13-3.71) | -2.83 (-3.06 to -2.60) |
| El Salvador | 49.68 (20.34-79.01) | 1.66 (0.66-2.68) | 46.90 (21.67-78.31) | 0.71 (0.33-1.18) | -3.40 (-3.68 to -3.11) |
| Equatorial Guinea | 6.50 (2.73-13.21) | 3.23 (1.20-7.05) | 9.26 (3.68-18.73) | 1.81 (0.72-3.67) | -2.48 (-2.75 to -2.21) |
| Eritrea | 12.74 (5.93-20.65) | 0.96 (0.41-1.65) | 29.80 (12.69-51.01) | 1.02 (0.41-1.80) | 0.21 (0.10 to 0.32) |
| Estonia | 24.41 (10.66-38.50) | 1.19 (0.52-1.87) | 5.00 (2.17-8.08) | 0.17 (0.07-0.27) | -6.91 (-7.23 to -6.58) |
| Eswatini | 18.26 (7.06-35.33) | 6.95 (2.55-13.84) | 35.93 (14.28-63.85) | 6.77 (2.63-12.03) | 0.31 (-0.21 to 0.83) |
| Ethiopia | 246.92 (112.53-406.31) | 1.11 (0.48-1.86) | 270.47 (120.04-455.03) | 0.58 (0.24-1.00) | -2.53 (-2.69 to -2.38) |
| Fiji | 50.85 (23.01-82.47) | 17.45 (7.74-28.49) | 73.48 (34.43-116.12) | 12.84 (5.96-20.04) | -1.64 (-2.00 to -1.28) |
| Finland | 32.03 (13.61-51.79) | 0.46 (0.20-0.75) | 25.72 (11.02-41.44) | 0.18 (0.08-0.28) | -3.52 (-3.74 to -3.30) |
| France | 413.32 (181.90-642.61) | 0.49 (0.22-0.77) | 252.02 (106.77-404.22) | 0.15 (0.07-0.25) | -4.54 (-4.94 to -4.15) |
| Gabon | 17.48 (6.42-32.65) | 3.25 (1.14-6.38) | 21.50 (8.58-41.67) | 2.27 (0.90-4.60) | -1.31 (-1.43 to -1.19) |
| Gambia | 9.44 (3.84-16.37) | 2.64 (1.09-4.54) | 25.11 (10.24-48.25) | 2.49 (1.01-4.77) | -0.20 (-0.44 to 0.05) |
| Georgia | 120.66 (50.76-190.15) | 2.00 (0.84-3.16) | 25.61 (11.09-41.44) | 0.42 (0.18-0.68) | -4.41 (-5.41 to -3.40) |
| Germany | 1611.20 (695.31-2545.69) | 1.26 (0.55-1.98) | 312.46 (134.26-493.02) | 0.16 (0.07-0.25) | -7.61 (-8.18 to -7.03) |
| Ghana | 76.27 (33.68-130.13) | 1.18 (0.50-2.04) | 238.41 (101.01-407.16) | 1.50 (0.60-2.57) | 1.00 (0.86 to 1.14) |
| Greece | 23.96 (10.87-37.49) | 0.17 (0.08-0.26) | 12.38 (5.67-19.38) | 0.05 (0.02-0.08) | -3.94 (-4.37 to -3.50) |
| Greenland | 0.55 (0.24-0.89) | 1.49 (0.62-2.47) | 0.28 (0.12-0.43) | 0.43 (0.19-0.66) | -4.66 (-4.93 to -4.40) |
| Grenada | 0.51 (0.23-0.80) | 0.73 (0.33-1.15) | 0.74 (0.33-1.15) | 0.67 (0.30-1.05) | -0.47 (-0.84 to -0.10) |
| Guam | 1.04 (0.47-1.63) | 2.04 (0.91-3.26) | 1.14 (0.53-1.82) | 0.54 (0.25-0.86) | -4.00 (-4.27 to -3.73) |
| Guatemala | 78.03 (37.41-120.54) | 2.41 (1.09-3.83) | 54.86 (25.61-86.56) | 0.53 (0.25-0.84) | -5.44 (-5.70 to -5.17) |
| Guinea | 75.40 (33.42-126.14) | 2.29 (0.99-3.91) | 121.85 (50.43-228.43) | 2.12 (0.86-4.01) | 0.05 (-0.14 to 0.23) |
| Guinea-Bissau | 14.65 (6.25-25.70) | 3.55 (1.51-6.32) | 23.69 (10.09-43.74) | 3.02 (1.29-5.59) | -0.28 (-0.48 to -0.09) |
| Guyana | 5.26 (2.23-8.38) | 1.33 (0.55-2.14) | 6.43 (3.02-10.46) | 0.96 (0.45-1.55) | -1.26 (-1.52 to -1.00) |
| Haiti | 50.32 (19.64-104.03) | 1.31 (0.45-3.04) | 113.93 (39.20-292.59) | 1.33 (0.43-3.58) | 0.19 (0.12 to 0.26) |
| Honduras | 34.03 (16.03-57.43) | 1.61 (0.68-2.85) | 70.61 (31.57-123.75) | 1.19 (0.52-2.08) | -1.02 (-1.16 to -0.88) |
| Hungary | 89.44 (38.98-140.88) | 0.63 (0.28-0.99) | 29.58 (13.61-46.30) | 0.15 (0.07-0.23) | -5.36 (-5.77 to -4.95) |
| Iceland | 1.54 (0.70-2.45) | 0.53 (0.24-0.84) | 1.04 (0.48-1.67) | 0.17 (0.08-0.27) | -4.21 (-4.64 to -3.78) |
| India | 6128.35 (2468.45-10634.85) | 1.44 (0.56-2.59) | 21018.30 (8407.39-36835.71) | 1.91 (0.77-3.40) | 1.24 (1.09 to 1.39) |
| Indonesia | 1087.38 (503.44-1760.12) | 1.12 (0.52-1.83) | 3231.94 (1439.41-5316.53) | 1.44 (0.65-2.37) | 0.97 (0.76 to 1.17) |
| Iran (Islamic Republic of) | 462.12 (188.63-738.00) | 2.18 (0.85-3.43) | 967.87 (422.96-1544.40) | 1.41 (0.61-2.25) | -1.65 (-1.84 to -1.46) |
| Iraq | 168.95 (70.90-275.89) | 2.13 (0.87-3.45) | 286.46 (120.84-462.04) | 1.38 (0.58-2.22) | -1.99 (-2.19 to -1.79) |
| Ireland | 29.60 (13.52-46.04) | 0.75 (0.34-1.17) | 13.01 (5.73-20.29) | 0.16 (0.07-0.26) | -5.18 (-5.63 to -4.72) |
| Israel | 52.34 (23.11-82.35) | 1.12 (0.50-1.77) | 34.74 (15.53-53.61) | 0.26 (0.12-0.40) | -5.06 (-5.55 to -4.55) |
| Italy | 376.90 (167.02-600.28) | 0.42 (0.19-0.68) | 115.41 (48.39-184.52) | 0.06 (0.03-0.10) | -6.90 (-7.63 to -6.16) |
| Jamaica | 11.02 (5.09-17.57) | 0.61 (0.28-0.98) | 19.88 (9.42-32.67) | 0.63 (0.30-1.03) | -0.35 (-0.93 to 0.23) |
| Japan | 669.95 (302.72-1063.53) | 0.43 (0.19-0.67) | 247.43 (104.24-405.40) | 0.05 (0.02-0.08) | -8.20 (-8.66 to -7.74) |
| Jordan | 24.52 (11.17-39.10) | 2.14 (0.95-3.42) | 48.71 (23.45-76.24) | 0.83 (0.38-1.28) | -3.90 (-4.37 to -3.43) |
| Kazakhstan | 338.02 (145.60-562.02) | 2.83 (1.23-4.75) | 397.69 (182.37-649.96) | 2.44 (1.13-3.99) | -1.30 (-1.93 to -0.67) |
| Kenya | 89.13 (28.30-261.24) | 1.03 (0.32-3.21) | 357.90 (115.09-1121.77) | 1.58 (0.49-5.24) | 1.74 (1.52 to 1.97) |
| Kiribati | 3.03 (1.15-6.80) | 8.65 (3.03-20.88) | 5.16 (2.23-9.01) | 8.33 (3.39-15.38) | -0.20 (-0.31 to -0.10) |
| Kuwait | 9.48 (4.40-14.23) | 1.92 (0.87-2.92) | 11.23 (5.42-17.40) | 0.49 (0.24-0.78) | -4.74 (-5.69 to -3.79) |
| Kyrgyzstan | 42.52 (18.35-68.58) | 1.46 (0.62-2.38) | 17.34 (7.78-27.26) | 0.38 (0.17-0.60) | -5.48 (-6.01 to -4.94) |
| Lao People's Democratic Republic | 41.73 (18.03-72.97) | 2.13 (0.90-3.79) | 72.64 (27.29-135.35) | 1.69 (0.64-3.27) | -0.91 (-1.02 to -0.81) |
| Latvia | 50.34 (22.98-77.30) | 1.40 (0.64-2.15) | 8.63 (3.93-13.20) | 0.21 (0.10-0.32) | -6.82 (-7.20 to -6.44) |
| Lebanon | 24.84 (9.92-44.45) | 1.30 (0.52-2.31) | 46.96 (21.67-75.44) | 0.72 (0.33-1.15) | -1.78 (-1.90 to -1.65) |
| Lesotho | 31.60 (12.04-64.44) | 3.96 (1.48-8.02) | 61.26 (23.93-107.98) | 5.84 (2.21-10.37) | 1.97 (1.54 to 2.40) |
| Liberia | 37.77 (16.25-63.81) | 3.26 (1.36-5.51) | 59.05 (25.41-106.93) | 2.56 (1.07-4.76) | -0.79 (-1.06 to -0.51) |
| Libya | 53.90 (22.90-90.71) | 3.10 (1.29-5.21) | 143.11 (61.11-242.81) | 3.13 (1.34-5.28) | 0.21 (0.05 to 0.38) |
| Lithuania | 37.16 (16.62-58.67) | 0.83 (0.37-1.30) | 7.92 (3.56-12.52) | 0.13 (0.06-0.21) | -6.65 (-6.98 to -6.32) |
| Luxembourg | 4.65 (2.06-7.17) | 0.88 (0.39-1.35) | 2.52 (1.08-3.98) | 0.22 (0.10-0.35) | -4.89 (-5.30 to -4.47) |
| Madagascar | 89.79 (42.71-148.38) | 1.69 (0.71-2.95) | 246.54 (102.36-473.67) | 2.24 (0.87-4.37) | 0.74 (0.67 to 0.81) |
| Malawi | 54.02 (27.69-85.19) | 1.24 (0.54-2.12) | 116.49 (50.60-213.66) | 1.47 (0.58-2.73) | 0.30 (0.10 to 0.50) |
| Malaysia | 138.63 (58.88-212.53) | 1.47 (0.62-2.26) | 200.46 (83.23-328.54) | 0.77 (0.32-1.28) | -3.21 (-3.70 to -2.71) |
| Maldives | 1.34 (0.56-2.37) | 1.46 (0.61-2.56) | 1.71 (0.76-2.89) | 0.54 (0.23-0.91) | -3.69 (-3.90 to -3.48) |
| Mali | 125.84 (53.45-214.94) | 3.14 (1.28-5.38) | 253.39 (101.79-493.82) | 2.72 (1.12-5.26) | -0.40 (-0.59 to -0.21) |
| Malta | 1.86 (0.82-2.89) | 0.45 (0.20-0.71) | 1.42 (0.62-2.32) | 0.14 (0.06-0.23) | -4.52 (-4.87 to -4.16) |
| Marshall Islands | 1.21 (0.52-1.97) | 7.88 (3.27-12.97) | 1.80 (0.74-3.06) | 6.05 (2.45-10.16) | -0.91 (-1.02 to -0.80) |
| Mauritania | 35.72 (15.57-60.49) | 3.79 (1.65-6.41) | 40.02 (17.12-67.48) | 1.96 (0.83-3.29) | -2.05 (-2.37 to -1.74) |
| Mauritius | 30.37 (13.45-47.57) | 4.77 (2.13-7.55) | 23.53 (10.63-37.13) | 1.38 (0.63-2.17) | -4.29 (-4.62 to -3.95) |
| Mexico | 565.56 (260.02-877.72) | 1.54 (0.70-2.43) | 355.01 (168.28-540.37) | 0.29 (0.14-0.45) | -5.70 (-5.88 to -5.51) |
| Micronesia (Federated States of) | 4.13 (1.81-7.11) | 8.85 (3.64-16.07) | 3.71 (1.66-6.12) | 5.97 (2.58-9.89) | -1.42 (-1.50 to -1.35) |
| Monaco | 0.03 (0.01-0.05) | 0.05 (0.02-0.08) | 0.04 (0.02-0.06) | 0.04 (0.02-0.07) | -0.55 (-0.65 to -0.45) |
| Mongolia | 21.67 (8.23-38.08) | 2.16 (0.81-3.85) | 13.22 (5.86-20.99) | 0.64 (0.28-1.01) | -4.88 (-5.26 to -4.50) |
| Montenegro | 0.87 (0.38-1.46) | 0.15 (0.06-0.25) | 0.99 (0.43-1.56) | 0.11 (0.05-0.17) | -1.45 (-1.84 to -1.05) |
| Morocco | 404.70 (164.74-691.23) | 3.09 (1.24-5.26) | 810.54 (334.52-1350.10) | 2.67 (1.12-4.48) | -0.46 (-0.50 to -0.42) |
| Mozambique | 65.05 (35.90-103.99) | 0.92 (0.42-1.60) | 164.54 (67.86-306.58) | 1.31 (0.52-2.53) | 1.53 (1.38 to 1.67) |
| Myanmar | 710.96 (315.57-1212.91) | 3.22 (1.42-5.48) | 1121.75 (484.87-1993.90) | 2.50 (1.08-4.47) | -1.12 (-1.25 to -0.99) |
| Namibia | 24.61 (9.84-47.37) | 4.26 (1.66-8.66) | 52.15 (22.81-94.78) | 4.18 (1.83-7.44) | -0.25 (-0.56 to 0.07) |
| Nauru | 0.37 (0.15-0.60) | 7.76 (3.12-12.88) | 0.38 (0.17-0.62) | 7.37 (2.94-13.81) | -0.24 (-0.64 to 0.17) |
| Nepal | 208.39 (87.15-383.06) | 2.15 (0.85-3.96) | 528.58 (216.55-1002.89) | 2.29 (0.93-4.41) | 0.46 (0.19 to 0.73) |
| Netherlands | 48.76 (22.26-76.60) | 0.25 (0.11-0.39) | 37.30 (15.80-59.43) | 0.10 (0.04-0.16) | -2.63 (-3.38 to -1.86) |
| New Zealand | 38.48 (17.44-59.33) | 1.02 (0.46-1.57) | 35.50 (16.39-53.79) | 0.45 (0.22-0.68) | -3.07 (-4.04 to -2.09) |
| Nicaragua | 16.47 (7.62-25.36) | 1.05 (0.46-1.68) | 22.40 (9.83-35.89) | 0.49 (0.22-0.79) | -2.85 (-3.17 to -2.53) |
| Niger | 60.12 (26.17-101.62) | 2.14 (0.90-3.78) | 125.37 (48.62-242.58) | 1.54 (0.59-3.16) | -0.98 (-1.26 to -0.70) |
| Nigeria | 627.98 (276.28-1025.85) | 1.54 (0.69-2.54) | 1216.48 (544.44-2003.46) | 1.44 (0.66-2.37) | -0.28 (-0.36 to -0.20) |
| Niue | 0.11 (0.05-0.19) | 4.90 (2.11-8.01) | 0.07 (0.03-0.12) | 3.63 (1.65-5.99) | -1.33 (-1.45 to -1.22) |
| North Macedonia | 32.08 (13.28-52.49) | 1.91 (0.79-3.12) | 18.63 (7.93-32.18) | 0.68 (0.29-1.16) | -4.34 (-4.71 to -3.98) |
| Northern Mariana Islands | 0.62 (0.27-1.05) | 3.92 (1.67-6.62) | 0.83 (0.40-1.25) | 2.18 (1.04-3.41) | -2.31 (-2.55 to -2.07) |
| Norway | 75.61 (32.84-120.45) | 1.06 (0.46-1.68) | 20.69 (8.87-32.65) | 0.19 (0.08-0.29) | -6.41 (-6.99 to -5.83) |
| Oman | 12.89 (5.29-22.17) | 2.07 (0.84-3.56) | 21.08 (9.68-34.51) | 1.40 (0.61-2.37) | -0.77 (-1.01 to -0.53) |
| Pakistan | 918.00 (360.45-1784.05) | 1.71 (0.66-3.39) | 2326.14 (963.84-4921.57) | 2.09 (0.84-4.71) | 0.54 (0.22 to 0.86) |
| Palau | 0.73 (0.34-1.16) | 8.19 (3.77-13.43) | 1.13 (0.52-1.76) | 6.63 (3.04-10.28) | -0.57 (-0.64 to -0.50) |
| Palestine | 19.13 (8.20-32.21) | 2.55 (1.05-4.28) | 26.08 (12.43-39.99) | 1.36 (0.63-2.10) | -2.20 (-2.61 to -1.79) |
| Panama | 11.39 (5.20-18.05) | 0.79 (0.36-1.26) | 14.94 (6.97-23.80) | 0.33 (0.15-0.52) | -3.39 (-3.88 to -2.89) |
| Papua New Guinea | 93.47 (35.75-167.66) | 5.82 (2.14-10.59) | 268.02 (111.78-538.38) | 5.85 (2.36-12.05) | 0.05 (-0.01 to 0.11) |
| Paraguay | 10.26 (4.69-16.51) | 0.46 (0.21-0.75) | 23.44 (10.90-38.54) | 0.41 (0.19-0.68) | -0.31 (-0.45 to -0.17) |
| Peru | 61.14 (32.24-94.53) | 0.43 (0.21-0.69) | 59.13 (24.24-103.87) | 0.18 (0.07-0.31) | -3.23 (-3.48 to -2.99) |
| Philippines | 509.26 (217.79-821.49) | 1.93 (0.78-3.11) | 1450.22 (635.00-2362.64) | 1.85 (0.81-3.03) | -0.07 (-0.32 to 0.18) |
| Poland | 722.81 (326.62-1126.39) | 1.72 (0.78-2.68) | 171.95 (79.19-270.47) | 0.23 (0.11-0.36) | -7.34 (-8.12 to -6.55) |
| Portugal | 97.90 (43.94-152.67) | 0.76 (0.34-1.20) | 43.75 (19.16-69.91) | 0.16 (0.07-0.26) | -5.20 (-5.84 to -4.56) |
| Puerto Rico | 43.14 (19.94-66.41) | 1.23 (0.57-1.89) | 33.27 (15.81-51.87) | 0.51 (0.24-0.80) | -3.94 (-4.55 to -3.33) |
| Qatar | 2.17 (0.90-3.39) | 3.13 (1.26-5.19) | 5.08 (2.46-8.16) | 0.97 (0.44-1.56) | -4.56 (-5.38 to -3.73) |
| Republic of Korea | 671.88 (281.20-1119.82) | 3.76 (1.49-6.37) | 349.11 (143.27-616.89) | 0.40 (0.17-0.70) | -8.56 (-9.07 to -8.04) |
| Republic of Moldova | 45.32 (19.68-71.26) | 1.06 (0.46-1.65) | 7.43 (3.51-11.19) | 0.13 (0.06-0.19) | -7.87 (-8.33 to -7.40) |
| Romania | 287.79 (125.50-458.66) | 1.12 (0.48-1.78) | 66.35 (29.44-104.73) | 0.17 (0.08-0.27) | -6.33 (-6.68 to -5.97) |
| Russian Federation | 2219.94 (987.57-3440.98) | 1.24 (0.55-1.92) | 408.83 (185.29-617.53) | 0.17 (0.08-0.26) | -7.71 (-8.16 to -7.25) |
| Rwanda | 62.92 (30.17-104.41) | 1.87 (0.83-3.32) | 74.86 (28.94-164.15) | 1.15 (0.43-2.66) | -2.57 (-2.94 to -2.20) |
| Saint Kitts and Nevis | 0.33 (0.14-0.52) | 0.91 (0.39-1.44) | 0.33 (0.15-0.54) | 0.51 (0.24-0.81) | -2.08 (-2.57 to -1.59) |
| Saint Lucia | 1.24 (0.56-1.94) | 1.44 (0.64-2.25) | 1.90 (0.89-3.01) | 0.82 (0.39-1.31) | -2.51 (-2.92 to -2.10) |
| Saint Vincent and the Grenadines | 0.64 (0.29-1.00) | 0.90 (0.41-1.42) | 0.95 (0.45-1.51) | 0.70 (0.33-1.12) | -1.01 (-1.54 to -0.48) |
| Samoa | 5.63 (2.31-11.17) | 7.26 (2.89-15.32) | 6.75 (3.15-11.43) | 5.17 (2.38-8.83) | -1.08 (-1.16 to -1.00) |
| San Marino | 0.07 (0.03-0.11) | 0.18 (0.08-0.29) | 0.08 (0.03-0.13) | 0.09 (0.04-0.15) | -1.52 (-1.90 to -1.14) |
| Sao Tome and Principe | 3.48 (1.51-5.84) | 5.65 (2.44-9.65) | 5.42 (2.25-9.22) | 5.03 (2.14-8.76) | -0.47 (-0.59 to -0.35) |
| Saudi Arabia | 203.18 (85.68-338.03) | 3.85 (1.58-6.41) | 425.21 (201.81-686.54) | 2.57 (1.25-4.10) | -1.48 (-1.63 to -1.34) |
| Senegal | 80.65 (35.08-132.57) | 2.48 (1.04-4.08) | 148.68 (64.63-257.03) | 1.95 (0.84-3.42) | -0.48 (-0.89 to -0.07) |
| Serbia | 138.61 (58.86-240.12) | 1.52 (0.63-2.67) | 98.19 (43.88-156.06) | 0.57 (0.26-0.91) | -4.03 (-4.42 to -3.64) |
| Seychelles | 1.08 (0.47-1.68) | 1.88 (0.83-2.96) | 1.52 (0.67-2.46) | 1.44 (0.63-2.35) | -0.86 (-0.99 to -0.74) |
| Sierra Leone | 42.76 (18.77-72.51) | 2.09 (0.88-3.64) | 72.16 (30.21-136.59) | 1.86 (0.77-3.61) | -0.11 (-0.29 to 0.08) |
| Singapore | 13.56 (6.30-21.31) | 0.65 (0.30-1.03) | 4.71 (2.02-7.56) | 0.06 (0.02-0.09) | -8.77 (-9.22 to -8.30) |
| Slovakia | 31.08 (13.53-48.16) | 0.53 (0.23-0.81) | 14.21 (6.31-22.48) | 0.15 (0.07-0.23) | -4.63 (-4.94 to -4.31) |
| Slovenia | 16.22 (7.19-24.61) | 0.66 (0.29-1.00) | 5.41 (2.43-8.42) | 0.10 (0.05-0.16) | -6.79 (-7.41 to -6.17) |
| Solomon Islands | 4.57 (1.83-8.27) | 3.83 (1.58-6.92) | 11.65 (4.96-20.73) | 3.73 (1.58-6.67) | -0.02 (-0.08 to 0.04) |
| Somalia | 54.55 (23.22-99.26) | 1.83 (0.73-3.32) | 137.36 (55.95-251.37) | 1.85 (0.70-3.49) | -0.05 (-0.14 to 0.04) |
| South Africa | 828.04 (351.80-1347.10) | 3.86 (1.61-6.52) | 1550.77 (722.03-2360.64) | 3.56 (1.64-5.46) | -0.48 (-1.08 to 0.12) |
| South Sudan | 31.90 (15.61-53.31) | 1.08 (0.45-1.92) | 45.89 (20.70-85.93) | 1.03 (0.42-1.98) | -0.36 (-0.72 to 0.01) |
| Spain | 290.06 (130.46-454.15) | 0.55 (0.25-0.86) | 312.54 (134.28-488.46) | 0.25 (0.11-0.38) | -2.54 (-2.84 to -2.23) |
| Sri Lanka | 209.63 (86.19-339.84) | 2.16 (0.88-3.53) | 302.01 (119.21-548.88) | 1.23 (0.49-2.25) | -1.85 (-2.04 to -1.66) |
| Sudan | 427.57 (145.10-1059.03) | 5.05 (1.66-13.42) | 566.08 (228.38-1079.14) | 3.26 (1.26-6.14) | -1.70 (-1.80 to -1.60) |
| Suriname | 1.88 (0.82-3.02) | 0.70 (0.31-1.13) | 3.41 (1.51-5.54) | 0.54 (0.24-0.88) | -1.11 (-1.32 to -0.91) |
| Sweden | 92.96 (41.51-146.58) | 0.61 (0.27-0.97) | 34.48 (14.74-55.69) | 0.14 (0.06-0.22) | -4.52 (-5.07 to -3.96) |
| Switzerland | 67.88 (29.41-105.64) | 0.64 (0.28-0.99) | 25.66 (11.11-41.52) | 0.12 (0.05-0.20) | -5.79 (-6.32 to -5.25) |
| Syrian Arab Republic | 171.92 (74.67-273.87) | 3.52 (1.48-5.81) | 357.07 (170.93-577.35) | 3.30 (1.50-5.37) | -0.49 (-0.66 to -0.32) |
| Taiwan (Province of China) | 212.59 (92.52-335.95) | 1.70 (0.75-2.72) | 135.95 (56.31-217.56) | 0.31 (0.13-0.50) | -6.07 (-6.50 to -5.62) |
| Tajikistan | 77.19 (32.17-135.39) | 3.09 (1.28-5.51) | 77.03 (33.12-132.61) | 1.69 (0.72-2.90) | -2.21 (-2.54 to -1.89) |
| Thailand | 325.08 (135.94-530.95) | 1.01 (0.43-1.67) | 685.78 (303.05-1150.27) | 0.66 (0.29-1.10) | -1.96 (-2.17 to -1.76) |
| Timor-Leste | 2.59 (1.29-4.47) | 0.87 (0.39-1.54) | 7.28 (3.22-12.52) | 0.87 (0.37-1.52) | 0.03 (-0.21 to 0.27) |
| Togo | 30.02 (13.67-51.76) | 2.40 (1.03-4.18) | 85.92 (34.45-159.17) | 2.33 (0.88-4.45) | 0.11 (-0.04 to 0.26) |
| Tokelau | 0.06 (0.02-0.12) | 5.09 (1.93-9.58) | 0.05 (0.02-0.09) | 3.58 (1.61-6.28) | -1.42 (-1.53 to -1.32) |
| Tonga | 2.20 (0.99-3.69) | 4.31 (1.87-7.33) | 2.89 (1.41-4.74) | 3.72 (1.81-6.14) | -0.37 (-0.50 to -0.23) |
| Trinidad and Tobago | 12.51 (5.57-19.37) | 1.53 (0.67-2.37) | 15.48 (7.08-24.69) | 0.84 (0.38-1.33) | -2.48 (-2.81 to -2.16) |
| Tunisia | 99.09 (43.03-159.77) | 2.33 (0.98-3.81) | 217.94 (91.06-377.45) | 1.83 (0.78-3.25) | -1.05 (-1.16 to -0.95) |
| Turkey | 563.10 (239.14-914.30) | 1.86 (0.76-3.09) | 844.91 (383.99-1365.77) | 1.01 (0.45-1.64) | -1.89 (-2.18 to -1.60) |
| Turkmenistan | 31.69 (12.97-53.78) | 1.72 (0.71-2.94) | 16.66 (6.60-29.59) | 0.40 (0.16-0.71) | -5.75 (-6.45 to -5.06) |
| Tuvalu | 0.44 (0.18-0.72) | 7.32 (3.06-12.05) | 0.44 (0.19-0.74) | 4.87 (2.10-8.37) | -1.34 (-1.41 to -1.27) |
| Uganda | 84.11 (38.01-153.73) | 1.15 (0.47-2.21) | 190.62 (71.22-433.58) | 1.15 (0.41-2.78) | -0.57 (-0.79 to -0.34) |
| Ukraine | 218.46 (93.33-350.46) | 0.31 (0.13-0.49) | 36.62 (14.97-59.82) | 0.05 (0.02-0.08) | -7.52 (-8.15 to -6.87) |
| United Arab Emirates | 18.26 (7.29-31.60) | 3.88 (1.51-6.66) | 63.33 (29.83-98.57) | 2.62 (1.12-4.12) | 0.30 (-0.33 to 0.94) |
| United Kingdom | 537.23 (241.34-841.91) | 0.64 (0.29-0.99) | 478.62 (213.17-746.00) | 0.36 (0.16-0.55) | -1.70 (-2.17 to -1.23) |
| United Republic of Tanzania | 137.57 (58.63-236.91) | 1.23 (0.49-2.13) | 344.50 (135.84-695.04) | 1.32 (0.50-2.74) | 0.15 (0.09 to 0.21) |
| United States Virgin Islands | 0.99 (0.45-1.54) | 1.21 (0.54-1.87) | 0.85 (0.37-1.52) | 0.56 (0.25-0.98) | -2.33 (-2.51 to -2.14) |
| United States of America | 1276.66 (575.90-1980.64) | 0.42 (0.19-0.64) | 1226.90 (581.29-1841.42) | 0.26 (0.13-0.38) | -2.21 (-2.52 to -1.89) |
| Uruguay | 35.05 (15.61-55.50) | 0.92 (0.41-1.45) | 30.86 (14.14-47.95) | 0.54 (0.25-0.83) | -2.09 (-2.38 to -1.80) |
| Uzbekistan | 447.93 (191.53-737.74) | 4.02 (1.72-6.68) | 263.54 (113.09-429.95) | 1.11 (0.49-1.77) | -5.44 (-6.33 to -4.53) |
| Vanuatu | 3.00 (1.08-6.77) | 5.32 (1.87-12.40) | 7.91 (3.06-16.84) | 5.16 (1.91-12.07) | -0.29 (-0.39 to -0.18) |
| Venezuela (Bolivarian Republic of) | 77.58 (35.86-121.06) | 0.80 (0.36-1.26) | 107.07 (51.03-171.36) | 0.37 (0.18-0.59) | -3.32 (-3.77 to -2.88) |
| Viet Nam | 213.37 (91.68-377.72) | 0.57 (0.24-1.02) | 511.00 (209.99-884.48) | 0.58 (0.23-1.01) | 0.27 (0.16 to 0.38) |
| Yemen | 138.30 (54.76-259.96) | 3.13 (1.12-6.53) | 370.54 (139.00-644.89) | 3.06 (1.12-5.33) | -0.36 (-0.56 to -0.16) |
| Zambia | 44.96 (23.11-69.27) | 1.30 (0.57-2.16) | 130.65 (54.83-243.49) | 1.67 (0.67-3.10) | 0.63 (0.48 to 0.79) |
| Zimbabwe | 94.29 (38.78-155.81) | 2.50 (1.02-4.21) | 337.28 (135.81-585.45) | 5.08 (2.04-8.76) | 2.97 (2.56 to 3.38) |

Abbreviations: ASMR = age-standardized mortality rate; UI = uncertainty interval; CI = confidence interval; EAPC = estimated annual percentage change.
