# Supplementary material for: The influence of high body mass index on asthma: A study integrating GBD 2021 and Mendelian randomization
Source: Medicine (Baltimore). 2026 Nov 6;105(45):e51490. doi: 10.1097/MD.0000000000051490 (PMC13747491; doi:10.1097/MD.0000000000051490)
Supplement: Supplementary file 3 [file medi-105-e51490-s003.docx]

**Table S3.**  Number of asthma DALYs attributable to high BMI across 204 countries and territories, with EAPC (1990–2021).

| **location** | **1990** | | **2021** | | **1990–2021** |
| --- | --- | --- | --- | --- | --- |
|  | **DALYs cases（95%UI）** | **ASDR per 100,000(95%UI)** | **DALYs cases（95%UI）** | **ASDR per 100,000(95%UI)** | **EAPC(95%CI)** |
| Afghanistan | 15216.26 (6757.06-25838.63) | 208.03 (90.08-358.95) | 19561.88 (9122.73-33699.36) | 149.93 (64.67-270.46) | -1.40 (-1.62 to -1.19) |
| Albania | 1578.89 (799.55-2395.92) | 73.71 (35.12-113.44) | 1258.85 (627.67-1968.76) | 35.08 (18.61-54.39) | -2.56 (-2.76 to -2.36) |
| Algeria | 10480.03 (5151.42-16375.57) | 77.98 (34.65-123.99) | 24081.62 (12464.03-36948.08) | 65.08 (33.54-101.75) | -0.66 (-0.71 to -0.60) |
| American Samoa | 48.84 (25.94-71.93) | 163.67 (80.45-244.63) | 42.23 (23.05-60.90) | 89.38 (48.38-129.84) | -2.29 (-2.46 to -2.11) |
| Andorra | 43.09 (19.20-71.43) | 72.00 (33.68-116.49) | 54.17 (22.73-90.90) | 52.30 (24.23-85.58) | -1.19 (-1.36 to -1.02) |
| Angola | 4276.23 (2392.23-6717.16) | 64.60 (31.70-117.99) | 10862.09 (5933.48-16689.50) | 54.06 (24.18-92.62) | -0.92 (-1.05 to -0.79) |
| Antigua and Barbuda | 25.13 (13.26-38.61) | 43.71 (22.99-66.57) | 47.63 (25.40-72.00) | 52.38 (29.29-77.43) | 0.43 (0.31 to 0.54) |
| Argentina | 22588.33 (10635.94-35965.99) | 69.45 (32.55-111.03) | 29376.63 (13737.58-46579.55) | 59.57 (28.23-93.30) | -0.79 (-0.89 to -0.68) |
| Armenia | 611.64 (301.85-951.21) | 19.70 (9.60-30.56) | 431.35 (213.22-660.06) | 13.25 (6.75-20.48) | -1.19 (-1.48 to -0.89) |
| Australia | 18343.69 (8906.37-28826.41) | 101.61 (50.05-159.05) | 21206.37 (10088.56-33596.85) | 75.63 (37.39-119.84) | -1.28 (-1.51 to -1.04) |
| Austria | 6738.48 (3066.68-10952.28) | 68.65 (31.39-111.86) | 3913.67 (1731.79-6314.25) | 35.87 (16.73-57.56) | -2.21 (-2.37 to -2.05) |
| Azerbaijan | 4015.26 (1938.05-6136.36) | 73.71 (35.15-113.86) | 4292.95 (2118.79-6842.85) | 40.22 (20.33-63.31) | -2.55 (-2.74 to -2.36) |
| Bahamas | 132.03 (68.41-198.90) | 57.21 (29.53-86.85) | 240.02 (127.14-359.79) | 61.58 (34.22-91.84) | 0.02 (-0.09 to 0.13) |
| Bahrain | 293.16 (150.21-451.04) | 118.46 (58.34-180.20) | 748.58 (383.54-1128.57) | 68.80 (35.52-103.34) | -2.38 (-2.69 to -2.07) |
| Bangladesh | 20716.13 (9654.70-34373.30) | 35.18 (15.45-61.05) | 45403.68 (19089.73-81043.74) | 30.21 (12.50-54.52) | -0.32 (-0.43 to -0.21) |
| Barbados | 191.55 (97.46-303.17) | 73.22 (37.58-114.96) | 285.81 (142.40-432.72) | 83.63 (44.84-127.16) | 0.28 (0.12 to 0.44) |
| Belarus | 8118.49 (3646.02-13279.20) | 66.00 (30.18-107.83) | 4062.93 (1788.73-6723.46) | 33.98 (15.79-55.25) | -2.65 (-2.85 to -2.45) |
| Belgium | 8598.90 (3844.81-14161.02) | 68.86 (31.60-113.50) | 4971.80 (2228.81-8102.35) | 37.09 (17.19-60.48) | -2.09 (-2.33 to -1.84) |
| Belize | 92.05 (50.02-136.52) | 57.58 (30.17-87.78) | 286.97 (151.94-426.30) | 68.01 (35.81-101.11) | 0.18 (-0.14 to 0.49) |
| Benin | 2417.00 (1257.69-3846.36) | 91.44 (43.49-151.82) | 5183.66 (2598.86-8580.58) | 65.94 (30.03-115.11) | -1.01 (-1.21 to -0.82) |
| Bermuda | 34.33 (17.16-52.46) | 56.49 (29.80-84.75) | 33.78 (17.93-50.16) | 57.40 (31.98-88.06) | -0.12 (-0.21 to -0.02) |
| Bhutan | 273.53 (123.90-479.36) | 88.12 (36.46-158.71) | 376.33 (163.61-728.51) | 58.44 (25.34-114.18) | -1.57 (-1.68 to -1.47) |
| Bolivia (Plurinational State of) | 3316.67 (1970.54-5139.94) | 49.55 (29.22-76.44) | 3432.42 (2002.96-5094.55) | 30.46 (17.93-45.31) | -1.70 (-1.81 to -1.60) |
| Bosnia and Herzegovina | 1959.72 (930.34-3099.71) | 45.34 (21.84-71.66) | 1741.93 (824.02-2827.90) | 41.36 (20.66-65.38) | -0.39 (-0.45 to -0.34) |
| Botswana | 790.00 (353.32-1495.19) | 115.75 (47.52-226.13) | 1706.86 (759.14-2820.32) | 96.12 (42.54-161.15) | -0.52 (-0.71 to -0.33) |
| Brazil | 56505.97 (31208.39-85428.17) | 42.88 (22.21-66.03) | 81119.25 (42728.21-122758.05) | 37.35 (20.69-56.22) | -0.96 (-1.15 to -0.76) |
| Brunei Darussalam | 95.49 (45.34-151.32) | 61.61 (29.02-95.97) | 185.13 (81.61-293.14) | 45.99 (21.03-72.02) | -0.89 (-0.97 to -0.80) |
| Bulgaria | 5331.55 (2476.73-8548.57) | 48.62 (23.00-77.91) | 2168.30 (997.42-3545.16) | 26.76 (13.11-42.00) | -2.31 (-2.46 to -2.15) |
| Burkina Faso | 2600.32 (1514.32-3964.42) | 37.40 (19.73-59.00) | 5546.41 (3201.76-8285.80) | 33.22 (17.14-50.48) | -0.40 (-0.55 to -0.26) |
| Burundi | 1905.49 (1069.79-2942.67) | 49.07 (25.30-80.80) | 3153.36 (1765.50-5178.47) | 36.36 (17.01-64.93) | -1.38 (-1.60 to -1.15) |
| Cabo Verde | 174.75 (78.53-284.25) | 75.30 (32.19-123.37) | 191.12 (90.13-308.61) | 37.88 (17.58-61.91) | -2.05 (-2.57 to -1.53) |
| Cambodia | 1737.25 (886.56-2686.07) | 30.32 (13.69-48.31) | 3716.98 (1739.65-6284.41) | 27.17 (12.72-46.51) | -0.48 (-0.54 to -0.43) |
| Cameroon | 6672.12 (3464.24-10659.07) | 116.62 (55.44-193.28) | 15735.54 (8191.20-25102.13) | 85.90 (39.91-143.27) | -0.90 (-0.98 to -0.82) |
| Canada | 18405.26 (8470.68-29494.47) | 62.45 (30.07-98.33) | 19488.30 (9379.70-30792.65) | 48.25 (25.06-75.34) | -0.54 (-0.71 to -0.37) |
| Central African Republic | 1329.72 (635.26-2325.21) | 78.66 (33.61-159.64) | 3577.61 (1665.72-7420.31) | 105.12 (44.09-254.44) | 0.84 (0.78 to 0.90) |
| Chad | 2027.26 (982.52-3264.26) | 58.93 (25.68-97.67) | 4886.13 (2380.86-7841.67) | 56.30 (24.54-97.19) | -0.11 (-0.17 to -0.04) |
| Chile | 8389.51 (4069.22-13281.23) | 73.53 (35.26-117.27) | 14816.62 (7046.35-23537.47) | 68.64 (33.68-106.83) | -0.32 (-0.47 to -0.18) |
| China | 150558.19 (72480.56-238271.02) | 16.50 (7.76-25.92) | 218662.32 (107327.86-339079.19) | 13.47 (6.91-20.77) | -0.95 (-1.16 to -0.74) |
| Colombia | 9701.26 (5014.48-14729.62) | 39.54 (19.95-61.14) | 11403.21 (5787.10-17921.64) | 22.95 (12.07-35.65) | -2.01 (-2.22 to -1.79) |
| Comoros | 236.23 (127.81-368.48) | 68.98 (33.17-112.97) | 455.65 (255.40-708.96) | 71.07 (37.77-115.11) | -0.22 (-0.44 to -0.01) |
| Congo | 1343.57 (602.97-2438.89) | 97.34 (39.69-187.98) | 3106.75 (1588.25-5061.92) | 81.82 (39.41-138.83) | -0.92 (-1.10 to -0.73) |
| Cook Islands | 16.24 (8.74-25.12) | 111.15 (57.05-174.01) | 11.22 (5.97-16.76) | 54.25 (29.32-79.14) | -2.34 (-2.46 to -2.21) |
| Costa Rica | 1640.56 (855.21-2552.23) | 62.74 (31.74-98.87) | 2530.07 (1254.23-3945.87) | 52.36 (27.72-81.00) | -1.00 (-1.20 to -0.80) |
| Croatia | 3817.19 (1767.42-6119.98) | 66.17 (32.14-105.12) | 1985.24 (930.56-3186.38) | 35.23 (17.95-55.06) | -2.31 (-2.56 to -2.06) |
| Cuba | 6333.30 (3140.57-9804.07) | 57.87 (28.95-88.83) | 7215.49 (3617.81-10805.13) | 62.37 (34.30-93.77) | 0.01 (-0.10 to 0.13) |
| Cyprus | 590.21 (265.20-977.75) | 75.57 (33.73-124.94) | 1147.59 (490.32-1905.08) | 70.29 (31.91-115.41) | -0.29 (-0.35 to -0.23) |
| Czechia | 4437.57 (2142.08-6812.82) | 36.67 (18.02-56.11) | 3016.77 (1468.79-4747.19) | 23.23 (11.93-36.63) | -1.39 (-1.62 to -1.17) |
| Coate d'Ivoire | 5015.52 (2426.85-7935.03) | 84.49 (38.39-138.18) | 11589.76 (5781.21-18970.18) | 69.03 (31.85-114.47) | -0.57 (-0.64 to -0.50) |
| Democratic People's Republic of Korea | 4605.61 (2333.95-7852.45) | 27.53 (13.66-47.72) | 8926.45 (4218.89-15286.74) | 31.41 (15.69-53.33) | 0.43 (0.36 to 0.49) |
| Democratic Republic of the Congo | 11644.80 (5635.90-22667.10) | 56.05 (22.37-128.43) | 38905.87 (17421.69-84893.49) | 80.87 (30.99-203.61) | 1.20 (1.15 to 1.25) |
| Denmark | 3848.69 (1764.97-6189.36) | 60.65 (29.07-96.42) | 2534.11 (1151.23-4111.92) | 38.27 (18.08-61.66) | -1.71 (-1.89 to -1.53) |
| Djibouti | 102.85 (57.42-155.12) | 33.85 (18.32-53.68) | 312.34 (164.37-486.64) | 30.22 (14.79-48.65) | -0.62 (-0.71 to -0.53) |
| Dominica | 51.42 (27.57-78.00) | 79.22 (41.52-120.65) | 67.35 (36.40-98.57) | 92.80 (51.14-134.75) | 0.36 (0.24 to 0.48) |
| Dominican Republic | 3056.99 (1654.68-4413.32) | 55.31 (29.69-83.09) | 5183.92 (2746.59-7909.57) | 47.64 (25.30-72.25) | -0.21 (-0.33 to -0.10) |
| Ecuador | 3437.03 (2020.58-5096.29) | 37.82 (21.74-55.41) | 3981.85 (2151.69-6074.96) | 22.21 (12.05-33.82) | -1.62 (-1.99 to -1.25) |
| Egypt | 50597.58 (26614.76-74144.68) | 152.03 (71.46-230.46) | 62279.56 (34108.66-90858.97) | 82.54 (43.40-119.92) | -2.24 (-2.44 to -2.03) |
| El Salvador | 3607.49 (1890.70-5444.55) | 87.86 (45.32-133.05) | 3912.87 (2007.37-5977.75) | 61.20 (31.52-93.82) | -1.55 (-1.71 to -1.40) |
| Equatorial Guinea | 309.07 (159.73-523.37) | 110.00 (50.29-208.21) | 612.76 (335.87-976.73) | 64.19 (31.26-114.30) | -2.30 (-2.52 to -2.07) |
| Eritrea | 760.87 (414.88-1158.99) | 36.34 (17.98-58.01) | 1683.91 (873.06-2604.06) | 37.90 (17.33-60.40) | 0.09 (-0.00 to 0.18) |
| Estonia | 1113.40 (523.07-1713.39) | 57.53 (27.28-89.13) | 422.06 (195.62-665.88) | 24.61 (11.77-38.33) | -3.23 (-3.48 to -2.99) |
| Eswatini | 828.52 (395.49-1419.20) | 208.98 (88.93-374.34) | 1627.37 (816.55-2706.28) | 219.03 (104.04-365.39) | 0.43 (-0.09 to 0.95) |
| Ethiopia | 12863.20 (6759.13-19882.98) | 40.90 (19.72-65.77) | 14947.98 (8155.35-22825.41) | 21.25 (10.15-33.34) | -2.58 (-2.74 to -2.43) |
| Fiji | 1708.09 (799.64-2681.35) | 412.30 (188.16-658.93) | 2128.43 (1050.32-3304.40) | 285.03 (138.37-435.79) | -1.70 (-1.96 to -1.43) |
| Finland | 3658.67 (1646.88-5993.14) | 61.01 (27.78-98.93) | 3751.35 (1691.73-6194.29) | 53.66 (25.34-88.56) | -0.38 (-0.43 to -0.34) |
| France | 46791.59 (20457.09-78177.46) | 68.24 (30.29-115.39) | 38171.93 (16748.22-63566.66) | 48.64 (22.37-80.64) | -1.21 (-1.35 to -1.08) |
| Gabon | 623.85 (265.16-1027.01) | 97.44 (38.99-166.34) | 934.56 (464.68-1562.84) | 71.89 (34.42-124.23) | -1.15 (-1.27 to -1.03) |
| Gambia | 388.04 (178.31-653.27) | 82.11 (36.00-137.29) | 1046.33 (484.51-1868.57) | 78.90 (34.60-145.59) | -0.20 (-0.43 to 0.03) |
| Georgia | 3943.86 (1761.03-6096.77) | 63.84 (28.65-98.45) | 1028.84 (512.27-1605.42) | 22.43 (11.68-34.17) | -3.15 (-3.63 to -2.67) |
| Germany | 99245.79 (45967.44-160101.71) | 93.58 (45.21-150.66) | 37989.83 (17591.11-62954.85) | 37.12 (17.65-60.60) | -2.64 (-2.98 to -2.31) |
| Ghana | 3585.74 (1743.66-5693.71) | 40.31 (18.64-66.30) | 10641.95 (4942.85-16980.61) | 46.98 (20.87-77.37) | 0.62 (0.50 to 0.73) |
| Greece | 6169.52 (2749.14-10227.96) | 49.40 (22.48-81.05) | 4963.89 (2210.11-8093.27) | 41.91 (19.23-68.74) | -0.74 (-0.86 to -0.63) |
| Greenland | 68.89 (34.70-108.89) | 143.69 (72.57-225.59) | 50.15 (24.30-79.51) | 80.51 (40.40-125.05) | -2.31 (-2.45 to -2.17) |
| Grenada | 49.33 (25.06-76.44) | 65.33 (32.14-102.76) | 76.40 (38.22-115.51) | 72.07 (38.04-107.67) | 0.26 (0.15 to 0.36) |
| Guam | 61.24 (32.48-90.68) | 63.38 (33.77-94.50) | 51.11 (28.02-75.53) | 29.84 (16.62-43.97) | -2.38 (-2.56 to -2.21) |
| Guatemala | 4741.27 (2734.43-6936.69) | 82.42 (43.19-122.99) | 4164.98 (2267.43-6158.80) | 29.43 (15.37-43.38) | -3.90 (-4.20 to -3.60) |
| Guinea | 2991.21 (1496.74-4746.45) | 74.35 (34.87-121.09) | 5380.43 (2610.83-9227.75) | 68.62 (31.31-121.85) | -0.03 (-0.19 to 0.12) |
| Guinea-Bissau | 628.79 (297.40-1050.86) | 116.20 (51.22-198.55) | 1108.24 (507.25-1894.46) | 98.53 (42.92-175.27) | -0.35 (-0.52 to -0.17) |
| Guyana | 358.49 (183.70-541.83) | 63.84 (30.79-99.12) | 504.33 (257.77-772.43) | 66.77 (34.09-102.18) | -0.09 (-0.26 to 0.08) |
| Haiti | 4005.46 (2042.37-6562.94) | 73.67 (34.87-128.18) | 9858.81 (4786.87-17520.80) | 84.21 (41.45-155.31) | 0.53 (0.46 to 0.60) |
| Honduras | 2139.97 (1265.02-3164.94) | 62.52 (31.20-97.61) | 3733.89 (2041.46-5907.37) | 46.52 (24.15-73.81) | -1.10 (-1.18 to -1.02) |
| Hungary | 6046.99 (2782.88-9721.27) | 47.53 (22.49-75.31) | 3308.80 (1563.14-5199.33) | 28.58 (14.11-44.94) | -1.88 (-2.02 to -1.75) |
| Iceland | 254.99 (117.88-416.70) | 97.20 (45.37-157.48) | 252.17 (114.33-410.02) | 66.15 (31.27-107.10) | -1.41 (-1.49 to -1.32) |
| India | 224190.77 (103470.34-361935.91) | 41.27 (18.06-69.02) | 632587.57 (261685.39-1046890.64) | 51.12 (21.10-85.01) | 0.94 (0.84 to 1.03) |
| Indonesia | 48901.21 (23956.11-75418.68) | 37.48 (17.88-58.41) | 125603.90 (56599.51-200199.42) | 46.47 (21.42-74.28) | 0.89 (0.68 to 1.10) |
| Iran (Islamic Republic of) | 22232.47 (10882.12-34351.55) | 69.90 (31.18-108.58) | 41672.89 (21405.41-63168.59) | 51.55 (26.83-77.93) | -1.25 (-1.39 to -1.11) |
| Iraq | 9461.56 (4805.30-14636.09) | 89.17 (42.22-140.13) | 18391.53 (9725.84-27576.70) | 57.83 (29.79-88.25) | -1.81 (-1.98 to -1.65) |
| Ireland | 3839.22 (1804.21-6203.30) | 104.59 (48.83-169.29) | 3288.14 (1521.91-5318.15) | 61.57 (29.38-100.23) | -1.86 (-1.99 to -1.73) |
| Israel | 3063.03 (1500.69-4857.07) | 64.42 (31.27-102.09) | 3289.91 (1548.24-5230.29) | 32.37 (15.19-51.61) | -2.51 (-2.76 to -2.26) |
| Italy | 39964.98 (17434.39-65706.34) | 51.10 (23.17-83.14) | 19540.15 (8485.00-32314.01) | 25.98 (12.25-42.51) | -2.53 (-2.76 to -2.30) |
| Jamaica | 1155.19 (619.65-1764.15) | 53.62 (28.31-82.35) | 1674.33 (875.59-2533.73) | 58.50 (31.02-87.85) | -0.03 (-0.31 to 0.26) |
| Japan | 65932.01 (30581.41-108703.22) | 44.17 (21.59-72.90) | 24587.48 (11301.68-40002.67) | 15.32 (7.90-24.70) | -3.96 (-4.26 to -3.66) |
| Jordan | 1480.37 (816.04-2182.36) | 77.50 (38.03-117.49) | 4566.21 (2440.01-6825.24) | 44.50 (22.93-66.67) | -2.27 (-2.54 to -2.00) |
| Kazakhstan | 10035.99 (4528.53-15942.87) | 76.06 (33.52-121.57) | 11885.70 (5673.48-18767.30) | 65.00 (30.91-102.87) | -1.38 (-1.91 to -0.84) |
| Kenya | 4929.89 (2317.63-10398.66) | 37.04 (15.47-91.27) | 17013.08 (7649.82-40105.91) | 51.99 (20.18-139.86) | 1.37 (1.17 to 1.58) |
| Kiribati | 139.45 (62.83-251.92) | 283.67 (120.13-563.41) | 221.94 (103.81-357.23) | 248.97 (114.48-419.28) | -0.53 (-0.64 to -0.42) |
| Kuwait | 810.57 (428.02-1234.67) | 74.32 (38.51-110.78) | 1999.80 (992.23-3071.52) | 46.83 (25.47-69.72) | -1.65 (-2.02 to -1.29) |
| Kyrgyzstan | 2165.29 (1073.35-3311.69) | 65.13 (30.87-100.99) | 1663.18 (838.21-2555.89) | 27.52 (13.85-42.20) | -3.68 (-4.03 to -3.34) |
| Lao People's Democratic Republic | 1509.06 (725.76-2600.77) | 59.57 (26.93-102.73) | 2588.93 (1099.67-4593.83) | 47.23 (19.39-83.91) | -0.87 (-0.99 to -0.75) |
| Latvia | 2351.43 (1094.89-3667.76) | 69.63 (32.33-108.71) | 657.33 (307.45-1030.80) | 26.15 (12.75-41.12) | -3.58 (-3.85 to -3.32) |
| Lebanon | 1625.11 (802.19-2570.02) | 66.75 (31.25-107.20) | 3166.44 (1645.85-4877.72) | 54.73 (28.68-83.12) | -0.65 (-0.71 to -0.59) |
| Lesotho | 959.58 (404.53-1857.54) | 103.21 (42.15-204.25) | 2024.36 (818.73-3556.20) | 160.99 (64.47-283.45) | 2.19 (1.74 to 2.65) |
| Liberia | 1487.37 (738.70-2400.49) | 101.93 (46.61-168.13) | 2720.94 (1344.85-4534.32) | 82.29 (38.38-143.10) | -0.75 (-0.98 to -0.51) |
| Libya | 2192.89 (1108.65-3479.87) | 94.76 (43.06-152.48) | 5876.08 (3050.74-8855.63) | 99.90 (51.91-151.14) | 0.23 (0.13 to 0.34) |
| Lithuania | 1868.72 (859.14-2901.42) | 43.30 (20.01-67.48) | 755.29 (356.95-1212.74) | 21.34 (10.28-33.48) | -2.62 (-2.83 to -2.41) |
| Luxembourg | 432.35 (191.30-712.55) | 93.23 (42.14-153.00) | 458.86 (203.75-750.36) | 59.44 (27.37-96.04) | -1.70 (-1.83 to -1.56) |
| Madagascar | 5332.38 (2991.07-7898.23) | 62.35 (31.40-98.27) | 14098.10 (6930.08-22437.56) | 75.95 (33.06-131.03) | 0.48 (0.40 to 0.55) |
| Malawi | 4009.32 (2334.62-6103.64) | 51.26 (28.58-78.60) | 8048.62 (4514.16-12398.64) | 59.06 (30.31-96.43) | 0.15 (0.01 to 0.30) |
| Malaysia | 6957.11 (3320.79-10676.31) | 57.99 (26.11-89.55) | 10468.82 (5262.43-15936.74) | 34.05 (17.14-51.64) | -2.52 (-2.84 to -2.20) |
| Maldives | 65.49 (31.01-107.48) | 49.86 (21.65-82.37) | 89.77 (44.72-139.92) | 20.14 (10.45-30.93) | -3.29 (-3.42 to -3.17) |
| Mali | 4645.77 (2116.55-7440.68) | 93.37 (40.22-154.83) | 10129.34 (4314.49-18488.17) | 82.82 (33.65-155.73) | -0.33 (-0.51 to -0.15) |
| Malta | 280.69 (123.16-473.60) | 67.94 (30.23-114.61) | 305.40 (134.73-507.21) | 55.01 (25.08-89.67) | -0.89 (-0.95 to -0.83) |
| Marshall Islands | 52.62 (25.27-81.90) | 227.94 (102.53-363.69) | 75.67 (34.41-122.61) | 174.59 (77.09-287.17) | -0.93 (-1.06 to -0.80) |
| Mauritania | 1539.23 (771.68-2448.38) | 126.83 (58.75-205.88) | 2339.16 (1203.88-3612.32) | 80.15 (38.51-127.43) | -1.48 (-1.71 to -1.25) |
| Mauritius | 946.71 (439.97-1460.70) | 121.35 (55.10-188.31) | 754.79 (359.33-1126.80) | 46.79 (23.68-69.79) | -3.41 (-3.68 to -3.15) |
| Mexico | 32542.47 (18094.91-48695.73) | 55.08 (29.16-83.08) | 33034.81 (18322.72-50117.38) | 25.70 (14.37-38.94) | -2.65 (-2.82 to -2.48) |
| Micronesia (Federated States of) | 163.29 (77.38-259.43) | 265.33 (117.97-439.53) | 142.57 (68.58-224.66) | 171.77 (80.71-274.21) | -1.53 (-1.58 to -1.47) |
| Monaco | 19.63 (8.19-32.55) | 52.91 (23.76-86.44) | 23.48 (10.46-39.24) | 53.80 (25.64-88.62) | 0.05 (-0.01 to 0.11) |
| Mongolia | 868.17 (396.74-1378.49) | 70.50 (30.22-114.59) | 824.43 (411.24-1264.07) | 29.61 (14.47-45.69) | -3.57 (-3.85 to -3.30) |
| Montenegro | 168.15 (83.26-268.62) | 26.61 (13.35-42.43) | 205.65 (100.39-330.36) | 31.35 (16.35-49.19) | 0.54 (0.45 to 0.62) |
| Morocco | 13220.22 (6344.35-20943.28) | 82.31 (37.20-131.73) | 25452.18 (11625.76-39639.25) | 73.33 (33.89-114.35) | -0.41 (-0.43 to -0.39) |
| Mozambique | 4402.77 (2591.96-6670.36) | 39.21 (21.76-60.57) | 10323.04 (5608.80-16294.34) | 51.39 (24.12-85.43) | 1.18 (1.06 to 1.29) |
| Myanmar | 24193.74 (11236.69-39893.21) | 89.19 (40.63-148.85) | 32925.51 (14221.27-56691.87) | 64.51 (27.68-111.08) | -1.37 (-1.52 to -1.22) |
| Namibia | 865.22 (387.29-1549.35) | 114.90 (48.11-214.95) | 1804.02 (836.43-3113.40) | 112.16 (50.98-198.48) | -0.34 (-0.66 to -0.02) |
| Nauru | 17.52 (8.55-26.39) | 259.13 (115.97-404.93) | 15.89 (7.74-24.39) | 209.69 (97.30-334.69) | -0.81 (-1.22 to -0.41) |
| Nepal | 7352.96 (3417.81-12768.00) | 64.03 (27.95-115.64) | 16957.67 (7302.74-30334.44) | 66.79 (28.12-120.41) | 0.38 (0.11 to 0.64) |
| Netherlands | 11373.35 (4880.88-19042.27) | 65.94 (28.96-109.00) | 10656.68 (4747.71-17735.68) | 51.89 (24.13-85.73) | -0.77 (-1.05 to -0.50) |
| New Zealand | 6047.62 (2817.20-9623.16) | 169.60 (80.01-269.46) | 4293.97 (2137.66-6633.76) | 78.54 (40.55-122.00) | -2.44 (-2.81 to -2.07) |
| Nicaragua | 1629.32 (924.19-2437.63) | 56.74 (29.54-85.69) | 2279.09 (1209.88-3447.69) | 37.26 (19.75-56.24) | -1.68 (-1.79 to -1.56) |
| Niger | 2834.72 (1462.42-4485.34) | 67.71 (31.03-112.32) | 6063.03 (2949.91-10268.64) | 49.62 (21.79-89.98) | -0.97 (-1.20 to -0.74) |
| Nigeria | 30351.54 (15691.19-46688.41) | 54.36 (25.35-84.06) | 80143.05 (41797.32-122372.84) | 57.81 (28.47-88.74) | 0.04 (-0.01 to 0.08) |
| Niue | 3.09 (1.52-4.79) | 140.68 (67.78-217.87) | 1.96 (1.01-3.03) | 104.34 (55.97-159.30) | -1.50 (-1.65 to -1.35) |
| North Macedonia | 1940.98 (910.60-3065.65) | 102.88 (48.44-163.18) | 1641.11 (756.79-2654.26) | 58.52 (28.59-91.87) | -2.31 (-2.57 to -2.04) |
| Northern Mariana Islands | 33.71 (17.49-53.10) | 113.27 (55.18-183.76) | 30.87 (16.32-44.59) | 63.73 (34.28-90.92) | -2.11 (-2.34 to -1.87) |
| Norway | 5999.57 (2638.37-9734.85) | 110.23 (49.35-177.56) | 2882.79 (1285.77-4632.37) | 46.62 (22.11-73.98) | -3.16 (-3.30 to -3.01) |
| Oman | 663.66 (333.79-1048.41) | 65.74 (30.38-107.69) | 2174.10 (1123.66-3282.94) | 61.63 (31.92-91.69) | 0.05 (-0.08 to 0.18) |
| Pakistan | 31359.41 (14143.43-54748.98) | 49.07 (21.20-89.73) | 80680.76 (34732.61-149627.25) | 55.99 (23.18-110.91) | 0.31 (0.01 to 0.60) |
| Palau | 26.16 (13.29-40.62) | 227.06 (111.94-353.35) | 35.70 (17.75-53.87) | 175.74 (89.53-262.19) | -0.72 (-0.80 to -0.65) |
| Palestine | 895.35 (446.42-1388.55) | 84.01 (38.53-132.35) | 2104.70 (1108.16-3123.61) | 59.39 (30.81-87.62) | -1.26 (-1.43 to -1.08) |
| Panama | 841.64 (472.76-1279.62) | 41.80 (22.84-64.02) | 1423.89 (755.18-2206.64) | 33.18 (17.76-50.99) | -1.02 (-1.16 to -0.89) |
| Papua New Guinea | 3659.68 (1726.90-6307.73) | 163.16 (69.23-286.09) | 10031.40 (4595.25-18451.20) | 158.36 (68.05-305.66) | -0.10 (-0.16 to -0.04) |
| Paraguay | 1180.39 (676.88-1803.54) | 33.19 (17.98-50.56) | 2913.33 (1582.95-4468.04) | 42.60 (23.13-65.47) | 0.91 (0.87 to 0.94) |
| Peru | 10485.29 (6030.62-16212.92) | 45.77 (26.98-69.67) | 11917.67 (6598.02-18907.92) | 34.07 (18.96-54.35) | -1.03 (-1.22 to -0.84) |
| Philippines | 24308.72 (12161.91-36603.23) | 62.30 (29.29-95.39) | 63618.53 (31097.70-97117.88) | 65.96 (31.02-101.84) | 0.28 (0.09 to 0.47) |
| Poland | 58280.52 (26267.02-94154.96) | 135.58 (61.82-219.96) | 31586.44 (14635.30-50218.36) | 66.88 (33.21-103.83) | -2.45 (-2.93 to -1.97) |
| Portugal | 12781.63 (5568.57-20724.91) | 108.29 (47.41-177.02) | 10818.39 (4750.23-18006.77) | 80.57 (37.15-132.45) | -0.78 (-1.07 to -0.48) |
| Puerto Rico | 4466.57 (2321.71-6904.97) | 124.46 (64.93-192.28) | 3286.57 (1683.67-5041.04) | 103.09 (56.56-160.49) | -1.12 (-1.29 to -0.95) |
| Qatar | 181.45 (90.50-280.04) | 86.29 (41.28-131.71) | 1139.93 (571.40-1764.02) | 52.29 (27.61-76.96) | -2.11 (-2.49 to -1.74) |
| Republic of Korea | 18800.70 (8981.47-29366.86) | 71.53 (31.69-114.38) | 12594.15 (5804.03-20475.49) | 19.24 (9.68-30.38) | -4.99 (-5.27 to -4.71) |
| Republic of Moldova | 2603.54 (1189.59-4042.14) | 56.75 (26.10-88.11) | 932.45 (445.36-1479.14) | 21.28 (10.31-33.69) | -3.76 (-3.94 to -3.58) |
| Romania | 16051.02 (7054.54-25260.75) | 60.29 (26.81-94.84) | 7905.12 (3678.78-12721.05) | 34.15 (16.22-54.06) | -1.83 (-1.94 to -1.73) |
| Russian Federation | 125513.31 (58145.54-194367.08) | 71.09 (33.41-109.50) | 36500.29 (17773.33-56254.39) | 21.30 (10.71-33.22) | -4.75 (-5.01 to -4.50) |
| Rwanda | 5222.73 (2937.63-8023.18) | 92.54 (48.50-146.45) | 7239.18 (3911.17-11374.91) | 66.49 (34.74-113.91) | -1.92 (-2.21 to -1.63) |
| Saint Kitts and Nevis | 21.22 (11.28-31.97) | 55.63 (28.94-84.50) | 29.61 (15.50-44.68) | 50.55 (28.49-75.93) | -0.38 (-0.48 to -0.29) |
| Saint Lucia | 81.76 (40.89-126.82) | 77.56 (38.03-121.69) | 136.00 (67.07-209.34) | 67.33 (34.86-103.05) | -0.75 (-0.89 to -0.61) |
| Saint Vincent and the Grenadines | 38.32 (20.08-58.84) | 43.42 (21.58-67.98) | 60.37 (30.74-92.64) | 49.11 (25.86-75.52) | 0.26 (0.09 to 0.44) |
| Samoa | 210.73 (101.47-370.01) | 206.63 (92.93-383.40) | 240.20 (131.05-375.45) | 149.20 (76.61-236.00) | -1.01 (-1.08 to -0.94) |
| San Marino | 13.54 (5.94-22.44) | 49.89 (22.63-81.83) | 20.21 (8.94-33.22) | 52.33 (24.61-84.84) | 0.28 (0.24 to 0.33) |
| Sao Tome and Principe | 115.76 (56.72-186.64) | 161.37 (74.87-265.39) | 213.89 (100.83-351.98) | 150.48 (69.89-251.25) | -0.45 (-0.65 to -0.26) |
| Saudi Arabia | 8298.54 (4355.12-12778.91) | 103.73 (49.23-165.49) | 25026.72 (12543.19-38083.30) | 85.74 (44.63-130.23) | -0.70 (-0.81 to -0.59) |
| Senegal | 3239.84 (1674.18-5212.51) | 76.94 (35.09-127.06) | 5949.44 (2968.83-9839.78) | 60.11 (28.29-99.83) | -0.55 (-0.91 to -0.19) |
| Serbia | 5617.62 (2621.50-8716.86) | 53.64 (25.31-83.52) | 4434.86 (2153.47-6861.97) | 35.76 (18.23-54.82) | -1.80 (-1.99 to -1.61) |
| Seychelles | 36.07 (16.51-54.90) | 60.42 (27.25-91.97) | 53.97 (26.15-81.71) | 47.52 (23.63-71.38) | -0.80 (-0.90 to -0.70) |
| Sierra Leone | 1810.60 (979.20-2868.95) | 66.40 (31.51-109.68) | 3554.13 (1720.17-5846.64) | 62.82 (28.08-109.18) | 0.05 (-0.13 to 0.23) |
| Singapore | 1193.02 (533.18-1929.18) | 42.25 (19.40-67.81) | 1322.17 (565.00-2212.66) | 19.73 (9.15-32.15) | -2.96 (-3.30 to -2.62) |
| Slovakia | 1853.16 (902.07-2901.87) | 32.43 (15.86-50.50) | 1551.07 (719.83-2511.95) | 23.01 (11.11-36.74) | -1.22 (-1.32 to -1.12) |
| Slovenia | 1606.17 (736.07-2554.71) | 69.83 (33.16-110.56) | 1153.98 (543.18-1862.74) | 44.28 (21.38-70.27) | -1.65 (-1.80 to -1.50) |
| Solomon Islands | 197.41 (91.43-332.74) | 115.78 (51.37-200.98) | 476.79 (225.49-801.71) | 109.76 (49.34-188.32) | -0.13 (-0.19 to -0.07) |
| Somalia | 3533.74 (1870.66-5701.38) | 72.22 (33.32-121.21) | 9055.03 (4904.40-14196.49) | 73.86 (34.26-125.56) | -0.04 (-0.14 to 0.06) |
| South Africa | 34064.09 (16660.09-52581.62) | 126.40 (57.78-200.63) | 51656.32 (24576.84-76754.64) | 101.16 (48.04-149.19) | -0.92 (-1.47 to -0.37) |
| South Sudan | 2112.06 (1198.18-3410.78) | 44.10 (23.79-70.32) | 3096.36 (1766.85-4960.12) | 41.74 (21.74-71.82) | -0.35 (-0.71 to 0.01) |
| Spain | 23290.95 (11086.30-38395.33) | 50.11 (23.85-82.61) | 23715.48 (10895.36-37753.72) | 40.00 (18.72-63.87) | -0.63 (-0.81 to -0.45) |
| Sri Lanka | 7869.05 (3578.48-12341.88) | 64.18 (27.92-101.32) | 10755.34 (4537.90-17888.27) | 41.94 (18.22-69.65) | -1.62 (-1.78 to -1.45) |
| Sudan | 14570.54 (5870.65-30130.04) | 134.53 (49.18-297.03) | 22181.83 (10544.18-37134.97) | 89.65 (40.85-156.55) | -1.59 (-1.68 to -1.49) |
| Suriname | 129.78 (64.32-204.79) | 39.24 (19.17-61.90) | 247.87 (127.13-377.97) | 40.86 (21.12-61.68) | -0.11 (-0.22 to 0.00) |
| Sweden | 12031.34 (5210.41-20207.49) | 107.89 (47.22-180.71) | 7106.31 (3068.40-11703.78) | 58.99 (26.84-97.17) | -1.92 (-2.36 to -1.48) |
| Switzerland | 5605.49 (2510.71-9048.10) | 66.03 (30.53-105.28) | 4206.40 (1837.40-6945.36) | 39.46 (18.21-64.76) | -1.79 (-1.92 to -1.65) |
| Syrian Arab Republic | 7482.00 (3931.16-10996.79) | 106.95 (50.87-166.44) | 14905.16 (8293.88-21880.51) | 112.61 (61.62-167.68) | 0.03 (-0.13 to 0.19) |
| Taiwan (Province of China) | 9008.23 (4170.07-13895.85) | 55.39 (25.77-86.05) | 10879.65 (5124.41-17399.05) | 39.69 (20.40-62.57) | -1.15 (-1.49 to -0.81) |
| Tajikistan | 2440.69 (1132.31-3886.37) | 83.25 (37.01-135.93) | 3105.19 (1561.28-4926.41) | 48.31 (22.27-77.53) | -2.06 (-2.27 to -1.86) |
| Thailand | 15587.84 (7291.46-24229.33) | 36.74 (16.81-56.38) | 27548.41 (13343.96-43641.43) | 32.08 (16.28-49.56) | -0.89 (-1.07 to -0.70) |
| Timor-Leste | 151.29 (76.28-239.82) | 28.54 (14.33-47.74) | 324.92 (175.27-494.19) | 30.00 (14.19-47.89) | 0.13 (-0.11 to 0.37) |
| Togo | 1334.52 (675.28-2158.65) | 74.84 (35.58-126.93) | 4016.87 (1906.28-6914.51) | 75.53 (34.00-134.68) | 0.20 (0.07 to 0.34) |
| Tokelau | 2.09 (0.90-3.60) | 155.53 (65.48-274.30) | 1.55 (0.81-2.39) | 108.64 (56.40-166.90) | -1.59 (-1.73 to -1.46) |
| Tonga | 104.01 (56.24-157.04) | 154.43 (76.81-240.01) | 113.62 (63.07-167.25) | 128.25 (68.40-190.98) | -0.57 (-0.67 to -0.46) |
| Trinidad and Tobago | 609.20 (318.43-921.67) | 60.34 (30.76-92.26) | 723.63 (372.38-1079.47) | 45.84 (24.54-68.74) | -1.16 (-1.46 to -0.85) |
| Tunisia | 3794.08 (1884.34-5740.55) | 68.03 (31.33-103.94) | 7758.38 (3857.44-11992.50) | 61.36 (30.64-94.28) | -0.57 (-0.64 to -0.51) |
| Turkey | 36241.53 (18496.13-55581.51) | 86.94 (42.35-135.37) | 56168.11 (28428.71-85326.67) | 63.52 (32.74-95.28) | -1.34 (-1.53 to -1.15) |
| Turkmenistan | 1695.57 (799.14-2676.82) | 70.95 (31.25-113.69) | 1076.18 (548.62-1694.57) | 22.04 (11.10-34.93) | -4.47 (-4.86 to -4.08) |
| Tuvalu | 17.29 (8.12-27.88) | 229.76 (103.99-369.01) | 14.97 (6.90-23.62) | 138.90 (63.80-222.57) | -1.65 (-1.71 to -1.60) |
| Uganda | 6161.24 (3542.38-9696.25) | 49.18 (24.70-81.21) | 16015.33 (9060.80-26151.58) | 52.08 (25.70-97.50) | -0.32 (-0.50 to -0.15) |
| Ukraine | 22070.11 (9868.14-35498.19) | 35.00 (15.64-56.48) | 8628.27 (3978.57-13697.56) | 18.44 (9.01-29.41) | -2.73 (-2.93 to -2.53) |
| United Arab Emirates | 2013.41 (976.81-3158.28) | 180.76 (85.80-284.24) | 14256.79 (7086.35-23213.22) | 153.30 (78.69-233.42) | -0.19 (-0.42 to 0.04) |
| United Kingdom | 90927.41 (41366.16-149939.17) | 137.96 (63.90-225.35) | 72588.63 (33244.20-117699.19) | 94.57 (44.56-151.59) | -1.24 (-1.43 to -1.06) |
| United Republic of Tanzania | 9140.07 (5250.39-13852.90) | 51.62 (24.57-80.85) | 26906.11 (14098.56-42089.15) | 62.69 (28.91-104.10) | 0.53 (0.49 to 0.58) |
| United States Virgin Islands | 87.66 (48.06-130.06) | 86.43 (46.85-128.80) | 65.97 (34.94-97.80) | 75.62 (42.50-113.66) | -0.42 (-0.45 to -0.39) |
| United States of America | 225464.34 (107973.02-352664.31) | 83.54 (41.60-128.82) | 389253.62 (187273.68-606143.36) | 104.26 (51.31-160.04) | 1.35 (1.04 to 1.66) |
| Uruguay | 2518.16 (1224.05-3986.47) | 71.68 (35.10-112.89) | 2305.12 (1131.06-3588.33) | 55.51 (27.98-85.79) | -1.01 (-1.07 to -0.95) |
| Uzbekistan | 17500.29 (8397.30-27426.43) | 138.50 (65.01-220.17) | 17547.96 (8512.83-27692.26) | 58.13 (27.99-91.91) | -3.87 (-4.44 to -3.29) |
| Vanuatu | 125.14 (52.65-261.55) | 153.53 (60.69-330.48) | 303.51 (129.44-592.40) | 143.71 (59.00-293.90) | -0.46 (-0.57 to -0.36) |
| Venezuela (Bolivarian Republic of) | 8247.25 (4737.13-12331.03) | 50.80 (27.67-76.73) | 11046.46 (5903.26-16818.64) | 40.91 (22.14-63.29) | -1.03 (-1.26 to -0.79) |
| Viet Nam | 7514.47 (3798.61-12382.27) | 16.46 (7.55-27.61) | 18965.69 (8827.94-30733.46) | 18.90 (8.96-30.66) | 0.68 (0.54 to 0.83) |
| Yemen | 6653.59 (3177.10-10546.58) | 99.09 (40.66-172.77) | 16842.99 (8088.88-26427.49) | 91.54 (38.57-150.36) | -0.50 (-0.65 to -0.34) |
| Zambia | 2669.81 (1528.94-3937.47) | 47.50 (25.29-72.40) | 7082.23 (3775.59-11477.12) | 57.41 (27.54-99.13) | 0.39 (0.24 to 0.54) |
| Zimbabwe | 3237.09 (1523.42-5166.41) | 67.20 (28.94-108.85) | 11857.93 (5048.86-20156.19) | 139.95 (56.99-237.10) | 3.01 (2.55 to 3.48) |

Abbreviations: DALYs = disability-adjusted life years; ASDR = age-standardized DALY rate; UI = uncertainty interval; CI = confidence interval; EAPC = estimated annual percentage change.
