# Supplementary material for: The influence of high body mass index on asthma: A study integrating GBD 2021 and Mendelian randomization
Source: Medicine (Baltimore). 2026 Nov 6;105(45):e51490. doi: 10.1097/MD.0000000000051490 (PMC13747491; doi:10.1097/MD.0000000000051490)
Supplement: Supplementary file 4 [file medi-105-e51490-s005.docx]

**Table S4.**  Contributions of population growth, population aging, and epidemiological change to changes in asthma deaths and DALYs attributable to high BMI by SDI quintile, 1990–2021.

| **Measure** | **SDI region** | **Overall change 1990–2021** | **Population growth** | | **Population aging** | | **Epidemiological change** | |
| --- | --- | --- | --- | --- | --- | --- | --- | --- |
|  |  |  | **n** | **%** | **n** | **%** | **n** | **%** |
| Deaths | Global | 24322.68 | 19663.59 | 80.84 | 20360.69 | 83.71 | -15701.6 | -64.56 |
| Deaths | Low SDI | 4758.21 | 4902.33 | 103.03 | 214.87 | 4.52 | -358.99 | -7.54 |
| Deaths | Low-middle SDI | 14711.48 | 8041.27 | 54.66 | 6124.03 | 41.63 | 546.18 | 3.71 |
| Deaths | Middle SDI | 9346.55 | 4973.75 | 53.21 | 9086.66 | 97.22 | -4713.86 | -50.43 |
| Deaths | High-middle SDI | -1396.81 | 1396.28 | -99.96 | 3747.26 | -268.27 | -6540.35 | 468.23 |
| Deaths | High SDI | -3099.45 | 1512.01 | -48.78 | 3016.79 | -97.33 | -7628.25 | 246.12 |
| DALYs | Global | 1029358.36 | 1091841.98 | 106.07 | 622035.94 | 60.43 | -684519.56 | -66.5 |
| DALYs | Low SDI | 224779.53 | 223663.51 | 99.5 | 3675.66 | 1.64 | -2559.64 | -1.14 |
| DALYs | Low-middle SDI | 477569.44 | 301902.34 | 63.22 | 152239.16 | 31.88 | 23427.94 | 4.91 |
| DALYs | Middle SDI | 364396.37 | 226260.94 | 62.09 | 215087.04 | 59.03 | -76951.61 | -21.12 |
| DALYs | High-middle SDI | -55598.36 | 91481.2 | -164.54 | 130242.56 | -234.26 | -277322.12 | 498.8 |
| DALYs | High SDI | 18106.51 | 172909.54 | 954.96 | 114965.83 | 634.94 | -269768.86 | -1489.9 |

Abbreviations: SDI = Socio-Demographic Index; DALYs = disability-adjusted life years. n = change in number of deaths/DALYs attributable to each factor, 1990–2021; % = percentage contribution of each factor to the overall change.
